# A systematic comparison of single-cell perturbation response prediction models

**DOI:** 10.1126/sciadv.aed3414

**Published:** 2026-09-09

**Authors:** Lanxiang Li, Yue You, Yunlin Fu, Wenyu Liao, Xueying Fan, Shihong Lu, Ye Cao, Bo Li, Wenle Ren, Jiaming Kong, Shuangjia Zheng, Jizheng Chen, Xiaodong Liu, Luyi Tian

**Affiliations:** ^1^ GMU-GIBH Joint School of Life Sciences, Guangzhou Medical University, Guangzhou, China.; ^2^ Guangzhou National Laboratory, Guangzhou, China.; ^3^ The University of Hong Kong, Hong Kong, China.; ^4^ School of Life Sciences, Westlake University, Hangzhou, China.; ^5^ Research Center for Industries of the Future, Westlake University, Hangzhou, China.; ^6^ Westlake Laboratory of Life Sciences and Biomedicine, Hangzhou, China.; ^7^ Westlake Institute for Advanced Study, Hangzhou, China.; ^8^ Renaissance Era Innovation Technology Co., Ltd., Beijing, China.; ^9^ Global Institute of Future Technology, Shanghai Jiao Tong University, Shanghai, China.; ^10^ State Key Laboratory of Respiratory Disease, National Clinical Research Center for Respiratory Disease, Guangzhou Institute of Respiratory Health, The First Affiliated Hospital of Guangzhou Medical University, Guangzhou, China.

## Abstract

Predicting single-cell transcriptional responses to perturbations is central to dissecting gene regulation and accelerating therapeutic design, yet the field lacks a rigorous, task-spanning assessment of model behavior. We present a large-scale benchmark of 13 representative methods and baselines across 25 datasets spanning diverse perturbation modalities and species, including two primary immune-cell drug-response resources. We evaluated three core tasks—generalization to unseen single-gene perturbations, prediction of combinatorial interactions, and transfer across cell types—using 24 metrics covering expression-level accuracy, relative changes, differential expression (DE) recovery, and distributional similarity. Across tasks, performance depended strongly on perturbation effect size and evaluation perspective: Expression-level agreement was the highest for small-effect perturbations resembling controls, whereas delta- and DE-based metrics improved with larger effects, providing clearer signals. Models shared a conservative bias, with fine-tuned foundation models compressing variance and underestimating synergistic effects in combinations. PerturbNet showed superior recovery of DE signatures in Tasks 1 and 2, while no method consistently generalized across cell types in Task 3, where biological consistency dominated outcomes. This benchmark establishes current methodological limits, clarifies that different metrics probe distinct biological signals rather than redundant summaries of the same prediction problem, and provides a foundation for developing virtual-cell models that more faithfully capture heterogeneous perturbation responses.

## INTRODUCTION

Single-cell RNA sequencing (scRNA-seq) offers unprecedented resolution for exploring cellular heterogeneity (*1*, *2*). Multiplexed perturbations together with scRNA-seq, such as Perturb-seq and CROP-seq, help uncover how cells respond to diverse stimuli or interventions (*3*–*5*). These approaches advance our understanding of cellular functions and regulatory networks, providing crucial insights for drug development and disease treatment (*6*). However, current experimental systems are largely optimized for cell lines, with limited applicability to primary or in vivo systems. Furthermore, combinatorial multiplexing experiments remain prohibitively expensive and technically challenging to scale (*7*, *8*).

In this context, in silico approaches that predict postperturbation cell states across diverse environments and conditions present a promising, scalable alternative and provide a path to building a virtual cell (*9*, *10*). Early approaches focused on predicting cellular responses in cell types absent from experimental training data or responses to combinations of previously tested perturbations (*11*–*13*). However, these methods could not be generalized to unseen perturbations. Recent advances have incorporated graph-based methods and foundational models that take advantage of prior knowledge of gene expression relationships, resulting in improved generalizability (*14*–*17*).

Among them, single-cell foundation models represent a pivotal breakthrough. By leveraging vast datasets and pretrained transformer architectures (*18*), these models learn robust, generalizable relationships between genes and have demonstrated potential across diverse downstream tasks, including transcriptome prediction following perturbations after models are fine-tuned on specific datasets (*19*). Despite their promise, the field lacks a comprehensive framework to evaluate their capabilities systematically. Compared to tasks such as cell annotation and batch correction, perturbation prediction offers experimentally generated model input/output with well-defined objectives, making it a more robust benchmark for assessing foundation models (*15*, *20*, *21*). Recent studies have provided initial benchmarks comparing foundation models, other deep learning architectures, and simple baselines for perturbation effect prediction and reported that complex models do not consistently outperform the simpler alternatives (*22*–*26*). While this work provides valuable insights, its evaluation was limited in dataset diversity and task coverage, leaving the question of whether such conclusions generalize across broader biological contexts.

At the same time, the Virtual Cell Challenge (*27*) has emerged as a community benchmark for perturbation prediction, attracting wide participation and fostering the development of evaluation frameworks such as Cell-Eval (*28*). Together with the release of large-scale datasets like Tahoe-100M (*29*), these initiatives reflect a growing momentum toward standardized, transparent, and multimetric benchmarking in this rapidly evolving field.

Motivated by these developments, we present a comprehensive benchmark that substantially expands the scope to a more diverse set of datasets, perturbation modalities, and prediction tasks. In addition to systematically evaluating model performance, we summarize common patterns across datasets and identify key biological and technical factors influencing predictive accuracy—most notably, that the magnitude of the perturbation effect strongly affects both generalization and double perturbation prediction performance and that, in cell-type transfer settings, the greater transcriptomic distance between source and target cell types is associated with reduced accuracy.

## RESULTS

### Benchmarking models and experimental design

**Fig. 1.**
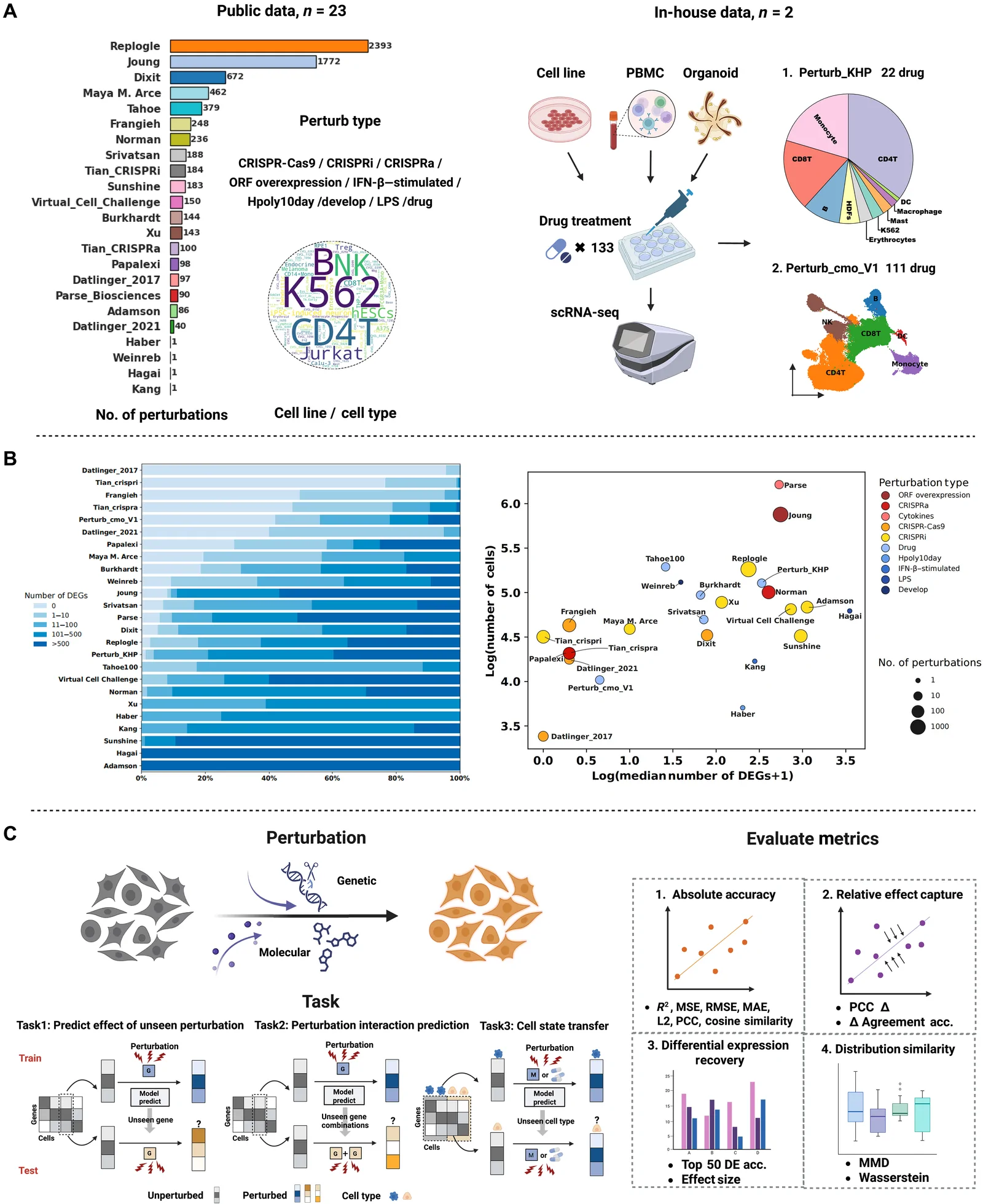
Benchmarking datasets, perturbation properties, and experimental design. (**A**) Data overview. The benchmarking collection integrates 23 public datasets and 2 in-house datasets. Public resources include large-scale CRISPR screens, drug perturbation atlases, and immune stimulation studies (numbers shown in the figure denote the total number of perturbations in the original datasets). The in-house datasets profile the effects of 133 drug treatments across lymphoid immune subsets, enabling systematic evaluation of cross-cell-type generalization. Together, these datasets cover a wide range of perturbation types [CRISPR-Cas9, CRISPRi, CRISPRa, open reading frame (ORF) overexpression, drug, IFN-β, Hpoly, and LPS] across diverse cellular systems, including cancer cell lines, pluripotent stem cells, PBMCs, organoids, and nonhuman immune cells. (**B**) Perturbation effect size and dataset properties. Left: Distribution of dataset components by percentage of DEGs per perturbation compared to controls. Right: Scatterplot of the median number of DEGs per condition (*x* axis) versus the log number of cells (*y* axis), summarizing the number of DEGs across all perturbations in each dataset (including both training and testing conditions). Point size indicates the number of perturbations, and color encodes perturbation type, ranging from red (strongest transcriptional effects) to blue (weakest effects). (**C**) Benchmarking tasks and evaluation strategy. Both genetic and molecular perturbations are used in benchmarking. Models were trained on observed perturbation-response pairs and evaluated on held-out conditions. Three tasks were benchmarked: Task 1, prediction of responses to unseen perturbations; Task 2, prediction of combinatorial perturbation interactions; Task 3, transfer of responses across unseen cell types. Performance was assessed using multiple complementary metrics to assess absolute accuracy, relative effect capture, DE recovery, and distributional similarity. Created in BioRender. L. Tian (2026), https://biorender.com/me28fg3.

We comprehensively benchmarked 13 single-cell perturbation response prediction models across a broad and diverse dataset collection, alongside a suite of baselines for different tasks. Our goal was to assess how well different model architectures generalize across biological contexts, perturbation types, and prediction tasks (fig. S1). Our dataset collection spans a broad range of biological contexts, perturbation modalities, and effect sizes. It integrates large-scale public resources, recently released high-throughput perturbation screens, and in-house–generated datasets. The collection covers genetic perturbations [e.g., CRISPR knockouts (KOs) and activations], small-molecule treatments (e.g., kinase inhibitors and antiaging compounds), and immune stimuli [e.g., lipopolysaccharide (LPS) and cytokines], measured across cancer cell lines (e.g., K562 and A549), pluripotent stem cells [e.g., human embryonic stem cells (hESCs)], and primary immune populations (e.g., T cells, natural killer cells, and monocytes), as well as nonhuman immune cells (e.g., mouse and rat). Notably, we incorporated datasets that include multicell-type profiling from the Virtual Cell Challenge, extensive drug screens across dozens of cell lines, and primary immune cell responses to cytokines. In addition, we generated two datasets that capture the effects of drugs on both myeloid and lymphoid subsets, enabling a more systematic evaluation of cell-type transfer (Fig. 1A).

We systematically examined perturbation modalities, numbers of differentially expressed genes (DEGs), E-distances, and sample sizes both across experiments and within individual experiments. This analysis revealed broad dispersion in the number of perturbations, cell numbers, and perturbation effect sizes, with substantial heterogeneity not only between studies but also among conditions within the same study. While perturbation modality may contribute to transcriptional response variation, well-designed, well-powered datasets—such as the Replogle genome-scale screen in RPE1 cells and the curated Virtual Cell Challenge—can exhibit strong signals (often >500 DEGs for many conditions) (Fig. 1B). Beyond modality, cell-type sensitivity further modulates measurable effects, with certain immune subsets showing muted transcriptional responses to specific perturbations compared with other cell types. A full characterization of these properties and details of all datasets used are summarized in Table 1 and table S1. This heterogeneity in effect size and response patterns enables a more comprehensive and discriminative assessment of model generalization across diverse biological scenarios.

**Table 1.** Overview of datasets used in this study. Ctrl, control.

Species	Task	Source paper	Data name	Perturbation type	n_obs × n_vars	No. of perturbations (including ctrl)	No. of perturbations	Sub_Perturbation type	Sub_n_obs × n_vars	Sub_No. of perturbations (including ctrl)	Sub_No. of perturbations
Human	Task 1	Datlinger	DatlingerBock2017.h5ad	CRISPR-Cas9 + TCR	5905 × 36,722	97	96	CRISPR-Cas9	2419 × 5018	24	23
Human	Task 1	Datlinger	DatlingerBock2021.h5ad	CRISPR-Cas9 + TCR	39,194 × 25,904	41	40	CRISPR-Cas9	17,723 × 5012	21	20
Human	Task 1	Adamson	Adamson.h5ad	CRISPRi	68,603 × 5060	87	86	CRISPRi	68,603 × 5060	87	86
Human	Task 1	Joung	Fengzhang2023.h5ad	ORF overexpression	1,145,823 × 37,528	1773	1772	ORF overexpression	755,474 × 5895	1079	1078
Human	Task 1	Frangieh	FrangiehIzar2021_RNA.h5ad	CRISPR-Cas9 + IFN-γ	218,331 × 23,712	249	248	CRISPR-Cas9	42,861 × 4065	211	210
Human	Task 1	Papalexi	PapalexiSatija2021_eccite_RNA.h5ad	CRISPR-Cas9	20,729 × 18,649	99	98	CRISPR-Cas9	20,681 × 3674	95	94
Human	Task 1	Replogle	ReplogleWeissman2022_rpe1.h5ad	CRISPRi	247,914 × 8749	2394	2393	CRISPRi	183,126 × 2994	1378	1377
Human	Task 1	Tian	TianKampmann2021_CRISPRi.h5ad	CRISPRi	32,300 × 33,538	185	184	CRISPRi	31,815 × 5170	181	180
Human	Task 1	Tian	TianKampmann2021_CRISPRa.h5ad	CRISPRa	21,193 × 33,538	101	100	CRISPRa	20,720 × 5053	96	95
Human	Task 1	Xu	Junyue_Cao.h5ad	CRISPRi	186,375 × 42,235	144	143	CRISPRi	77,654 × 20,000	88	87
Human	Task 1	Virtual Cell Challenge	vcc_train_filtered.h5ad	CRISPRi	221,273 × 18,080	151	150	CRISPRi	64,879 × 5000	51	50
Human	Task 1/2	Dixit	DixitRegev2016.h5ad	CRISPR-Cas9	51,898 × 23,529	673	672	CRISPR-Cas9	33,144 × 5008	61	60
Human	Task 1/2	Norman	NormanWeissman2019_filtered.h5ad	CRISPRa	111,445 × 33,694	237	236	CRISPRa	100,792 × 2561	232	231
Human	Task 1/2	Sunshine	Sunshine2023_CRISPRi_sarscov2.h5ad	CRISPRi	90,380 × 33,549	184	183	CRISPRi	32,489 × 5117	170	169
Human	Task 1/2	Maya M. Arce	Arce_MM_CRISPRi.h5ad	CRISPRi	178,960 × 36,807	463	462	CRISPRi	39,045 × 5000	47	46
Human	Task 3	Kang	Kang.h5ad	IFN-β–stimulated	16,893 × 6998	2	1	IFN-β–stimulated	16,893 × 6998	2	1
Mouse	Task 3	Haber	Haber.h5ad	Hpoly10day	5059 × 7000	2	1	Hpoly10day	5059 × 7000	2	1
Mouse	Task 3	Weinreb	Weinreb.h5ad	Develop	130,861 × 1622	2	1	Develop	130,861 × 1622	2	1
Cross-species	Task 3	Hagai	Hagai.h5ad	LPS	62,114 × 6619	2	1	LPS	62,114 × 6619	2	1
Human	Task 3	Srivatsan	Srivatsan_sciplex3_sub10.h5ad	Drug	799,317 × 110,984	189	188	Drug	49,660 × 5000	11	10
Human	Task 3	Burkhardt	Burkhardt_sub10.h5ad	Drug	240,090 × 21,255	145	144	Drug	93,636 × 4586	11	10
Human	Task 3	Tahoe	Tahoe100_sub10.h5ad	Drug	100,648790 × 62,710	380	379	Drug	194,700 × 5000	11	10
Human	Task 3	Parse Biosciences	Parse_10M_PBMC_sub10.h5ad	Cytokines	9,697,974 × 40,352	91	90	Cytokines	1,637,321 × 5000	11	10
Human	Task 3	This study	Perturb_KHP_sub10.h5ad	Drug	262,565 × 23,409	23	22	Drug	127,595 × 5000	11	10
Human	Task 3	This study	Perturb_cmo_V1_sub10.h5ad	Drug	112,566 × 34,868	112	111	Drug	10,410 × 5000	11	10

In terms of the benchmarking object, we categorized them into three tasks focusing on their ability in different scenarios, each representing a different aspect of generalization: predicting unseen perturbations, combinatorial perturbation interactions, and responses across unseen cell types. These tasks reflect the varying complexity of perturbation prediction and the diverse experimental contexts in which these models are applied. The evaluation process is shown in Fig. 1C, where the input and output for the training and testing phases are outlined. In particular, we focus on the following:

1) Unseen perturbation prediction (Fig. 1C, bottom left): In this task, the models were trained to predict the effects of unseen single-gene perturbations within the same cell type or cell line. Using training data that included both unperturbed and known single-gene perturbation responses, the models were tasked with inferring gene expression changes under previously unseen perturbations.

2) Combinatorial perturbation prediction (Fig. 1C, bottom middle): This task introduced additional complexity by requiring the models to predict the interaction effects of combinatorial perturbations. On the basis of single-gene perturbation data, models were expected to predict how previously unseen combinations of gene perturbations would influence gene expression in the same cell type or cell line.

3) Cell state transfer (Fig. 1C, bottom right): This task assessed the models’ ability to generalize the effect of a known perturbation across different cell types or other categories. The models were trained on unperturbed and perturbed data from various cell types to learn gene expression patterns under specific perturbations. They were then tested on previously unseen cell types, predicting their responses to the same perturbations.

To evaluate the models in the three tasks, we selected 13 representative models categorized into four types on the basis of their design and model structure: (i) foundation models (scGPT and scFoundation), pretrained on large datasets; (ii) gene regulatory network (GRN)–based models (GEARS and scELMo), which use regulatory information to predict perturbation effects; (iii) variational autoencoder (VAE)–based model (PerturbNet, scGen, trVAE, scVIDR, and scPRAM); (iv) other deep learning–based models [Compositional Perturbation Autoencoder (CPA), scPreGAN, BioLord, and Squidiff]. In addition, we established a range of task-specific baselines for comparison, including simple statistical baselines (context-mean and perturbation-mean predictors) and model-based baselines [linear regression and multilayer perceptron (MLP)–based neural network models]. Detailed baseline descriptions are provided in Materials and Methods (see the “Baseline model” section), and model descriptions are provided in table S2.

Next, we implemented a standardized, multidimensional evaluation pipeline designed to capture complementary aspects of model performance across diverse perturbation scenarios (Fig. 1C, right). Our framework integrates four categories of metrics that together assess absolute accuracy, relative effect capture, differential expression (DE) recovery, and distributional similarity. Absolute gene expression–level agreement was quantified using error-based measures [mean squared error (MSE), root mean squared error (RMSE), mean absolute error (MAE), and *L*_2_ loss], goodness of fit (*R*^2^), and correlation metrics (Pearson’s correlation and cosine similarity). Each of these metrics was calculated on the full gene set as well as on subsets containing the top 20, 50, or 100 genes ranked by absolute score, allowing us to assess model behavior across different levels of gene prioritization. To evaluate the ability to capture relative perturbation effects, we computed expression changes (perturbed versus control) and assessed Pearson’s correlation on these delta values, together with delta direction accuracy, which measures the proportion of genes with correctly predicted up- or down-regulation. We further evaluated the recovery of DE by identifying DEGs in both predicted and observed profiles and then quantifying the overlap accuracy and the Spearman correlation of effect size, defined as the number of DEGs per perturbation. Last, to assess higher-order similarity beyond mean shifts, we calculated the Wasserstein distance and maximum mean discrepancy (MMD) between predicted and observed gene expression values. Both metrics were computed per gene by comparing the distributions of predicted and observed expression values across all cells for that gene. The per-gene distances were then aggregated to yield a single scalar that summarizes the overall distributional fidelity for each perturbation condition. In addition to absolute metric values, we performed a ranking-based meta-analysis in which models were ranked for each perturbation and metric, and aggregated win rates were computed to capture relative performance trends across datasets and tasks. In summary, we systematically evaluated 13 representative methods and baseline models across three core tasks using 25 datasets and 24 distinct evaluation metrics to provide a comprehensive comparison of model performance.

### Prediction of unseen single-gene perturbations

**Fig. 2.**
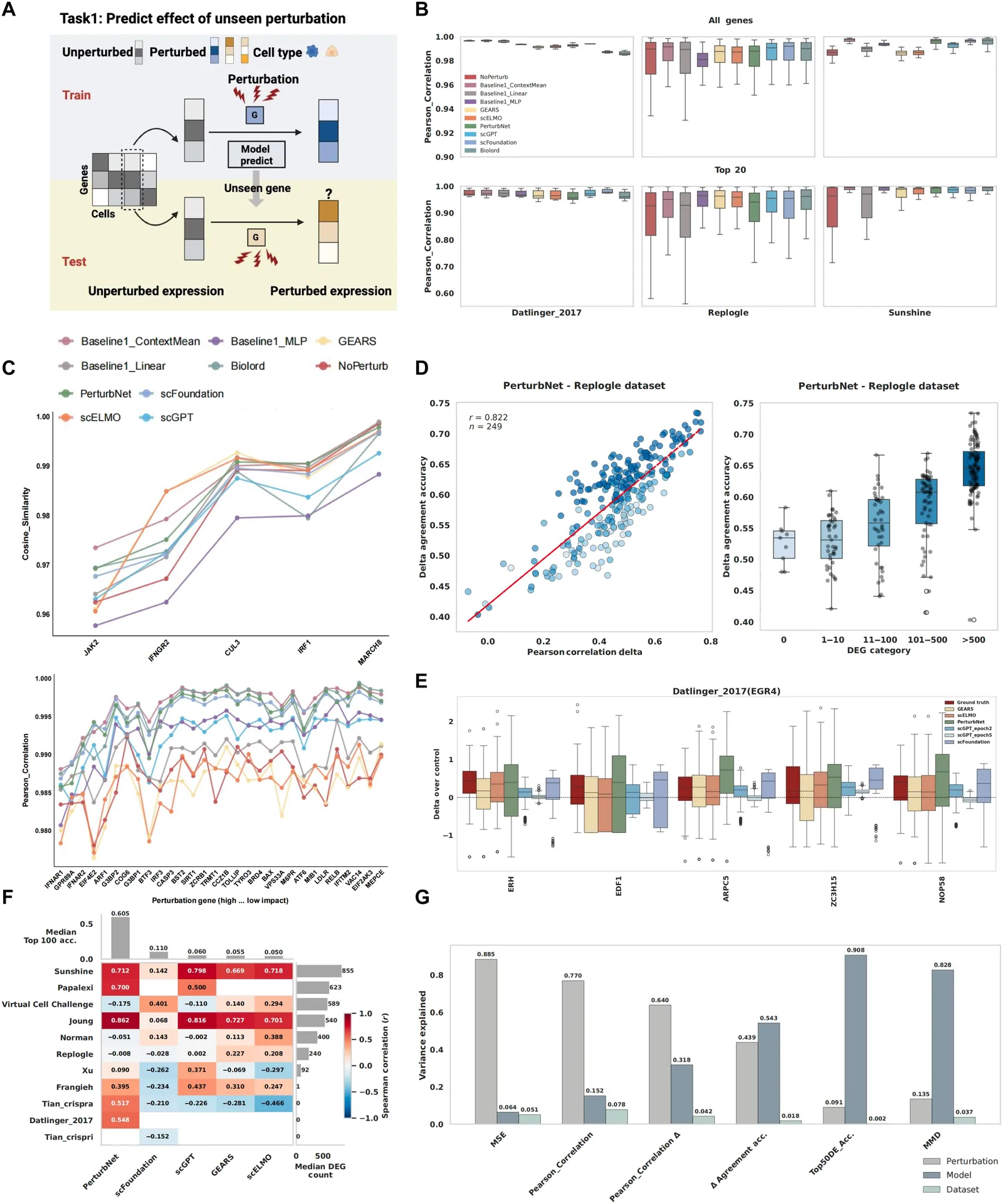
Prediction of unseen single-gene perturbations across multiple models and datasets. (**A**) Schematic overview of the single-gene perturbation prediction task. Created in BioRender. L. Tian (2026), https://biorender.com/bjpu41h. (**B**) Absolute gene expression–level performance metrics. Pearson’s correlation coefficients on all genes and the top 20 genes across different datasets and models. (**C**) Cosine similarity (Papalexi dataset) and Pearson’s correlation coefficients (Sunshine dataset) on selected perturbation genes across models. The perturbation genes are ranked by impact strength. (**D**) Delta-based analysis of perturbation effect size and prediction accuracy predicted by the PerturbNet model on the Replogle dataset. Pearson correlation and delta direction accuracy were positively correlated (left), with larger-effect perturbations yielding higher delta direction accuracy (right). (**E**) Distribution comparison of the top five genes between predicted and observed expression changes on EGR4 perturbation of the Datlinger 2017 dataset. (**F**) Correlation of effect sizes between true and predicted perturbations relative to control. All statistics shown are computed using the subset of Task 1 test perturbations shared by all models. The median overlap accuracy of the top 100 DEGs across datasets is shown above, and the median DEG count per dataset is shown on the right. Models are ranked by overlap accuracy and datasets by median effect size. (**G**) Variance decomposition analysis showing the relative contribution of perturbation identity, model choice, and dataset identity to prediction performance across different evaluation metrics.

In the first task, we assessed the ability of each model to generalize to single-gene perturbations that were not observed during training, a critical measure of whether models can extrapolate to previously unseen genetic interventions (Fig. 2A). GEARS and scELMo leverage gene regulatory priors, foundation models such as scFoundation and scGPT benefit from large-scale pretraining, and BioLord and PerturbNet integrate broad perturbation-response relationships. As baselines, we used simple statistical predictors (context-mean) and model-based approaches, including linear regression and MLPs with one-hot perturbation encodings (see Materials and Methods).

Using absolute gene expression–level metrics, we observed generally small performance differences between models, with Pearson’s correlation exceeding 0.95 for most datasets (Fig. 2B and fig. S2 to S7). Datasets with smaller perturbation effects tended to exhibit higher correlation-based performance and lower variance across models (e.g., Datlinger_2017 with many low-DE conditions), whereas datasets characterized by larger perturbation effects, such as Replogle and Sunshine, showed lower overall scores (Fig. 2B and figs. S3 to S7). Within datasets, the effect size quantified by Transcriptome-wide Analysis of Differential Expression (TRADE; transcriptome-wide impact) showed a similar trend—smaller-effect perturbations were easier to predict (Fig. 2C and fig. S8). This likely reflects the fact that small-effect perturbations are more difficult to distinguish from technical noise, whereas large-effect perturbations provide a clearer and more reproducible signal for the models to capture.

When moving to an analysis based on expression changes relative to the control, the relationship was reversed. Delta-based Pearson correlation and delta direction accuracy were strongly positively correlated, and perturbations with larger effect sizes yielded better performance (Fig. 2D and fig. S9).

Foundation models exhibited variance compression after fine-tuning, reducing the spread of predicted deltas toward a dataset-wide mean, as confirmed by higher MMD and Wasserstein distances relative to other model classes. Notably, scGPT converges to the population average and has worse distribution alignment as the training epoch increases (Fig. 2E and fig. S10A). This “variance compression” likely arises because the distribution preservation is not in the fine-tuning objectives in the foundation model.

Evaluation of DEG overlap accuracy and effect size ranking further supported the delta-based trends: Datasets with larger perturbation effects achieved higher DE overlap accuracy and stronger concordance in effect size ranking. In this setting, PerturbNet emerged as the clear top performer, achieving a mean overlap accuracy of 0.56, whereas most other methods failed to recover DEGs reliably in several datasets (Fig. 2F and fig. S6, B and C).

Variance decomposition showed that dataset and perturbation identities were primary factors for absolute and delta metrics, while DE accuracy was strongly driven by model choice, dominated by PerturbNet (Fig. 2G). To further improve interpretability, we conducted a granular variance decomposition by explicitly modeling biologically relevant attributes (e.g., KO efficiency and effect size) and technical model design factors (e.g., latent structure and loss objective). This analysis confirmed that dataset-level attributes—particularly perturbation efficiency—mainly explain variance in numerical accuracy, whereas model design choices (in particular, latent space architecture) predominantly govern biological plausibility (DE accuracy) and distributional alignment (fig. S10C).

### Perturbation interaction prediction

**Fig. 3.**
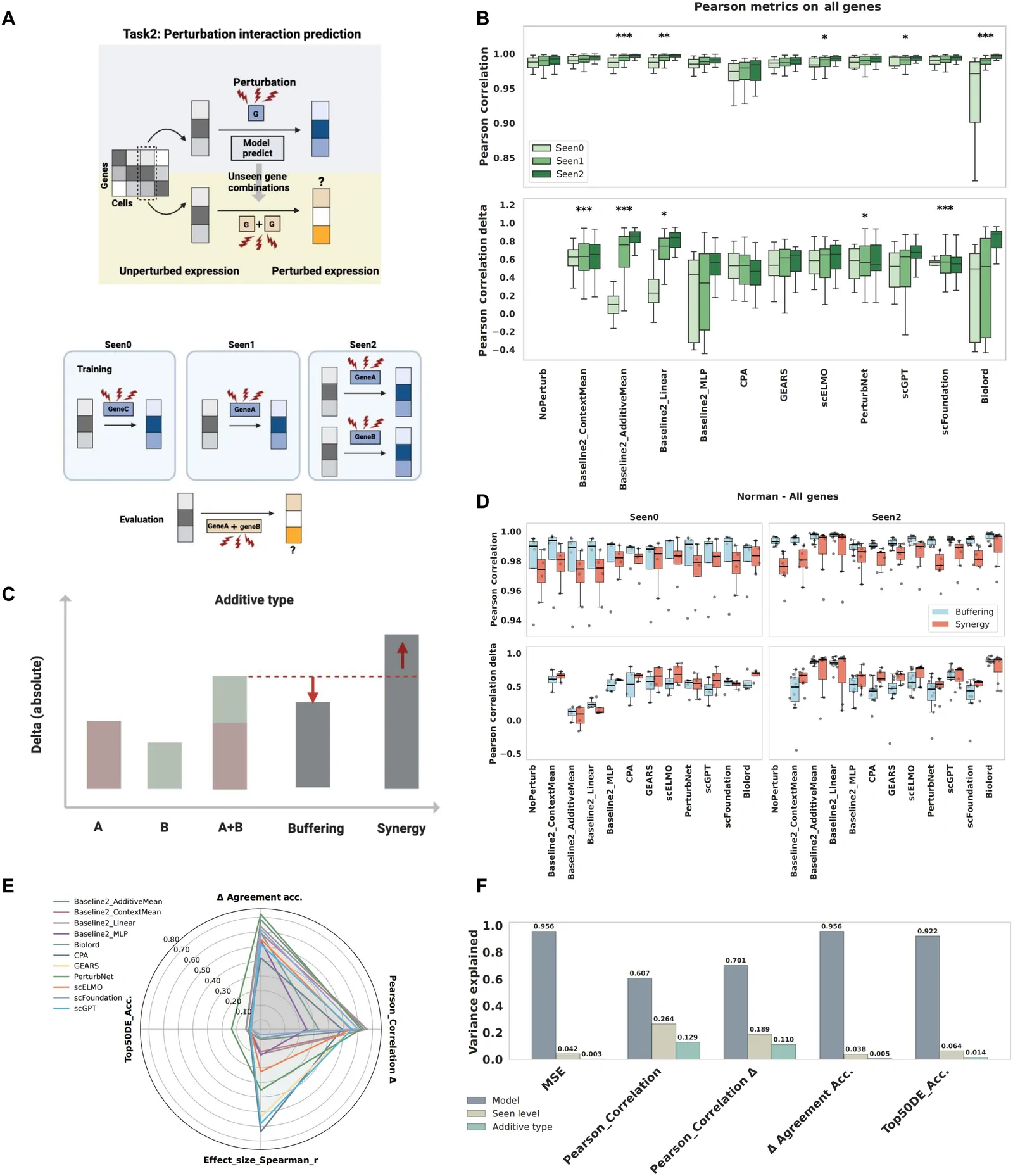
Perturbation interaction prediction performance across models and interaction types. (**A**) Schematic overview of the perturbation interaction prediction task. The bottom panel illustrates the three “seen levels” for combinatorial perturbation evaluation: seen0 (neither individual perturbation in training data), seen1 (one perturbation in training data), and seen2 (both perturbations in training data). Created in BioRender. L. Tian (2026), https://biorender.com/bjpu41h. (**B**) Performance metrics for Pearson’s correlation coefficients on expressions and delta expressions across models comparing seen0, seen1, and seen2 conditions. Significance is shown. (**C**) Classification of combinatorial perturbations into buffering (combination delta smaller than additive expectation) and synergistic (combination delta larger than additive expectation) interaction types. (**D**) Comparison of Pearson correlation and Pearson correlation on deltas between buffering and synergistic combinations under seen0 and seen2 conditions across models, evaluated on all genes in the Norman dataset. (**E**) Averaged top 50 DEG overlap accuracy, effect size correlation (Spearman *r*), Pearson’s correlation coefficients on delta, and delta direction accuracy across all tested models for perturbation interaction prediction. (**F**) ANOVA variance decomposition analysis results showing variance contributions from model choice and interaction type factors for different performance metrics.

Task 2 evaluated each model’s ability to predict gene expression responses to combinatorial perturbations. We considered three “seen levels” on the basis of whether the individual perturbations comprising a combination had appeared in the training data: seen0 (neither perturbation seen), seen1 (one perturbation seen), and seen2 (both perturbations seen) (Fig. 3A) (*14*). Predicting seen0 combinations is the most challenging, as it requires both generalizations to previously unseen perturbations—analogous to Task 1—and inference of interaction effects, whereas seen2 primarily tests a model’s ability to capture the interaction effect of combinatorial perturbation. Models included are CPA, GEARS, scELMo, scFoundation, scGPT, PerturbNet, BioLord, and Baseline models (see Materials and Methods).

Across absolute gene expression–level metrics, most models showed a consistent and significant performance increase from seen0 to seen2, indicating that prior exposure to constituent perturbations in training data improves predictive accuracy. A similar trend was observed for delta Pearson correlations in some methods (Fig. 3B and figs. S11 to S16). Besides, simple baselines proved highly competitive with complex deep learning models, which is consistent with a previous study (*25*). In particular, additive mean and linear models demonstrated superior performance in seen1 and seen2 scenarios, often surpassing more complex architectures in these settings where constituent perturbations were known (Fig. 3B and figs. S11 to S16).

We further classified each combination on the basis of its deviation from the additive expectation—comparing the observed delta for the combination to the sum of deltas for its components—into buffering (combination delta smaller than additive) and synergistic (combination delta larger than additive) categories (Fig. 3C). Across both seen0 and seen2 scenarios, we observed a distinct metric-dependent performance inversion. Buffering combinations consistently achieved higher accuracy in absolute profile correlation (PCC) compared to synergistic ones. In contrast, for delta metrics, synergistic combinations outperformed buffering combinations (Fig. 3D and figs. S17 to S19).

At the delta agreement accuracy and DE evaluation levels, PerturbNet again consistently outperformed all other models, replicating its dominance in Task 1, particularly in DE overlap accuracy, where it achieved substantially higher values, while most other methods struggled to identify any significant DEGs (Fig. 3E and fig. S13D).

Analysis of variance (ANOVA) decomposition revealed that model choice was the predominant determinant across nearly all metrics, overshadowing the contributions of seen levels and additive interaction types. In particular, variance in MSE and MMD was driven primarily by model design, marking a distinct shift from data-driven limitations. Similarly, the accuracy of downstream network retrieval (top 50 DE accuracy) was consistently governed by these design factors (Fig. 3F and fig. S13E).

### Cell state transfer

**Fig. 4.**
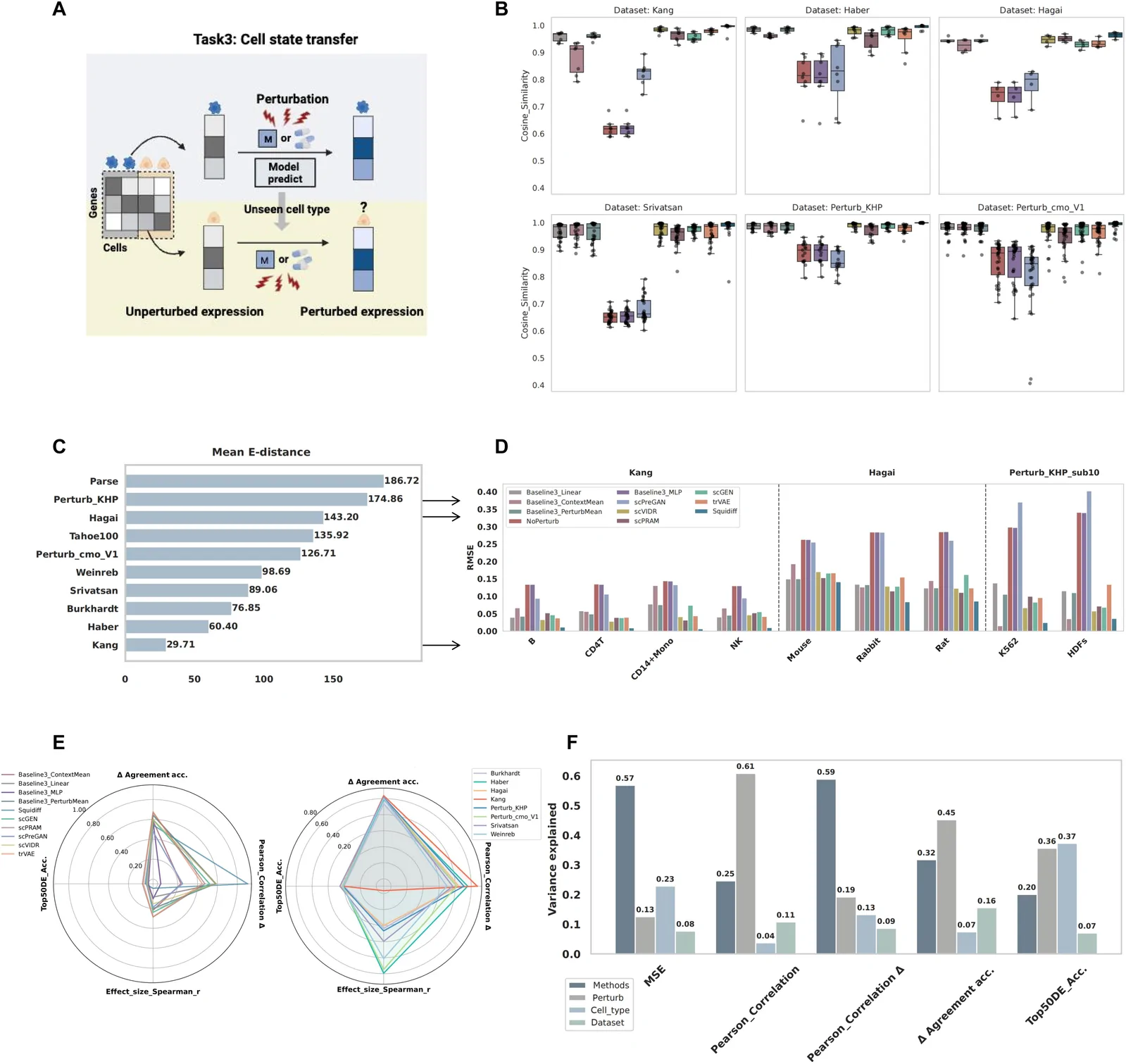
Cell state transfer performance evaluation across cell types. (**A**) Schematic overview of the cell state transfer task framework. Created in BioRender. L. Tian (2026), https://biorender.com/bjpu41h. (**B**) Absolute gene expression–level performance comparison across models and datasets. Cosine similarity is the evaluation metric. (**C**) The average E-distance among cell types for each dataset, ranked by values. (**D**) Model performance in predicting the responses of different cell types, evaluated using RMSE on the selected Kang, Hagai, and Perturb_KHP_sub10 datasets. (**E**) Averaged top 50 DEG overlap accuracy, effect size correlation (Spearman *r*), Pearson’s correlation coefficients on delta expressions, and delta direction accuracy across all tested models for cell state transfer prediction. (**F**) Variance partitioning analysis showing the relative contributions of model choice, perturbation identity, and cell type to the overall transfer performance variability.

Task 3 assessed each model’s ability to generalize known perturbation responses across distinct cell types (Fig. 4A). We evaluated six methods, including scPreGAN, scGen, trVAE, scVIDR, scPRAM, and Squidiff, using diverse datasets in which at least two cell types shared a set of perturbations. A range of baseline methods was used for comparison (see Materials and Methods).

For absolute gene expression–level evaluation, most models (except scPreGAN) achieved similarly high performance across datasets (Fig. 4B and figs. S20 to S24). However, notable variation was observed across datasets: In general, datasets with smaller mean E-distances (E-distance, measuring the separability of perturbed versus control distributions in the high-dimensional space, reflects the perturbation effect size and aligns with signal-to-noise ratios) between source and target cell types yielded better transfer performance (Fig. 4, C and D, and figs. S25 and S26). Smaller E-distances generally correlated with better transfer accuracy [e.g., transfer interferon-β (IFN-β) stimulation in human immune subsets compared with transfer various drug effects from epithelial to K562 cells].

However, E-distance is not fully predictive. For example, results from Burkhardt showed low transfer accuracy despite a small E-distance, likely due to heterogeneous drug effects and high within-group variability, while results from Hagai, with a large domain shift and strong transfer, benefited from conserved immune activation signatures. These results indicate that Task 3 performance is shaped not only by baseline domain differences between cell types but also by the consistency of perturbation effects across cell types (figs. S25 and S26).

Delta-level performance mirrored absolute-level trends, with trVAE leading in direction accuracy, followed by scPRAM, and Squidiff performing best in delta Pearson correlation, followed by scVIDR (Fig. 4E). At the DE level, all models performed poorly, consistent with findings from previous tasks (Tasks 1 and 2), indicating a persistent challenge in accurately predicting DEGs (Fig. 4E).

Variance decomposition quantified a functional decoupling between numerical reconstruction and biological retrieval in cross-cell-type transfer tasks. Global alignment metrics (MSE and delta Pearson) were predominantly determined by algorithmic design factors, specifically loss formulation and latent space structure. Conversely, the accuracy of DEG identification (top 50 DE accuracy) was governed by target cell identity (Fig. 4F and fig. S26B).

## DISCUSSION

This study benchmarks single-cell perturbation response prediction across three complementary settings: unseen single perturbations, combinatorial perturbations, and cross-cell-state transfer. Across these tasks, a consistent pattern emerged. Many models achieved high accuracy when evaluated by absolute gene-expression reconstruction, yet this apparent success did not consistently translate into the accurate recovery of perturbation-induced changes, DEGs, distributional similarity, or transfer across cellular contexts. Thus, the central practical message from our benchmark is that perturbation prediction performance cannot be summarized by a single global score; each metric must be interpreted in terms of the biological signal it is able to measure.

Perturbation effect size was a major determinant of this discrepancy. For weak perturbations, absolute expression metrics such as correlation or MSE often yielded high scores because perturbed profiles remained close to control states and most genes were unchanged. In this regime, raw profile metrics have limited discriminative power: A model can appear accurate by reconstructing the basal expression state while failing to recover the perturbation-specific response. In contrast, delta-based metrics emphasize expression changes relative to control and become more informative when perturbation effects are large enough to rise above technical and sampling noise. DEG recovery imposes an even stricter requirement, because models must predict not only the correct direction of change but also sufficiently calibrated effect sizes and dispersion to support statistical detection. These observations are consistent with recent work showing that standard perturbation metrics can overestimate biological prediction accuracy and that metric design remains a critical challenge for virtual-cell benchmarking (*26*, *30*, *31*).

**Fig. 5.**
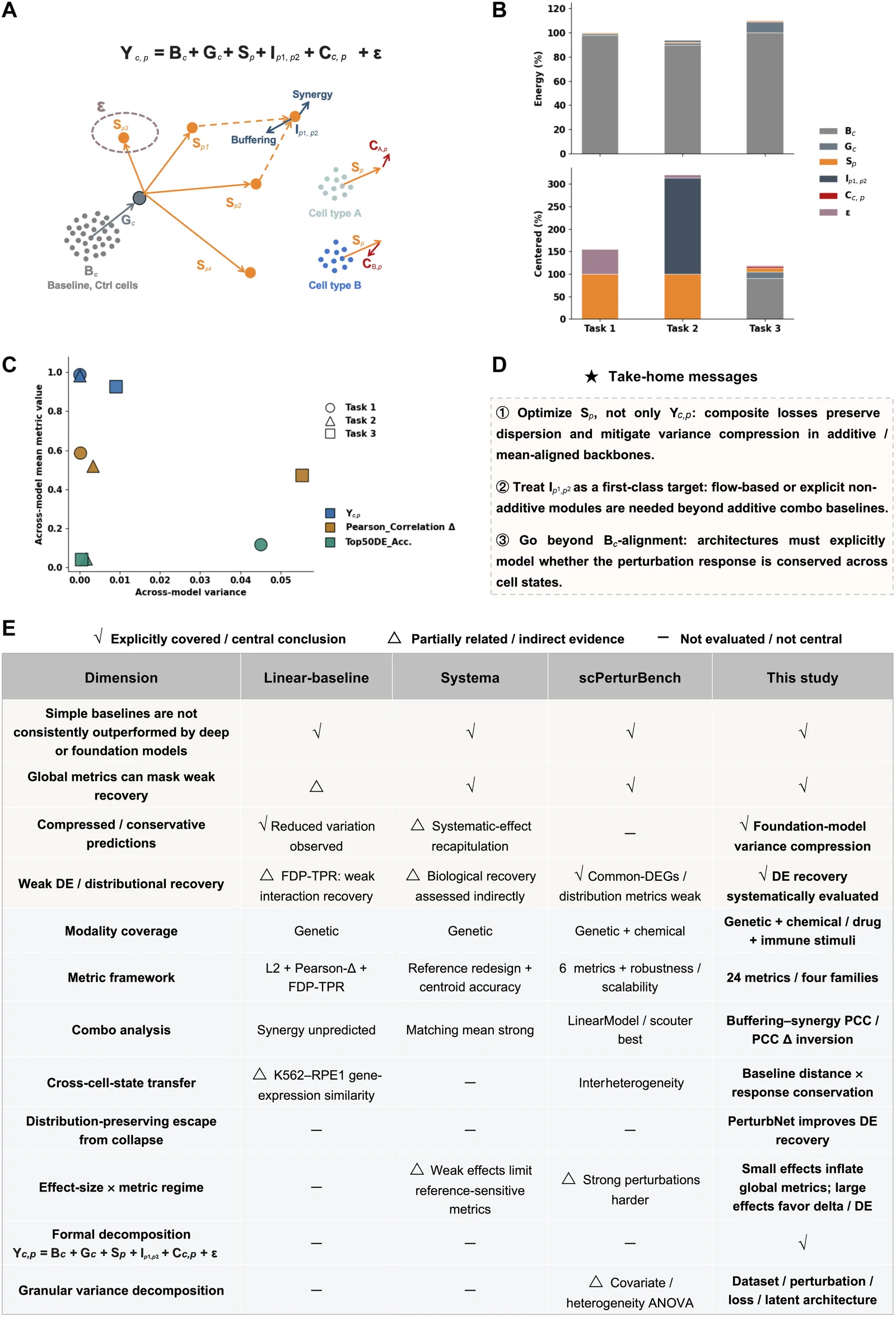
Decomposition-based interpretation of perturbation-prediction benchmarks. (**A**) Schematic of the proposed decomposition $Y_{c,p}=B_{c}+G_{c}+S_{p}+I_{p_{1},p_{2}}+C_{c,p}+\varepsilon$, separating baseline cell-state expression, systematic shifts, perturbation-specific effects, combinatorial interactions, cell state–specific residuals of the perturbation response, and residual noise. (**B**) Component-energy decomposition across the three benchmark tasks. The upper panel shows energy fractions normalized by total expression energy, and the lower panel shows the corresponding centered decomposition after subtracting the gene-wise grand mean across evaluated entries. Fractions are component-wise normalized sum-of-squares values and are not constrained to sum to 100% because the terms do not form a strictly orthogonal variance partition. (**C**) Across-model mean performance versus across-model variance for three representative evaluation readouts: full-profile $Y_{c,p}$, Pearson correlation Δ, and top 50 DEG overlap accuracy. Marker shape denotes task, and color denotes readout. For each task-readout pair, values were calculated across retained prediction models after excluding NoPerturb and Baseline-related models. (**D**) Take-home messages. (**E**) Summary comparison of this study with three recent perturbation-prediction benchmarks: the linear-baseline benchmark (*25*), the Systema framework (*26*), and the scPerturBench generalizability benchmark (*31*).

To formalize why different metrics diverge and why model rankings shift across evaluation families, we introduced a signal-decomposition framework (Fig. 5). We represent a perturbed expression profile as containing several evaluable components: the basal cellular state of the context ($B_{c}$), perturbation-specific effects ($S_{p}$), nonadditive interaction residuals in combinatorial perturbations ($I_{p_{1},p_{2}}$), context-dependent modulation of perturbation responses ($C_{c,p}$), and residual noise or unexplained variation (ϵ). This decomposition is intended as a conceptual evaluative scaffold, not as a mechanistic linear model of gene regulation. To quantify how much variance each component contributes to a given metric, we used linear model fitting as a statistical tool. The analysis showed that raw expression-level metrics are dominated by $B_{c}$, whereas centered, delta-based, DE-based, and distributional metrics expose additional contributions from perturbation-specific, interaction-specific, and context-dependent components. This explains why model rankings can change substantially across metric families: Different metrics are not redundant summaries of the same prediction problem but probes of different biological signals.

This decomposition clarifies why high reconstruction accuracy and poor DEG recovery can coexist. Correct DE recovery requires calibrated mean shifts and cell-to-cell variability. A model that produces overly smooth predictions may preserve global profile similarity while distorting the dispersion structure needed for reliable DE testing. In our benchmark, this issue was particularly evident for reconstruction-oriented autoencoder (AE)/VAE–based models and fine-tuned foundation models, which often aligned broad expression profiles but compressed response heterogeneity. In contrast, distribution-aware generative approaches are better aligned with the single-cell nature of the task because they aim to predict a population of perturbed cells rather than only a single average profile. Consistent with this view, PerturbNet showed improved DEG recovery in our benchmark (*32*). However, our conclusion is not that one architecture universally solves perturbation prediction. Rather, models should be evaluated for distributional calibration, variance preservation, and DE validity in addition to mean-response accuracy.

For combinatorial perturbations, the signal-decomposition view reframes the task from predicting the full double-perturbation profile to recovering the nonadditive residual beyond single-perturbation superposition. The discrepancy between buffering and synergistic combinations under different metrics should therefore be interpreted through $I_{p_{1},p_{2}}$ rather than through raw profile similarity alone. Buffering combinations can appear favorable under absolute profile metrics because their responses remain closer to additive or control-like expectations, whereas synergistic combinations are more visible under delta-based metrics because of larger response magnitude. Our effect size–controlled analysis further shows that combinatorial benchmarks must separate response magnitude from interaction class before attributing performance differences to biological nonadditivity. This finding extends recent observations that simple additive or linear baselines remain difficult to outperform in double-perturbation prediction (*25*, *31*).

Cross-cell-state transfer revealed another distinct failure mode. Model performance was not determined by basal-state distance alone. Although large distances between control cell states can make transfer more difficult, our results indicate that transferability depends jointly on basal-state similarity and conservation of the perturbation response program across contexts. This explains why models can transfer across apparently distant contexts when the underlying response program is conserved yet fail between closer contexts when perturbation responses are rewired. In other words, the challenge in Task 3 is not simply statistical alignment between cell types but biological transportability of the perturbation mechanism.

These task-specific diagnoses connect directly to three principles for future model development and evaluation. First, models should be trained and evaluated against perturbation-specific effects ($S_{p}$) rather than raw profiles alone. Absolute expression reconstruction remains useful as a sanity check, but it should be accompanied by effect size–stratified, centered, delta-based, DE-based, and distributional metrics. Second, combinatorial prediction should explicitly target nonadditive interaction residuals ($I_{p_{1},p_{2}}$) beyond additive single-perturbation expectations. This will require architectures or objectives that can represent a nonlinear interaction structure rather than defaulting to additive superposition. Third, cross-context prediction should move beyond basal-state alignment and explicitly model whether perturbation response programs are conserved or rewired across cellular states ($C_{c,p}$). To facilitate the practical application of our findings, we provide a decision framework to guide method selection on the basis of experimental objectives and dataset characteristics (fig. S27).

Several limitations of the present benchmark also point toward concrete experimental design requirements. The datasets included in this benchmark were generated using different technologies, perturbation modalities, cell types, and experimental protocols. These differences introduce batch effects and data-quality variation that can influence both model training and evaluation. Our variance analysis indicates that intrinsic dataset properties can contribute as much to performance variation as model choice, emphasizing the need for standardized perturbation datasets with matched controls, sufficient replicates, and carefully measured perturbation efficiency. In addition, current single-cell sequencing is destructive, so we cannot observe the same cell before and after perturbation. As a result, all models infer population-level counterfactuals rather than true paired single-cell trajectories. The lack of dense temporal sampling further limits our ability to distinguish direct perturbation effects from downstream adaptation or state transitions.

Future perturbation benchmarks should therefore be designed around the unresolved signal components identified here. Multi–time point data would help separate immediate perturbation responses from later regulatory adaptation. Multimodal measurements, including chromatin accessibility, protein abundance, genomic variation, and molecular perturbation representations, could identify mechanisms invisible from transcriptomes alone (*33*, *34*). Spatial perturbation technologies may extend virtual-cell modeling from isolated cells to tissue-level response prediction (*35*). Critically, future cross-context benchmarks should deliberately sample four regimes: similar basal states with conserved responses, distant basal states with conserved responses, similar basal states with divergent responses, and distant basal states with divergent responses. Such designs would distinguish easy transfer settings from hidden context-specific failures and genuinely hard out-of-distribution regimes. These data types should not be added merely for scale but to improve the recovery of perturbation-specific effects, nonadditive interactions, and context-dependent response programs. In this sense, the path toward virtual-cell models is not simply to train larger foundation models but to build perturbation-aware systems that combine robust cellular state representations, causal perturbation data, distributional response modeling, and experimentally grounded evaluation (*36*).

In summary, our benchmark shows that current perturbation predictors often succeed on basal-state reconstruction but remain limited on the biologically decisive components of the task: perturbation-specific effects, nonadditive interaction residuals, calibrated response distributions, and context-dependent response conservation. By decomposing prediction performance into these components, our study provides a framework for interpreting existing benchmarks and designing future models, metrics, and experiments for single-cell perturbation response prediction.

## MATERIALS AND METHODS

### Ethics statement

Human dermal fibroblasts (HDFs) and peripheral blood mononuclear cells (PBMCs) were collected from two male donors with informed consent. The use of both HDFs and PBMCs in this study was approved by the Westlake University Ethics Committee (approval number: 20240909LXD001).

### Cell culture and drug treatment

#### Perturb_KHP dataset

HDFs were cultured in Dulbecco’s modified Eagle’s medium (C11965500BT, Gibco) supplemented with 10% fetal bovine serum (FBS; C04002, VivaCell), 1% nonessential amino acids (11140-050, Gibco), 1 mM GlutaMAX (35050-061, Gibco), 55 μM 2-mercaptoethanol (21985-023, Gibco), 1 mM sodium pyruvate (11360-070, Gibco), and 1% penicillin-streptomycin (15140-122, Gibco). PBMCs were cultured in StemPro-34 SFM Medium (10639-011, Gibco) supplemented with StemPro-34 Nutrient Supplement, 1 mM GlutaMAX (35050-061, Gibco), stem cell factor (100 ng/ml), FLT-3 (FMS-related tyrosine kinase 3 ligand; 100 ng/ml), IL-3 (interleukin-3; 20 ng/ml), and IL-6 (20 ng/ml). K562 cells, obtained from American Type Culture Collection, were cultured in RPMI 1640 medium (61870-036, Gibco) with 10% FBS and 1% penicillin-streptomycin.

HDFs from two donors were seeded into 12-well plates at a density of 1 × 10^5^ cells per well. PBMCs from the same donors were thawed and seeded into 96-well low-attachment plates at a density of 4 × 10^5^ cells per well. K562 cells were seeded at a density of 1 × 10^5^ cells per well in either 96-well or 12-well plates. K562 cells were rested for 24 hours, while HDFs and PBMCs were rested for 48 hours before drugs were added to each well to reach the specified final concentrations. Half of the medium was refreshed daily. A detailed list of drugs and concentrations used for each cell type is provided in table S3. K562 cells were collected for scRNA-seq after 3 days of treatment, while HDFs and PBMCs were collected after 4 days.

#### Perturb_cmo_V1 dataset

Frozen human PBMCs from three healthy donors (aged 20 to 25 years; Stem Cell Technologies, no. 70025.1) were thawed and cultured in 96-well low-attachment plates at a seeding density of 4.5 × 10^5^ cells per well. Cells from two vials originating from two donors were mixed before seeding. PBMCs were maintained in RPMI 1640 medium (61870-036, Gibco) supplemented with 10% heat-inactivated FBS (C04002, VivaCell) and 1% penicillin-streptomycin (15140-122, Gibco). Cultures were allowed to acclimate for 24 hours before drug treatment, with half of the medium replaced daily to sustain cell viability. After 4 days of continuous treatment, cells were harvested for downstream scRNA-seq.

A total of 111 drugs from the Drug Repurposing Compound Custom Library (L9200, TargetMOI) were resuspended at 10 mM in DMSO (dimethyl sulfoxide) and stored at −80°C. For PBMC treatment, drugs were diluted in culture medium to a final concentration of 1 μM. Control groups received DMSO at an equivalent dilution. A detailed list of drugs used is provided in table S4.

### Sample multiplexing for scRNA-seq

Sample multiplexing was conducted using the 3′ CellPlex Kit Set A (10x Genomics, 1000261), following the manufacturer’s instructions. Briefly, cells from each well were collected and labeled with unique cell multiplexing oligonucleotides. For the Perturb_KHP dataset, equal numbers of live cells from drug-treated and untreated groups were pooled into a single sample. For HDFs and PBMCs, cells from two donors treated with the same drug were mixed before incubating with the multiplexing oligos. A total of two pooled samples were prepared for K562 cells, two for HDFs, and two for PBMCs. For the Perturb_cmo_V1 dataset, cells were pooled at equal viability-adjusted numbers across eight treatment groups per sample, generating a total of 16 pooled samples. All samples underwent library preparation using the 10x Chromium Next GEM Single Cell 3′ Kit version 3.1 (10x Genomics, 1000123).

### Datasets and preprocessing

According to the research conducted by Peidli *et al.* (*8*), we acquired the Datlinger (*4*, *37*), Dixit (*3*), Frangieh (*38*), Papalexi (*39*), Replogle (*40*), Tian (*41*), and Srivatsan (*42*) datasets from Zenodo (access link: https://zenodo.org/records/7041849). For the Datlinger and Frangieh datasets, we curated the perturbation_2 subset, excluding perturbations related to “stimulate” and “IFN-γ,” as well as “Co-culture,” while retaining only the CRISPR-Cas9 perturbations. The Sunshine (*43*) dataset was obtained from the NCBI Gene Expression Omnibus (GEO) under accession number GSE208240, where we filtered for entries classified as “exact_match,” “no_match,” or “scrambled_pair” and excluded entries that contained nontargeting sequences in the “guide_identity.”

We further expanded our dataset collection by including the Joung and Xu datasets directly from the authors, the Virtual Cell Challenge train dataset from https://virtualcellchallenge.org/app/datasets, the Maya M. Arce dataset from GSE278572, the Tahoe dataset from https://github.com/ArcInstitute/arc-virtual-cell-atlas/tree/main, the Parse Biosciences dataset from https://parse-wget.s3.us-west-2.amazonaws.com/10m/Parse_10M_PBMC_cytokines.h5ad, and the Burkhardt dataset from www.kaggle.com/competitions/open-problems-single-cell-perturbations/data. Because of the large size of the Tahoe dataset, which consists of 14 plates, we implemented a specialized preprocessing pipeline. For each plate, we first retained combinations of cell name and drug with at least 1000 cells and then randomly sampled up to 100 cells per group. Subsequently, we filtered conditions to retain only those with at least 40 unique cell types within each plate while preserving control conditions. The 14 processed plates were then merged into a single integrated dataset. For the Srivatsan, Burkhardt, Tahoe, and Parse Biosciences datasets, as well as our two in-house datasets, we used TRADE (TRADEtools version 0.99.0) to compute transcriptome-wide impact scores for each perturbation condition. On the basis of these scores, we selected the top five and bottom five ranked conditions for retention (detailed in table S5). The implementation details of TRADE are provided below.

Data normalization and log transformation were performed using the scanpy (version 1.10.1) toolkit. For datasets involving gene perturbations, we implemented a sample filtering process to ensure adequate coverage and minimize noise. In particular, we applied stratified subsampling to groups with large sample sizes, limiting the maximum sample count per group to 3000. Groups with fewer than 50 samples were excluded to reduce potential errors arising from small sample sizes. To enhance the robustness of feature selection, we calculated and retained the top 5000 highly variable genes. Within datasets containing gene perturbations, we identified perturbed genes on the basis of the “condition” field and extracted their names, thereby creating a subset of genes directly influenced by the experimental conditions. Ultimately, we combined the identified perturbed genes with the highly variable genes to generate the final gene set used for data filtering, ensuring the preservation of the most informative genes associated with perturbations. For datasets not involving gene perturbations, we retained only the top 5000 highly variable genes for filtering. However, for the Xu dataset, we extended the feature selection to the top 20,000 highly variable genes, as restricting to 5000 genes resulted in insufficient perturbation conditions for analysis.

In addition, we downloaded the Norman (*44*) and Adamson (*45*) datasets from https://dataverse.harvard.edu/api/access/datafile/6154020 and https://dataverse.harvard.edu/api/access/datafile/6154417, along with the Kang (*46*), Haber (*47*), and Hagai (*48*) datasets from the scGen study (https://github.com/theislab/scgen-reproducibility/blob/master/code/DataDownloader.py). The Weinreb (*49*) dataset was sourced from the CellOT study (www.research-collection.ethz.ch/handle/20.500.11850/609681) (*50*). Given that these datasets were appropriately preprocessed in their original studies, no further data processing steps were applied. All data analysis was performed on an in-house Linux server with Ubuntu operating system (version 22.04) and the Data Science Platform of Guangzhou National Laboratory (*51*).

The DEGs are calculated using the rank_genes_groups function in scanpy with method=‘wilcoxon.’ For each perturbation condition, cells from both the perturbation and control (ctrl) groups are extracted to form a subset using normalized expression data. After obtaining the DE results, genes are sorted by the absolute values of their scores in descending order to prioritize genes with the strongest DE regardless of up- or down-regulation direction. The top 20, 50, and 100 DEGs based on absolute scores are then selected and used to subset the gene expression matrix for calculating the evaluation metrics.

### Model selection and setup

In this study, we selected and applied a range of advanced modeling approaches to systematically predict and compare single-cell perturbation responses. The models span diverse theoretical frameworks and algorithmic structures, ranging from baseline models to foundation models, GRN–based, VAE-based, and other deep learning–based approaches. These approaches offer strong adaptability and generalization capabilities, allowing effective application across diverse perturbation contexts through training or fine-tuning.

#### Baseline model

In single-cell perturbation prediction tasks, baseline models provide reference performance levels that facilitate the evaluation of more expressive predictive models (*28*, *52*–*54*). Accordingly, we design task-specific baseline methods for each task, including MLP-based baselines, simple statistical predictors, and linear regression models.

##### Baseline1_MLP

The baseline uses one-hot encoding to represent the cell’s gene expression under perturbation *A* as $p_{A}$. The one-hot encoding of perturbations *A* is embedded into $z_{A}$. The gene expression of the cell under the control (no perturbation) condition is embedded into $z_{\text{ctrl}}$. The gene expression after combined perturbations can be represented as $x^{\prime }=\text{MLP}\left(z_{A}+z_{\text{ctrl}}\right)$.

##### Baseline1_ContextMean

This baseline predicts the average gene expression across all training perturbations for the given cell type. Let $P_{\text{train}}$ denote the set of training perturbations. The prediction is defined as

$$\hat{x}=\frac{1}{|P_{\text{train}}|}\sum_{p\in P_{\text{train}}}E\left[X_{p}\right]$$ (1)

##### Baseline1_Linear

This baseline uses a multioutput linear regression model that maps one-hot–encoded perturbations to gene expression profiles

$$\hat{x}=Wp+b$$ (2)

where **p** denotes the perturbation encoding, and *W* and **b** are learned parameters.

##### Baseline2_MLP

We still use one-hot encoding to represent the gene expression of the cell under perturbation *A* as $p_{A}$ and perturbation *B* as $p_{B}$. In addition, the one-hot encoding of perturbations *A* and *B* is embedded into $z_{A}$ and $z_{B}$, respectively. The gene expression after combined perturbations can be represented as $x^{\prime }=\text{MLP}\left(z_{A}+z_{B}+z_{\text{ctrl}}\right)$.

##### Baseline2_ContextMean

This baseline is defined analogously to Task 1 and predicts the average gene expression across all training perturbations for the given cell type.

##### Baseline2_AdditiveMean

This perturbation-mean baseline assumes additive effects of individual perturbations. For a combination of perturbations *A* and *B*, the prediction is

$${\hat{x}}_{A+B}=x_{\text{ctrl}}+\Delta_{A}+\Delta_{B}$$ (3)

where

$$\Delta_{A}=E\left[X_{A+\text{ctrl}}\right]-x_{\text{ctrl}}$$ (4)

$$\Delta_{B}=E\left[X_{B+\text{ctrl}}\right]-x_{\text{ctrl}}$$ (5)

##### Baseline2_Linear

This baseline uses a multioutput linear regression model with one-hot encoding that jointly indicates both perturbed genes

$$\hat{x}=Wp_{A+B}+b$$ (6)

##### Baseline3_MLP

We train the model for other cells under a single perturbation *A*, which is defined as $F_{A}\left(z_{\text{other}\_\text{ctrl}}+p_{A}\right)$, where $z_{\text{other}\_\text{ctrl}}$ is the embedding of the gene expression of other cells under the control (no perturbation) condition, and $p_{A}$ is the one-hot encoding of perturbation *A*. For the gene expression of the target cell under perturbation *A*, it can be represented as $F_{A}\left(z_{\text{target}\_\text{ctrl}}+p_{A}\right)$, where $z_{\text{target}\_\text{ctrl}}$ is the embedding of the gene expression of the target cell under the control condition.

##### Baseline3_ContextMean

This baseline predicts the average expression of the target cell type across all perturbations observed for that cell type in the training data

$${\hat{x}}_{c}=\frac{1}{|P_{\text{available}}|}\sum_{p\in P_{\text{available}}}E\left[X_{c,p}\right]$$ (7)

##### Baseline3_PerturbMean

This baseline predicts the response of the target cell type by adding the average perturbation effect, estimated from other cell types, to the target cell type’s control expression

$${\hat{x}}_{c_{\text{target}},p}=x_{c_{\text{target}},\text{ctrl}}+\Delta_{p}$$ (8)

where

$$\Delta_{p}=\frac{1}{|C_{\text{other}}|}\sum_{c\in C_{\text{other}}}\left(E\left[X_{c,p}\right]-E\left[X_{c,\text{ctrl}}\right]\right)$$ (9)

##### Baseline3_Linear

This baseline uses a multioutput linear regression model that jointly encodes cell type and perturbation information via concatenated one-hot vectors.

#### Foundation model–based

The pretrained models scGPT (version 1.0) and scFoundation (version 1.0) were used for the perturbation prediction task, with fine-tuning performed on the basis of the strategy used in the scGPT tutorial. In their approach, a Binary Condition Token was appended at each input gene position to denote the perturbation status of the gene. Besides, we implemented a strategy wherein a nonperturbed control cell was used as the input and a perturbed cell as the target. This was achieved by randomly matching each perturbed cell with a control cell, thereby forming input-target pairs. The models were trained using the default settings recommended in the tutorials, with scGPT undergoing 2 or 5 epochs of training for comparison and scFoundation trained for 2 epochs.

#### GRN-based

GEARS (version 1.0) was used to model gene perturbations by seamlessly integrating knowledge graph information with gene embeddings. We followed the tutorial guidelines using the default settings as recommended. For the scELMo (version 1.0) model, we followed the tutorial’s instructions and incorporated gene embeddings derived from GPT as inputs for gene embedding in GEARS.

#### VAE-based

The VAE-based approaches, PerturbNet (version 0.0.3) (*32*), scGen (version 2.1.0) (*55*), trVAE (version 1.0.0) (*56*), scVIDR (version 1.0) (*57*), and scPRAM (0.0.2) (*58*), were used for scRNA-seq perturbation prediction. PerturbNet combines a convolutional neural network with a VAE to capture complex patterns in single-cell gene expression data. The model learns a latent representation of gene expression and leverages this to predict perturbation effects across different conditions. We implemented PerturbNet according to the author’s instructions using the default architecture and hyperparameters. Note that PerturbNet requires chemical structure information (SMILES representations) as input and is therefore only applicable to drug perturbation datasets. To ensure a fair comparison, main task figures present only models evaluated on shared datasets and conditions; PerturbNet’s performance on drug-specific datasets in Task 3 is presented in the Supplementary Materials.

scGen leverages a VAE framework integrated with vector arithmetic to simulate cellular transitions between conditions, such as control and stimulation. The model encodes gene expression profiles into a latent space, reconstructing them via a decoder while learning different vectors between cellular states. This enables scGen to predict unobserved perturbation responses with high generalization. We implemented scGen following the tutorial provided by the authors, adhering to the recommended architecture and hyperparameters.

trVAE builds on the conditional VAE framework to model perturbation effects under scenarios lacking paired control and perturbed cells. The model encodes cellular states into a shared latent space while applying condition-specific transformations via perturbation vectors. This is optimized using a masked learning objective and MMD regularization to align distributions across conditions. The implementation followed the author’s tutorial, with early stopping enabled and the maximum iterations set to 1000, while all other hyperparameters were kept as default.

scVIDR extends the VAE-based perturbation prediction framework by addressing cell type–specific heterogeneity in dose-response relationships. The model uses a VAE to encode single-cell gene expression data into a latent space and then uses linear regression to predict cell type–specific perturbation vectors ($\delta_{c}$) on the basis of control group latent representations. For dose-response prediction, scVIDR performs log-linear interpolation on the estimated perturbation vectors to predict intermediate doses, followed by decoding back to the gene expression space. The model introduces a “pseudo-dose” metric to characterize individual cell sensitivity to chemical perturbations by projecting cells onto the perturbation axis in a latent space. We implemented scVIDR following the authors’ specifications, with default hyperparameters.

scPRAM uses an attention mechanism–based approach for predicting single-cell perturbation responses while accounting for cellular heterogeneity. The model consists of three main components: a VAE for encoding high-dimensional gene expression data into a latent space, optimal transport based on the Sinkhorn algorithm to match unpaired cells before and after perturbation, and an attention mechanism to compute perturbation vectors for test cells. The attention mechanism treats the test cell’s latent representation as a query using cosine similarity to identify the most relevant training cells and then computes weighted perturbation vectors through a softmax-normalized attention score. This approach enables scPRAM to capture individual cell–specific responses rather than relying on population averages. We implemented scPRAM with default parameters.

#### Other deep learning–based

CPA (version 0.8.7) (*59*) combines deep learning flexibility with linear model interpretability to predict cellular responses to perturbations. The model uses an encoder to map gene expression data into a disentangled latent space, removing perturbation-specific information via adversarial training. The latent embedding is then combined with perturbation and covariate embeddings, scaled by a learned dose-response function for cell-specific predictions. The decoder reconstructs gene expression profiles, enabling predictions for unseen perturbations. We followed the authors’ tutorial and used the default hyperparameters for implementation.

scPreGAN (version 1.0) (*60*) integrates AEs with generative adversarial networks (GANs) to predict cellular responses to perturbations. The architecture includes an encoder, two generator networks, and two discriminators. The encoder extracts features, while the generators predict perturbed data from latent vectors. Pretraining combines reconstruction and adversarial losses, optimizing data fidelity and distribution realism. We implemented scPreGAN on the basis of the authors’ GitHub tutorial, adhering to default settings and niter set to 1000.

Biolord (version 0.03) (*61*) uses a deep generative framework to disentangle single-cell data into known and unknown attributes through a decomposed latent space. The model separates each known attribute (cell type, time, and perturbation) into dedicated subnetworks while capturing unknown attributes in regularized embeddings. A generator module reconstructs cellular measurements from these disentangled representations. The framework is optimized using a composite loss function that balances reconstruction accuracy with information minimality constraints, enabling counterfactual predictions by modifying specific attributes while preserving cell-specific signatures. We implemented Biolord following the official tutorial with default parameters and max_epochs set to 1000.

Squidiff (version 1.0.8) (*62*) is a conditional diffusion–based generative framework designed to model single-cell transcriptomic dynamics under diverse biological conditions. The model integrates a semantic encoder to map gene expression profiles into a biologically meaningful latent space and a denoising diffusion implicit model to generate transcriptomes. By manipulating semantic latent variables via interpolation and vector arithmetic, Squidiff enables the prediction of cellular responses to perturbations across cell types. The model is trained following the official implementation with default training settings using a diffusion process with 1000 time steps, with input gene expression profiles restricted to the top 200 highly variable genes.

### Evaluation metrics

In evaluating model performance, selecting the appropriate assessment metrics is essential. To provide a comprehensive evaluation, this study uses a standardized, multidimensional evaluation framework that integrates four complementary categories of metrics: absolute accuracy, relative effect capture, DE recovery, and distributional similarity. These metrics offer a holistic view of the model’s predictive accuracy, biological interpretability, and distributional consistency across various perturbation scenarios.

In particular, absolute accuracy is assessed through error-based measures (such as MSE, RMSE, MAE, and *L*_2_ loss), which quantify the deviations between predicted and actual gene expression values; goodness-of-fit metrics (such as *R*^2^), which evaluate the model’s ability to account for overall data variability; and correlation analysis (including Pearson correlation and cosine similarity), which measures the linear relationship and directional alignment between predicted and actual values. Relative effect capture is evaluated by computing Pearson correlation on delta values (expression changes between perturbed and control conditions) to assess the model’s ability to predict perturbation-induced changes, along with delta direction accuracy, which quantifies the proportion of genes with correctly predicted up- or down-regulation patterns. DE recovery is measured through DE overlap accuracy, which assesses the agreement between predicted and observed DEGs, as well as Spearman correlation of effect sizes, which evaluates the consistency of perturbation magnitudes across different conditions. Last, distributional similarity metrics (such as MMD and Wasserstein distance) assess the alignment between predicted and actual gene expression distributions, capturing the nuanced distributional characteristics in high-dimensional data.

#### Absolute accuracy metrics

##### Mean squared error (MSE)

MSE is a commonly used metric for evaluating the model error, quantifying the mean squared difference between predicted and actual values. MSE is particularly suited to assessing prediction bias and overall accuracy, given its sensitivity to larger errors. In calculation, MSE squares the prediction error for each sample and then averages these values. The formula is as follows

$$\text{MSE}=\frac{1}{n}\sum_{i=1}^{n}{\left(y_{i}-{\hat{y}}_{i}\right)}^{2}$$ (10)

where $y_{i}$ is the actual value, ${\hat{y}}_{i}$ is the predicted value, and *n* is the number of samples.

##### Root mean squared error (RMSE)

RMSE is similar to MSE, being the square root of MSE, but its units are consistent with the original data, providing a more intuitive reflection of the magnitude of prediction errors. It is obtained by taking the square root of MSE, defined as follows

$$\text{RMSE}=\sqrt{\frac{1}{n}\sum_{i=1}^{n}{\left(y_{i}-{\hat{y}}_{i}\right)}^{2}}$$ (11)

##### Mean absolute error (MAE)

MAE measures the absolute error between predicted and actual values, is less sensitive to outliers than MSE, and is suitable for scenarios where one does not wish to penalize large errors. MAE was calculated by taking the average of the absolute difference between the predicted and actual values, defined as follows

$$\text{MAE}=\frac{1}{n}\sum_{i=1}^{n}|y_{i}-{\hat{y}}_{i}$$ (12)

##### L_2_ norm (L_2_)

*L*_2_ norm(*L*_2_) is used to measure the Euclidean distance between predicted and actual values, serving as an effective tool for describing the error space. It calculates the *L*_2_ norm of the difference vector between predicted and actual values, defined as follows

$$L_{2}=\sqrt{\sum_{i=1}^{n}{\left(y_{i}-{\hat{y}}_{i}\right)}^{2}}$$ (13)

##### *R* ^2^

$R^{2}$ is used to measure a model’s ability to explain the total variance, serving as an indicator of model fit. It represents the degree of correlation between predicted and actual values and is defined as

$$R^{2}=1-\frac{\sum_{i=1}^{n}{\left(y_{i}-{\hat{y}}_{i}\right)}^{2}}{\sum_{i=1}^{n}{\left(y_{i}-\bar{y}\right)}^{2}}$$ (14)

In this formula, $\bar{y}$ is the mean of the actual values. The numerator, $\sum_{i=1}^{n}{\left(y_{i}-{\hat{y}}_{i}\right)}^{2}$, represents the residual sum of squares, which is the total squared error. The denominator, $\sum_{i=1}^{n}{\left(y_{i}-\bar{y}\right)}^{2}$, represents the total sum of squares of the actual values.

##### Pearson’s correlation

Pearson’s correlation measures the linear relationship between predicted and actual values, making it suitable for analyzing the strength of association between datasets. It is calculated by dividing the covariance between predicted and actual values by the product of their standard deviations, defined as

$$r=\frac{\sum_{i=1}^{n}\left(y_{i}-\bar{y}\right)\left({\hat{y}}_{i}-\bar{\hat{y}}\right)}{\sqrt{\sum_{i=1}^{n}{\left(y_{i}-\bar{y}\right)}^{2}}\sqrt{\sum_{i=1}^{n}{\left({\hat{y}}_{i}-\bar{\hat{y}}\right)}^{2}}}$$ (15)

##### Cosine similarity

Cosine similarity measures the directional similarity between two vectors, making it suitable for evaluating the similarity between model predictions and actual results. The cosine similarity between the predicted and actual value vectors is defined as

$$\begin{array}{c} \text{Cosine similarity}\left(y_{i},{\hat{y}}_{i}\right)=\\ \frac{y_{i}\cdot {\hat{y}}_{i}}{∥y_{i}∥\times ∥{\hat{y}}_{i}∥}=\frac{\sum_{i=1}^{n}y_{i}{\hat{y}}_{i}}{\sqrt{\sum_{i=1}^{n}y_{i}^{2}}\times \sqrt{\sum_{i=1}^{n}{\hat{y}}_{i}^{2}}} \end{array}$$ (16)

#### Relative effect capture metrics

##### Pearson’s correlation Δ

Pearson’s correlation Δ measures the change in the linear relationship between the predicted and actual values when adjusted for control conditions. This metric is designed to assess how the perturbation affects the correlation between predicted and actual values relative to the control condition. In particular, Pearson’s correlation Δ is calculated by first subtracting the control values from both the predicted and actual data and then computing the Pearson correlation coefficient on the adjusted data.

##### Delta direction accuracy

Delta direction accuracy evaluates a model’s ability to correctly predict the directionality of gene expression changes under perturbation conditions. This metric assesses whether genes are predicted to be up-regulated or down-regulated in the same direction as observed experimentally. For each perturbation condition, we first compute the expression changes relative to the control by calculating delta values for both predicted and true expression profiles. The delta direction accuracy is then defined as the proportion of genes where the predicted and observed expression changes have the same directional sign, calculated as

$$\Delta \;\text{Agreement}\;\text{acc}.=\frac{1}{n}\sum_{i=1}^{n}\;I\left[\text{sign}\left(\Delta {\hat{y}}_{i}\right)=\text{sign}\left(\Delta y_{i}\right)\right]$$ (17)

where

$$\Delta {\hat{y}}_{i}={\hat{y}}_{i,\text{pert}}-{\hat{y}}_{i,\text{ctrl}},\;\Delta y_{i}=y_{i,\text{pert}}-y_{i,\text{ctrl}}$$ (18)

represent the predicted and true expression changes for gene *i*, respectively. Here, ${\hat{y}}_{i,\text{pert}}$ and $y_{i,\text{pert}}$ denote the mean predicted and true expression values under perturbation, respectively, while $y_{i,\text{ctrl}}$ represents the mean control expression. The indicator function I[⋅] equals 1 when the condition is true and 0 otherwise, and sign(⋅) denotes the sign function. This metric takes values of 0 or 1, where 1 indicates perfect directional agreement and 0 indicates directional disagreement.

#### DE recovery metrics

##### DE overlap accuracy

DE overlap accuracy evaluates how well perturbation prediction models recover the correct DEGs compared to ground truth experimental data. This metric assesses the biological relevance of predictions by measuring the concordance between predicted and observed transcriptional responses.

For each perturbation condition, we first identify DEGs by comparing both predicted and true perturbed expression profiles against their respective control conditions using the Wilcoxon rank-sum test. DEGs are filtered on the basis of adjusted *P* values (Benjamini-Hochberg correction) with a significance threshold of $P_{\text{adj}}<0.05$ and ranked by absolute log fold change. The DE overlap accuracy is computed at multiple granularity levels by comparing the top-ranked DEGs between predicted and true conditions. For each top-*N* threshold (N∈10,20,50,100,200,500), the metric is defined as

$${\text{Accuracy}}_{\text{Top}-N}=\frac{|{\text{DEG}}_{\text{true}}^{\left(N\right)}\cap {\text{DEG}}_{\text{pred}}^{\left(N\right)}|}{N}$$ (19)

where ${\text{DEGtrue}}^{\left(N\right)}$ and ${\text{DEGpred}}^{\left(N\right)}$ represent the sets of top-*N* DEGs from true and predicted data, respectively, and ∣⋅∣ denotes set cardinality. If the number of significant DEGs in either the predicted or true condition is less than *N*, the accuracy for that *N* is not calculated. A maximum of 500 cells per condition is used, with random sampling applied when necessary. The metric ranges from 0 to 1, with 1 indicating complete overlap between predicted and true DEGs.

##### Effect size

Effect size is defined as the number of significant DEGs per condition ($N_{\text{sig}}$, $P_{\text{adj}}<0.05$). The distribution of effect sizes in the true datasets is examined to characterize the transcriptional response across conditions. The relationship between predicted and true effect sizes is quantified using the Spearman rank correlation

$$\rho_{\text{Spearman}}=\text{rank}\;\text{correlation}\left(N_{\text{sig}}^{\text{true}},N_{\text{sig}}^{\text{pred}}\right)$$ (20)

where each vector contains paired effect sizes for all conditions in a dataset. Conditions are excluded from the calculation if there are fewer than five valid paired observations or if either the predicted or true values have zero variance.

#### Distributional similarity metrics

##### Maximum mean discrepancy (MMD)

MMD is used to measure the distance between two distributions, making it suitable for evaluating the difference between predicted and actual distributions. In particular, we computed per gene (i.e., it quantifies the distributional distance between the predicted and observed expression values across all cells for that gene). After obtaining one MMD value for every gene, we then aggregate these per-gene distances to yield a single scalar that summarizes the overall distributional fidelity for the perturbation condition. It calculates the mean discrepancy between the predicted and actual data using a kernel function, defined as

$${\text{MMD}}^{2}=E\left[k\left(y_{i},y_{i}^{\prime }\right)\right]+E\left[k\left({\hat{y}}_{i},{\hat{y}}_{i^{\prime }}^{\prime }\right)\right]-2E\left[k\left(y_{i},{\hat{y}}_{i}\right)\right]$$ (21)

Here, $y_{i}$ and ${\hat{y}}_{i}$ represent samples from two different distributions, while $y_{i}^{\prime }$ and ${\hat{y}}_{i}^{\prime }$ are samples within the same distribution. $k\left(y_{i},{\hat{y}}_{i}\right)$ is the kernel function, and we use the radial basis function kernel in this study.

##### Wasserstein distance

Wasserstein distance is used to measure the geographical distance between two probability distributions and is commonly used to assess the similarity between distributions. By solving the optimal transport problem, it calculates the cumulative discrepancy between the two distributions, defined as

$$W\left(P,Q\right)=\underset{\gamma \in \Pi \left(P,Q\right)}{\text{inf}}E_{\left(y_{i},{\hat{y}}_{i}\right)∼\gamma }\left[∥y_{i}-{\hat{y}}_{i}∥\right]$$ (22)

In the formula, P and Q are two different probability distributions, and γ∈Π(P,Q) represent the set of all joint distributions from P to Q.

#### Ranking-based meta-analysis

We conducted a comprehensive ranking-based meta-analysis to evaluate model performance across four standardized dimensions. The evaluation framework comprised four key dimensions, each weighted equally at 25%: (i) absolute accuracy, measuring prediction accuracy through error metrics (MSE, RMSE, *L*_2_, and MAE) and correlation metrics (Pearson correlation, cosine similarity, and *R*^2^); (ii) relative effect capture, assessing perturbation effect detection using Pearson correlation delta and delta agreement accuracy; (iii) DE recovery, evaluating DEG identification capability through top 50 accuracy and Spearman correlation; and (iv) distribution similarity, measuring distributional fidelity via MMD and Wasserstein distance. For the first, second, and fourth dimensions, metrics were calculated for both all genes and the top 20 DEGs, while the third dimension used all genes only.

For score calculation, models were ranked within each metric for each task separately. Error-based metrics (MSE, RMSE, *L*_2_, MAE, MMD, Wasserstein, and execution time) were reverse-ranked, where lower values received higher normalized scores. Each dimension score was computed as the average of normalized rank scores for all constituent metrics within that dimension. The overall score was calculated as the unweighted average of the four dimension scores, excluding time and user-friendliness metrics, which were displayed separately for reference. Missing values were imputed using the mean value of the respective metric across all models within each task to ensure complete comparisons.

The visualization used a multielement approach where point size represents normalized metric values within each column (larger points indicating higher values), point color indicates rank within each column using the RdGy color map (darker colors representing better ranks), horizontal bar charts display overall scores with numerical labels, and star symbols represent user-friendliness levels (1 star, good; 2 stars, better). This ranking-based meta-analysis provides task-specific model comparisons while maintaining consistency across evaluation criteria and effectively handling the heterogeneity of available metrics across different models and datasets.

### TRADE

To assess the transcriptome-wide impact of perturbations, we used the TRADE (TRADEtools version 0.99.0) (*63*) method, which quantifies the total effect of a perturbation across the entire transcriptome. TRADE addresses the challenges of noise and undetected effects that are common in single-cell CRISPR screens, such as Perturb-seq, by providing a robust estimation of the true DE effects. The analysis was performed following the tutorial provided by the authors. DE analysis was carried out using DESeq2 (version 1.42.1), incorporating both condition and batch information to account for potential variations between replicates. The DE results were then integrated into the TRADE framework, which calculates a transcriptome-wide impact score. This score reflects the global shift in gene expression distributions caused by the perturbation relative to the control condition. TRADE’s capability to summarize these effects enables the systematic ranking and comparison of perturbations across multiple datasets.

### E-distance

E-distance is a metric used to measure the difference between two distributions in a high-dimensional space, defined as the mean squared Euclidean distance between samples. In this study, we use E-distance to quantify the high-dimensional distribution differences across different datasets using scPerturb (version 0.1.0) for the calculations. For samples originating from two distributions, we first compute the Euclidean distance between each pair of samples and then calculate the average distance within each distribution as well as the average distance between the two distributions. Before these calculations, we standardize the single-cell data, filter out low-expression cells (min_counts = 100) and low-expression genes (min_cells = 50), and select the top 2000 highly variable genes as features for analysis. Detailed methodologies can be referred to in the scPerturb (*8*) publication.

### Statistical analysis

All statistical analyses were conducted using Python (version 3.12.3) with the pandas (version 1.5.3), statsmodels (version 0.14.2), and NumPy (1.26.4) libraries. For each task, we performed ANOVA tests to assess the significance of performance differences across models and various experimental factors specific to each task design. Task-specific ANOVA models were implemented using ordinary least squares regression, where all variables were treated as categorical factors using the C() notation in statsmodels.

For Task 1, we used the model

value∼C(perturbation)+C(dataset)+C(model)

where perturbation, dataset, and model served as categorical factors.

For Task 2, we used

value∼C(model)+C(seen level)+C(additive type)

For Task 3, we used

value∼C(model)+C(perturbation)+C(cell_type)+C(dataset)

Post hoc analysis with Tukey’s post hoc test was conducted to perform pairwise comparisons between models when a significant effect (P<0.05) was detected. All analyses were performed using type II sum of squares for ANOVA, and results were considered statistically significant at P<0.05.

In addition to the task-specific ANOVA models described above, which focus on experimental design factors, we further conducted a more granular variance decomposition analysis to better disentangle the contributions of dataset characteristics and model design choices to performance variability. For dataset-level factors, we extracted condition-specific features including (i) sequencing depth (mean counts per cell), (ii) perturbation effect size (mean absolute log_2_ fold change between perturbed and control cells), (iii) KO/KD (knockdown) efficiency for genetic perturbations (Tasks 1 and 2), and (iv) cell type heterogeneity for drug perturbations (Task 3, quantified by the Shannon diversity index). For model-level factors, we characterized (i) preprocessing strategies (normalization method, feature selection, and batch correction), (ii) loss function design (reconstruction, distributional, adversarial, and auxiliary objectives), and (iii) latent space architecture (type, dimensionality, and structure). All continuous features were categorized into three levels (low, medium, and high) using quantile-based binning. The granular ANOVA models included the following categorical factors. For Tasks 1 and 2, the model incorporated sequencing depth, perturbation effect size, KO/KD efficiency, preprocessing strategy, loss function design, and latent space architecture as main effects. For Task 3, the model incorporated sequencing depth, perturbation effect size, cell type heterogeneity, preprocessing strategy, loss function design, and latent space architecture as main effects. Variance proportions were calculated by dividing each factor’s sum of squares by the total sum of squares across all factors (excluding residuals).

### Response-component decomposition analysis

To interpret how different evaluation readouts reflect different sources of variation in postperturbation expression, we performed a response-component decomposition on the observed profiles from the common model-evaluation subset of each task. For each task, the common subset was defined by retaining only the evaluated entries shared across the corresponding prediction models. Each entry was indexed by dataset and perturbation condition, with cell type additionally included when cell state transfer was evaluated. For each entry, we computed a pseudobulk expression profile $Y_{c,p}$ by averaging normalized log-transformed expression across cells assigned to cell state or cell type *c* and perturbation condition *p*.

We represented each observed postperturbation profile using the diagnostic decomposition

$$Y_{c,p}=B_{c}+G_{c}+S_{p}+I_{p_{1},p_{2}}+C_{c,p}+\varepsilon$$

Here, $B_{c}$ denotes the baseline control expression of cell state *c*; $G_{c}$ denotes the systematic shift shared across perturbations within the same cell state; $S_{p}$ denotes the perturbation-specific response; $I_{p_{1},p_{2}}$ denotes the nonadditive interaction term for combinatorial perturbations; $C_{c,p}$ denotes the cell state–specific residual of the perturbation response, corresponding to the cell state–by–perturbation interaction term; and ε denotes the empirical noise floor.

For each dataset and task, $B_{c}$ was estimated from control cells. Perturbation-induced changes were calculated relative to $B_{c}$, and the average change within each cell state was used to estimate $G_{c}$. After removing $B_{c}$ and $G_{c}$, the remaining response was treated as the perturbation-associated residual. For tasks without cell state transfer, this residual was used as $S_{p}$. For combinatorial perturbations, $I_{p_{1},p_{2}}$ was quantified as a separate nonadditive diagnostic term, calculated as the deviation of the observed combination response from the additive expectation of the two corresponding single-perturbation responses. For cell state transfer, the residual response was further separated into a perturbation-average component shared across cell states and a cell state–specific residual $C_{c,p}$. Components that were not applicable to a given task were set to zero. The empirical noise floor ε was estimated from control cells using a split-half procedure.

Component contributions were quantified as sum-of-squares energies across evaluated entries and genes. We generated two normalized summaries. In the energy view, each component was normalized by the total postperturbation expression energy. In the centered view, expression was first centered by subtracting the gene-wise grand mean across evaluated entries, and component energies were then normalized by the centered total energy. Dataset-level fractions were averaged within each task. Because this decomposition was used as a component-wise diagnostic rather than a strictly orthogonal ANOVA partition, normalized fractions were not constrained to sum to 100%.

To relate the decomposition to model behavior, we summarized three representative evaluation readouts from the existing benchmark outputs: full-profile $Y_{c,p}$, Pearson correlation Δ, and top 50 DEG overlap accuracy. For each task-readout pair, we calculated the across-model mean and sample variance across retained prediction models. NoPerturb- and Baseline-related models were excluded from this analysis.

### Perturbation prediction on unseen genes

To assess the model’s response to perturbations in single genes that it has not seen, we use a masking strategy during model training to simulate the absence of specific genes in the training dataset. In particular, there are multiple choices for perturbed genes in each dataset, and following the same protocol as GEARS, we randomly split perturbed genes at the gene level into 75% for training and 25% for testing. During the training process, the perturbation response data for these genes are completely excluded from the training set, forcing the model to learn based solely on the perturbation features of other genes. Subsequently, the model predicts the perturbation responses for these unseen genes in the test set to evaluate its performance on unknown single-gene perturbations.

### Perturbation prediction on combinatorial perturbation

For predicting combinatorial gene perturbations, we followed the same protocol as GEARS and randomly selected multiple gene pairs from each dataset. We designed the following three training configurations to evaluate the models’ generalization capabilities under varying levels of prior knowledge: Seen0: In this configuration, neither the gene pair nor their individual single-gene perturbations appear in the training data. This setup assesses the model’s ability to predict responses to gene combinations not observed during training. Seen1: The training set includes single-gene perturbation data for one gene of the pair, while the other gene remains unseen. This configuration tests whether the model can leverage partial prior information to predict the effects of combinatorial perturbations. Seen2: The training data contain single-gene perturbation data for both genes in the pair, but their combined perturbation is not included in the training stage. This setup evaluates the model’s capacity to infer combinatorial perturbation responses on the basis of individual gene perturbation information. By randomly selecting gene pairs, we ensured that the perturbation response of each gene combination in the test set was not observed during training, thereby providing a comprehensive evaluation of its generalization performance in predicting combinatorial gene perturbations.

### Perturbation prediction on unseen cell types

In the cell state transfer prediction task, we explored the models’ generalization capabilities across different cell types. We trained the models using perturbation data from multiple cell types or cell lines to predict the perturbation responses of a target cell type that was masked during training. In particular, the training set included perturbation response data from only a subset of cell types, while data from the target cell type were completely excluded. The models were then tasked with predicting the perturbation responses for the unseen target cell type on the basis of the information learned from the training set. This approach allows us to evaluate the models’ applicability and robustness across different biological contexts.
