# Supplementary material for: A systematic comparison of single-cell perturbation response prediction models
Source: Sci Adv. 2026 Sep 9;12(37):eaed3414. doi: 10.1126/sciadv.aed3414 (PMC13557097; doi:10.1126/sciadv.aed3414)
Supplement: Supplementary file 1 [file sciadv.aed3414_sm.pdf]

Science Advances
AAAS

Supplementary Materials for

A systematic comparison of single-cell perturbation response prediction models

Lanxiang Li *et al.*

Corresponding author: Yue You, you_yue@gzlab.ac.cn; Xiaodong Liu, liuxiaodong@westlake.edu.cn; Luyi Tian, tian_luyi@gzlab.ac.cn

Sci. Adv. **12**, eaed3414 (2026)
DOI: 10.1126/sciadv.aed3414

This PDF file includes:

Figs. S1 to S27
Tables S1 to S5

Fig. S1

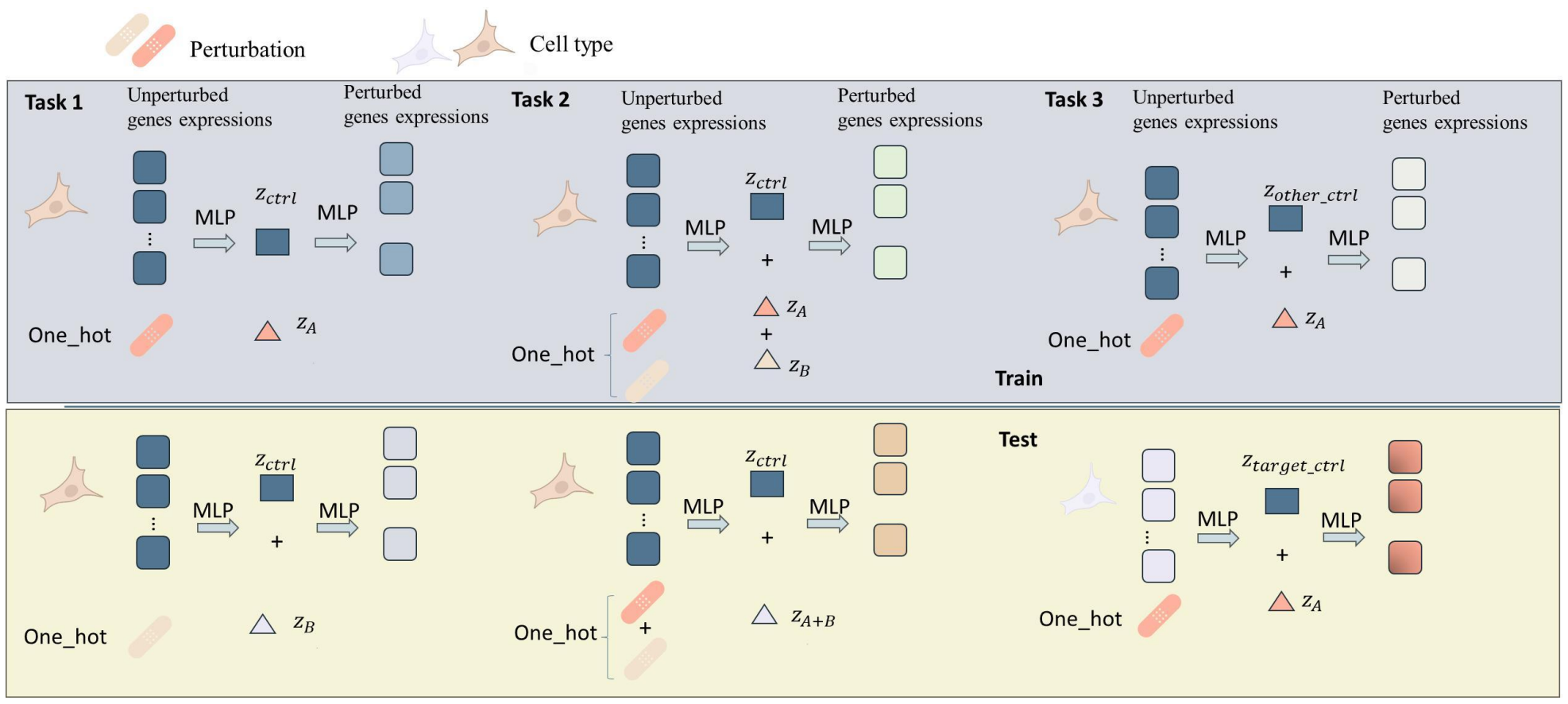

Fig. S1. Summary of Baseline_MLP model used in this study. Created in BioRender. Tian, L. (2026) `https://BioRender.com/wwdkl0n`.

Fig. S2

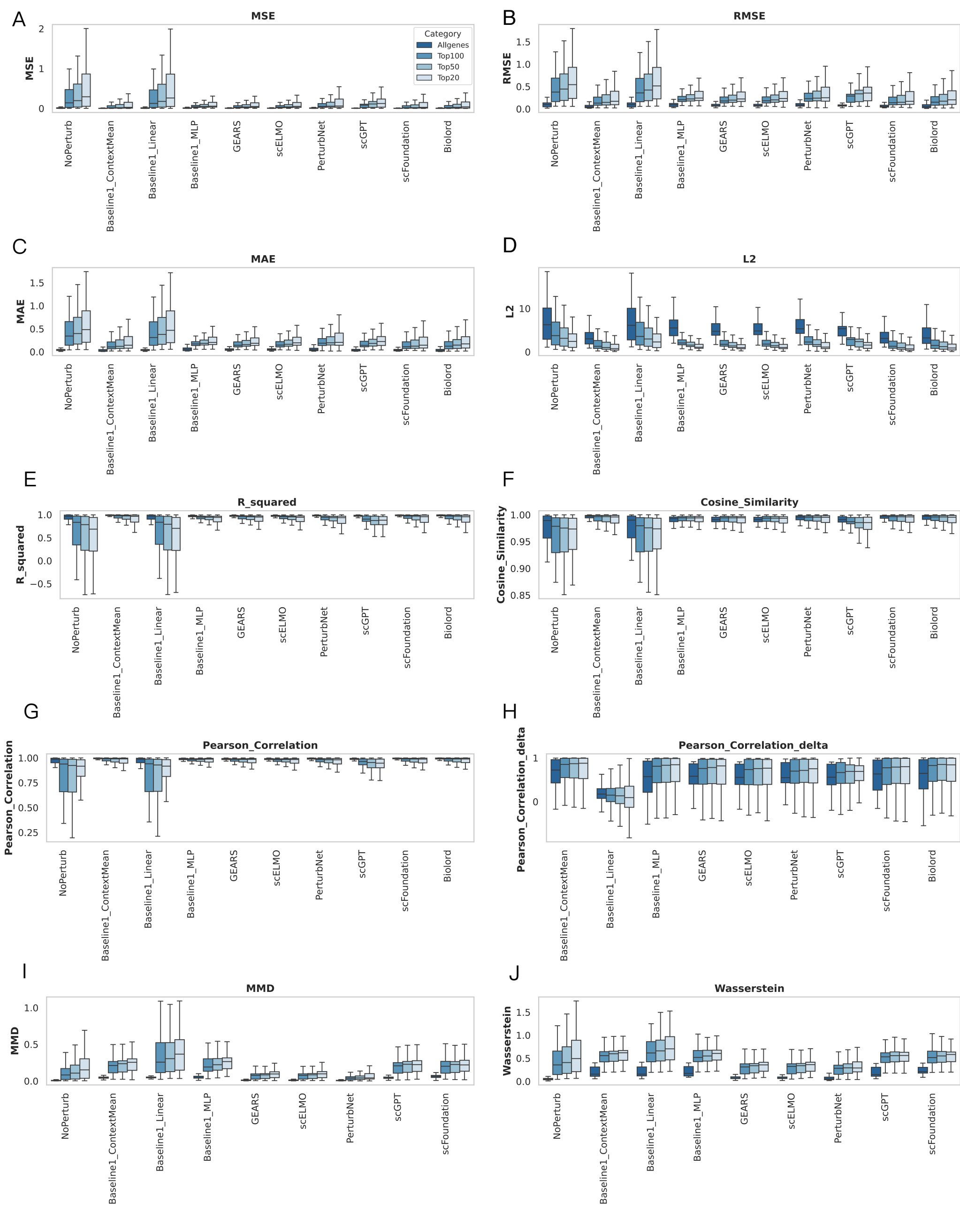

Fig. S2. Evaluation results of Task 1. Metrics aggregated on all datasets were computed across all models using four gene sets: all genes, top 100 DEGs, top 50 DEGs, and top 20 DEGs (ranked by absolute scores). Ten evaluation metrics are reported: **(A)** MSE, **(B)** RMSE, **(C)** MAE, **(D)** L2 distance, **(E)** R^2, **(F)** Cosine similarity, **(G)** Pearson correlation, **(H)** Pearson correlation delta, **(I)** MMD, and **(J)** Wasserstein distance.

Fig. S3

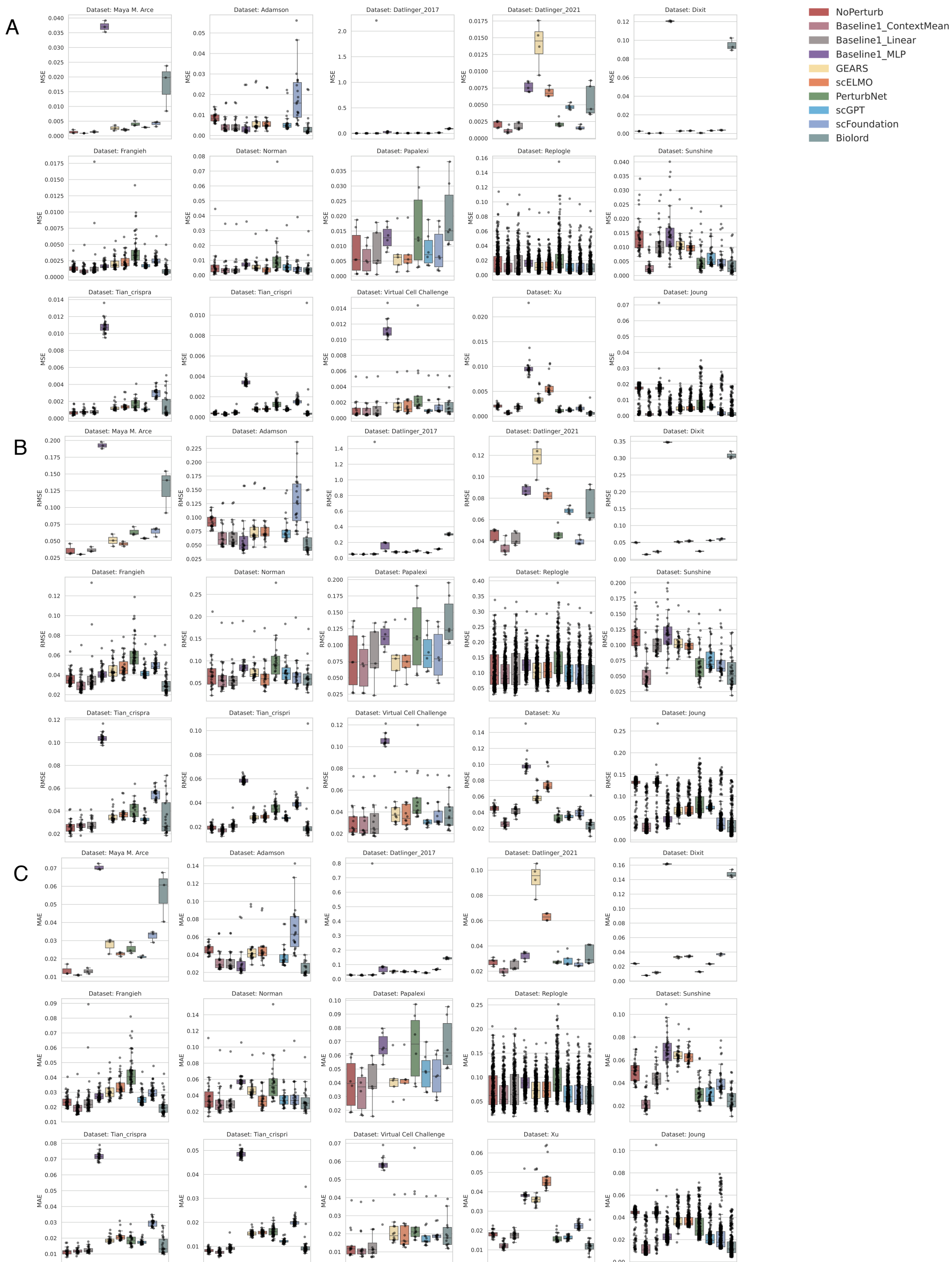

Fig. S3. Evaluation results of Task 1. Metrics were computed across all datasets and models using all genes. Each point represents a perturbation tested in the benchmark task. The evaluated datasets include Maya M. Arce, Adamson, Datlinger (2017 & 2021), Dixit, Frangieh, Norman, Papalexi, Replogle, Sunshine, Tian_crispra, Tian_crispri, Virtual Cell Challenge, Xu, and Joung. Three evaluation metrics are reported on this figure: (A) MSE, **(B)** RMSE, and **(C)** MAE.

Fig. S4

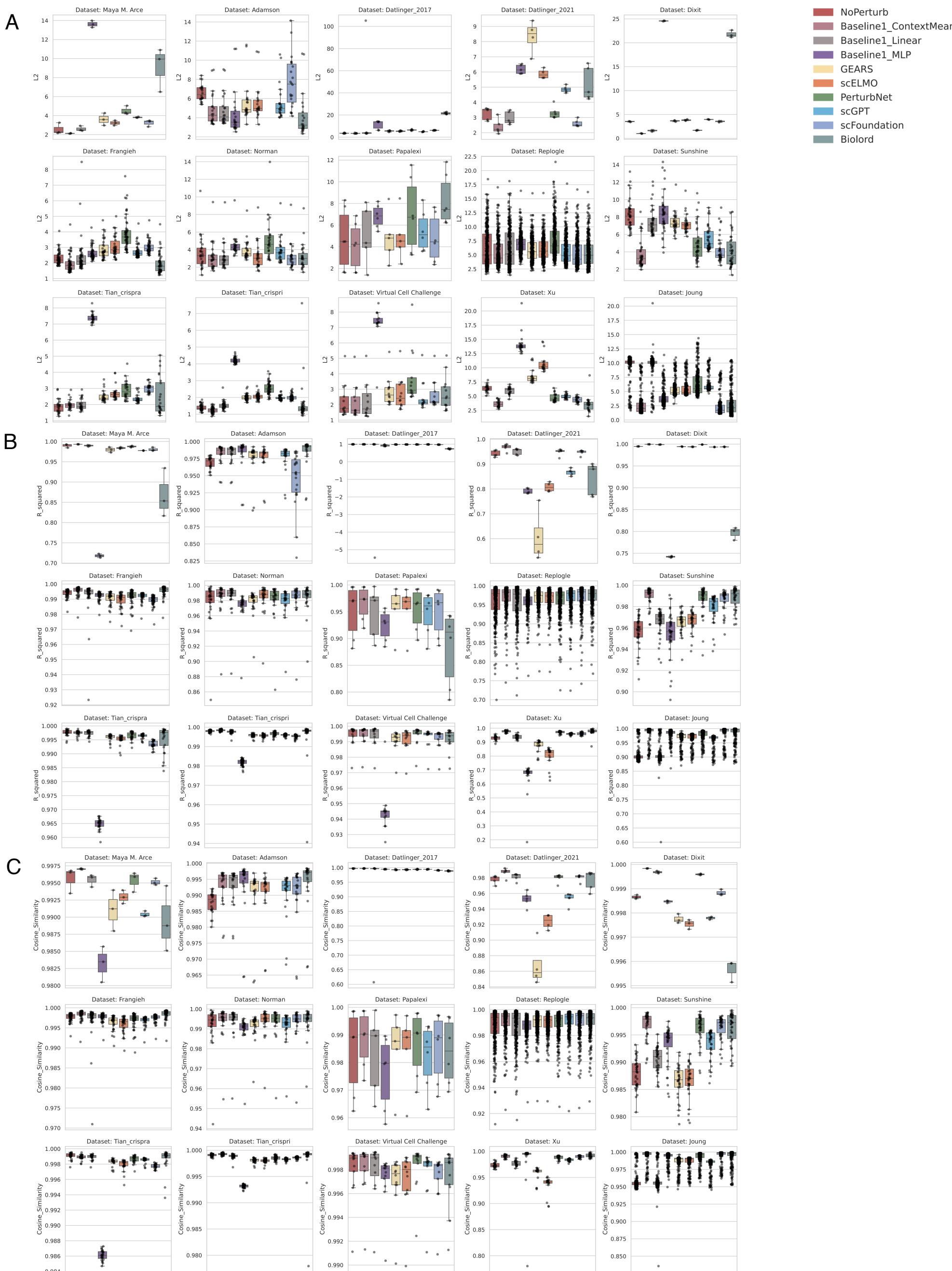

Fig. S4. Evaluation results of Task 1. Metrics were computed across all datasets and models using all genes. Each point represents a perturbation tested in the benchmark task. The evaluated datasets include Maya M. Arce, Adamson, Datlinger (2017 & 2021), Dixit, Frangieh, Norman, Papalexi, Replogle, Sunshine, Tian_crispra, Tian_crispri, Virtual Cell Challenge, Xu, and Joung. Three evaluation metrics are reported on this figure: **(A)** L2 distance, **(B)** R^2, and **(C)** Cosine similarity.

Fig. S5

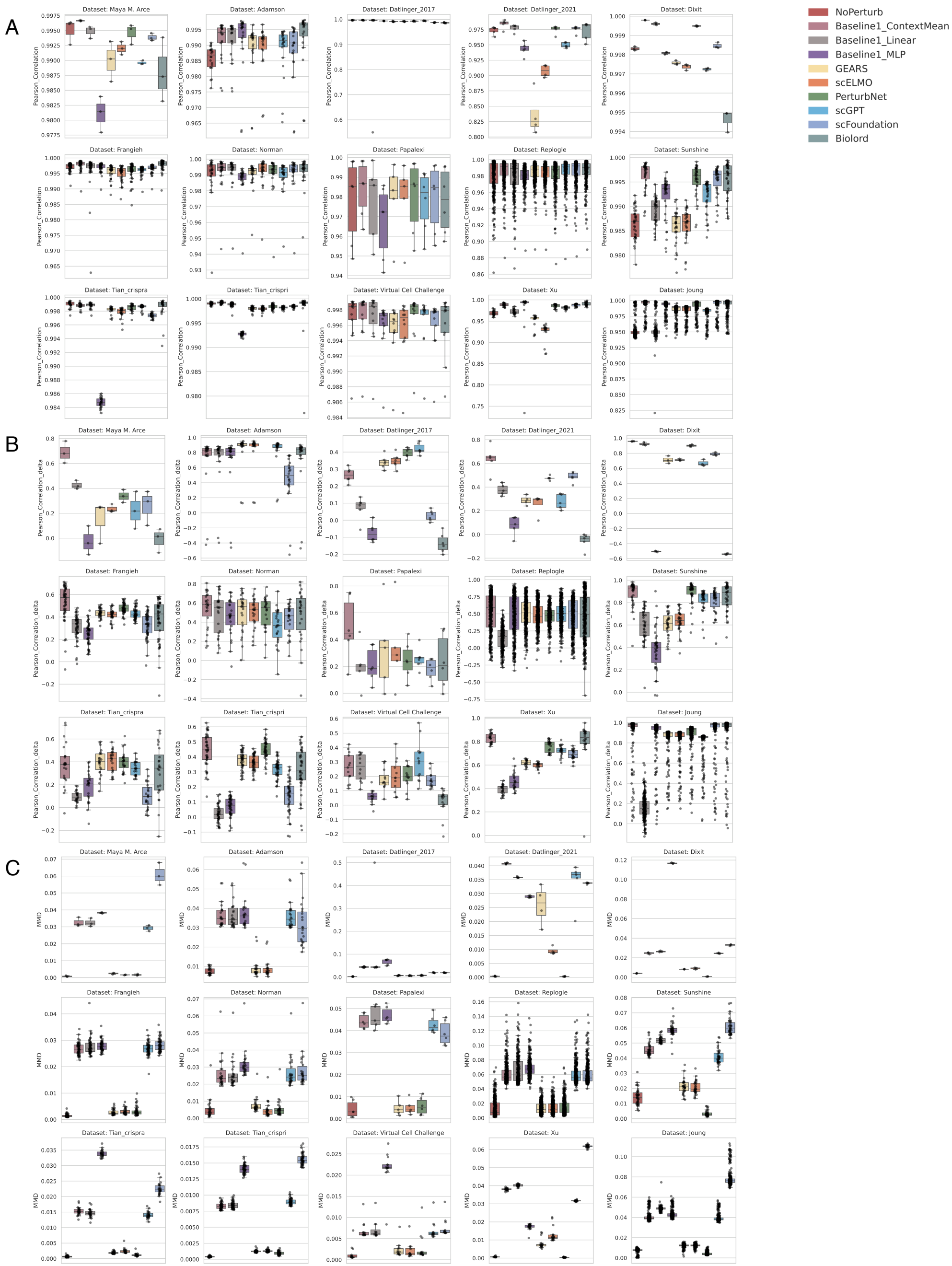

Fig. S5. Evaluation results of Task 1. Metrics were computed across all datasets and models using all genes. Each point represents a perturbation tested in the benchmark task. The evaluated datasets include Maya M. Arce, Adamson, Datlinger (2017 & 2021), Dixit, Frangieh, Norman, Papalexi, Replogle, Sunshine, Tian_crispra, Tian_crispri, Virtual Cell Challenge, Xu, and Joung. Three evaluation metrics are reported on this figure: (A) Pearson correlation, **(B)** Pearson correlation delta, and **(C)** MMD.

Fig. S6

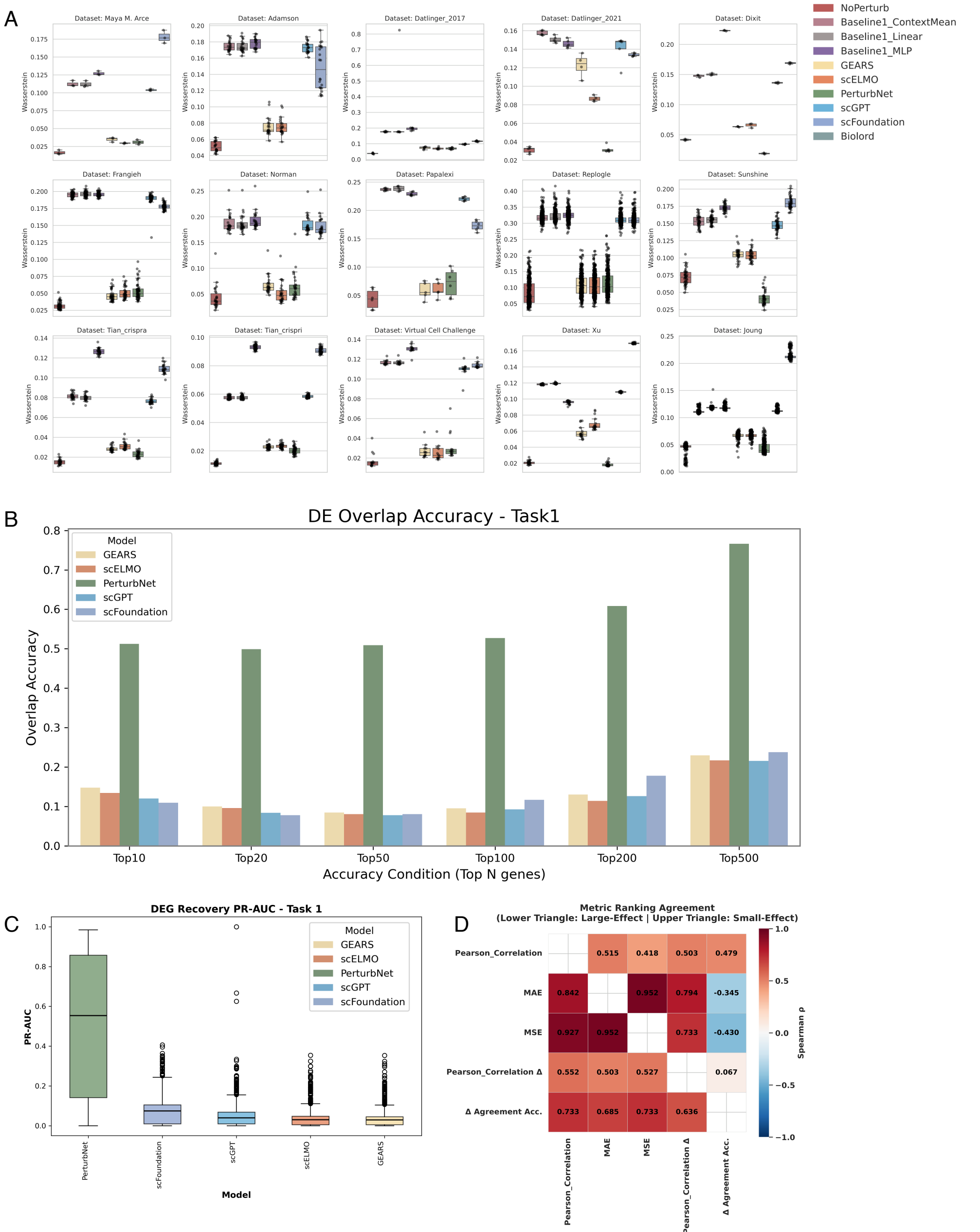

Fig. S6. Evaluation results of Task 1. (A) Wasserstein distance across all datasets and models using all genes. Each point represents a perturbation tested in the benchmark task. Datasets include Maya M. Arce, Adamson, Datlinger (2017 & 2021), Dixit, Frangieh, Norman, Papalexi, Replogle, Sunshine, Tian_crispra, Tian_crispri, Virtual Cell Challenge, Xu, and Joung. **(B)** Differentially expressed (DE) gene overlap accuracy between true and predicted perturbations versus control, aggregated on all datasets across all models. **(C)** auPRC for differential expression (DE) recovery between true and predicted perturbations versus control, aggregated across all datasets and models. Observed DE genes are used as ground truth, and predicted genes are ranked by FDR. **(D)** Heatmap of Spearman rank correlations between evaluation metrics. Lower triangle: large-effect perturbations; upper triangle: small-effect perturbations.

Fig. S7

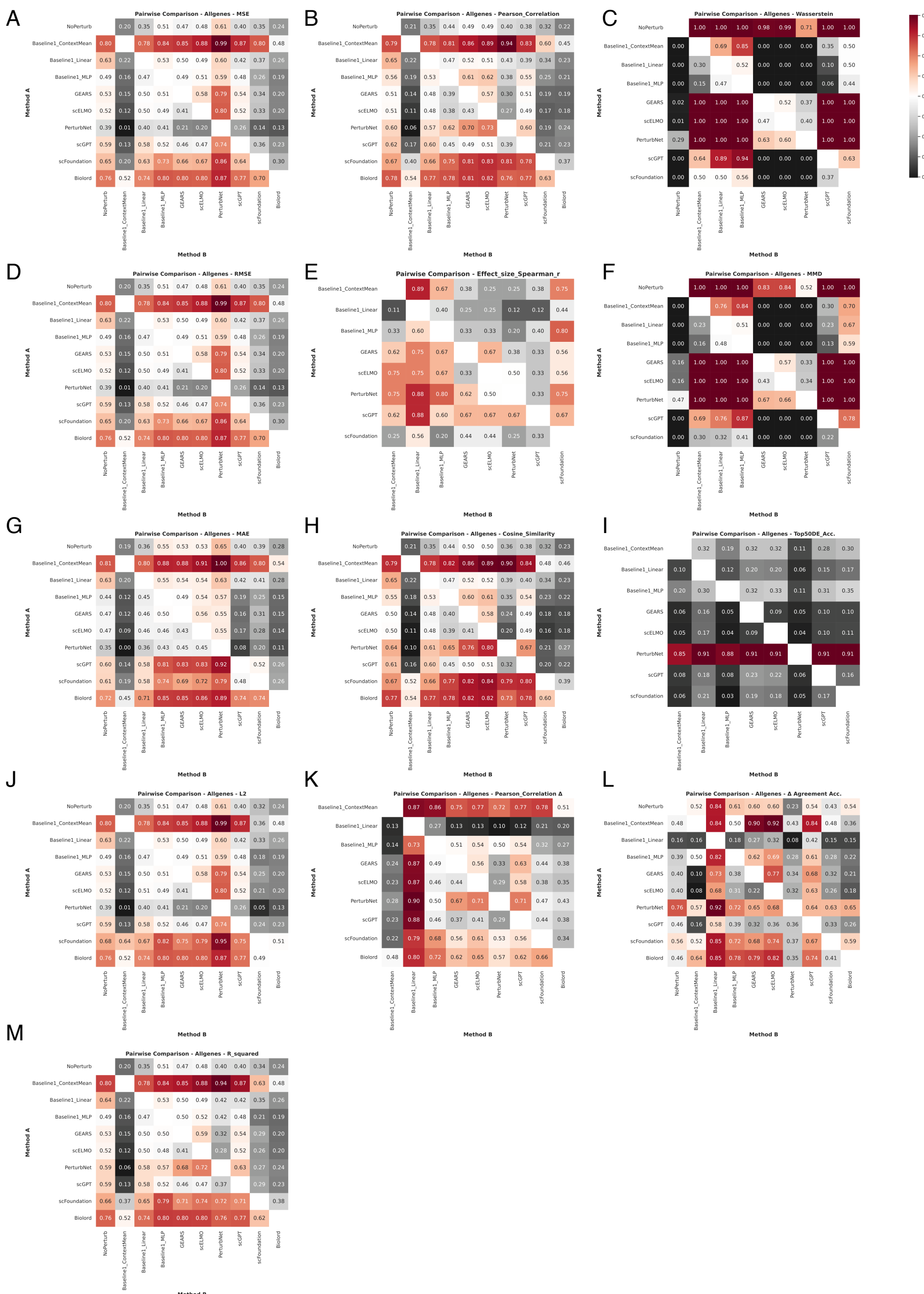

Fig. S7. Evaluation results of Task 1. (A-M) Pairwise outperformance heatmaps. Each panel corresponds to one evaluation metric: **(A)** MSE, **(B)** Pearson correlation, **(C)** Wasserstein distance, **(D)** RMSE, **(E)** Effect size correlation (Spearman r), **(F)** MMD, **(G)** MAE, **(H)** Cosine similarity, **(I)** Top 50 DE gene overlap accuracy, **(J)** L2 distance, **(K)** Pearson correlation delta, **(L)** delta direction agreement accuracy, and **(M)** R^2. Each cell (A, B) shows the fraction of dataset–perturbation conditions in which method A achieves a better rank than method B for the corresponding metric. Rankings are computed independently within each condition across all models, with ties handled using dense ranking.

Fig. S8

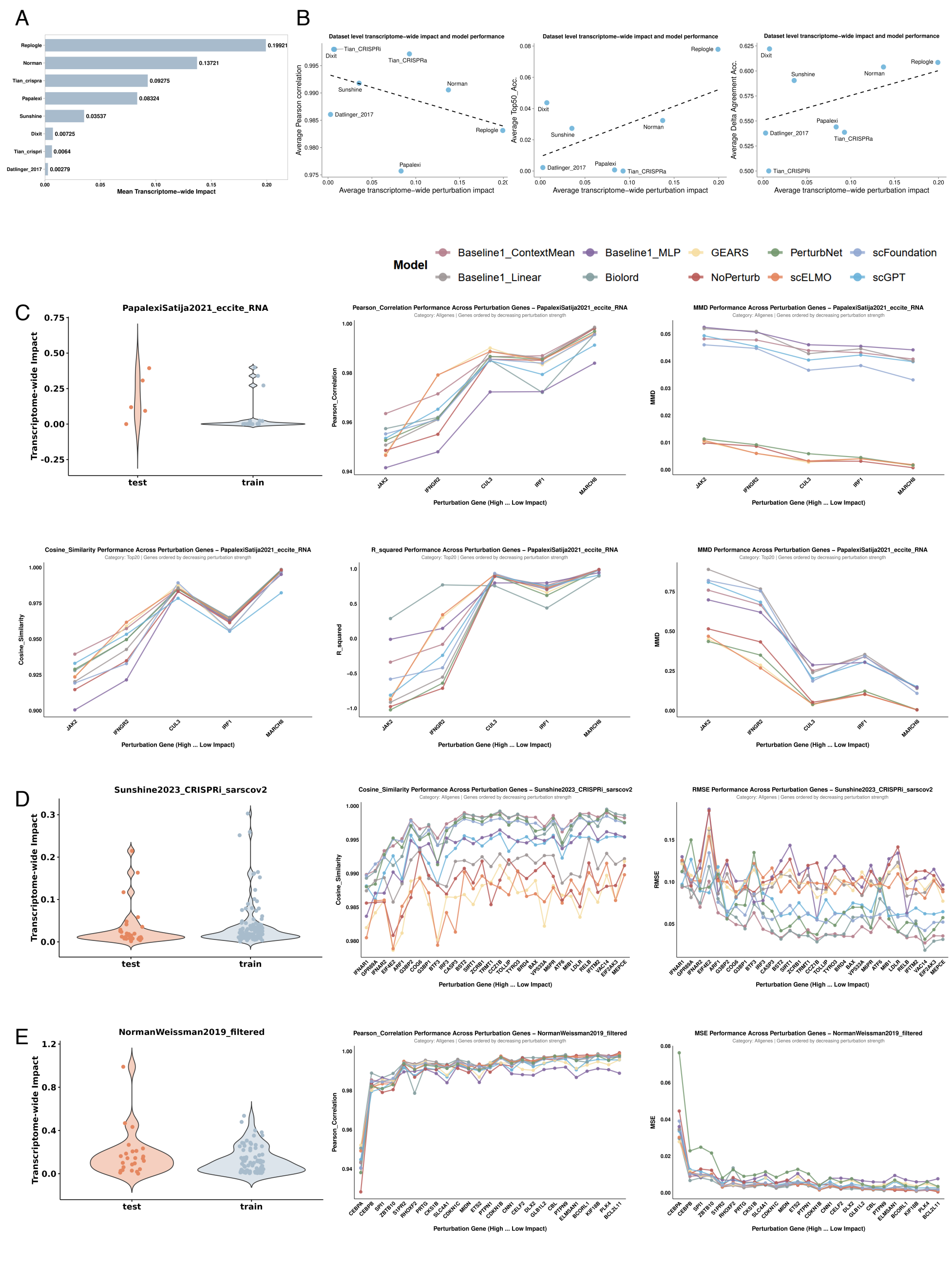

Fig. S8. Evaluation results of Task 1. **(A)** Mean transcriptome-wide impact across datasets. **(b)** Dataset-level relationship between average perturbation impact and average model performance for Pearson correlation (left), top 50 DE gene overlap accuracy (middle), and delta direction agreement accuracy (right). Each point is a dataset (labeled), with a linear fit line. **(C–E)** Transcriptome-wide impact distributions on train and test data for individual datasets (left). Metrics calculated across models on gene perturbations: **(C)** R^2, Cosine similarity, Pearson correlation, and MMD on the Papalexi dataset; **(D)** Cosine similarity and RMSE on the Sunshine dataset; and **(E)** MSE and Pearson correlation on the Norman dataset. Perturbed genes are ordered from highest to lowest transcriptome-wide impact.

Fig. S9

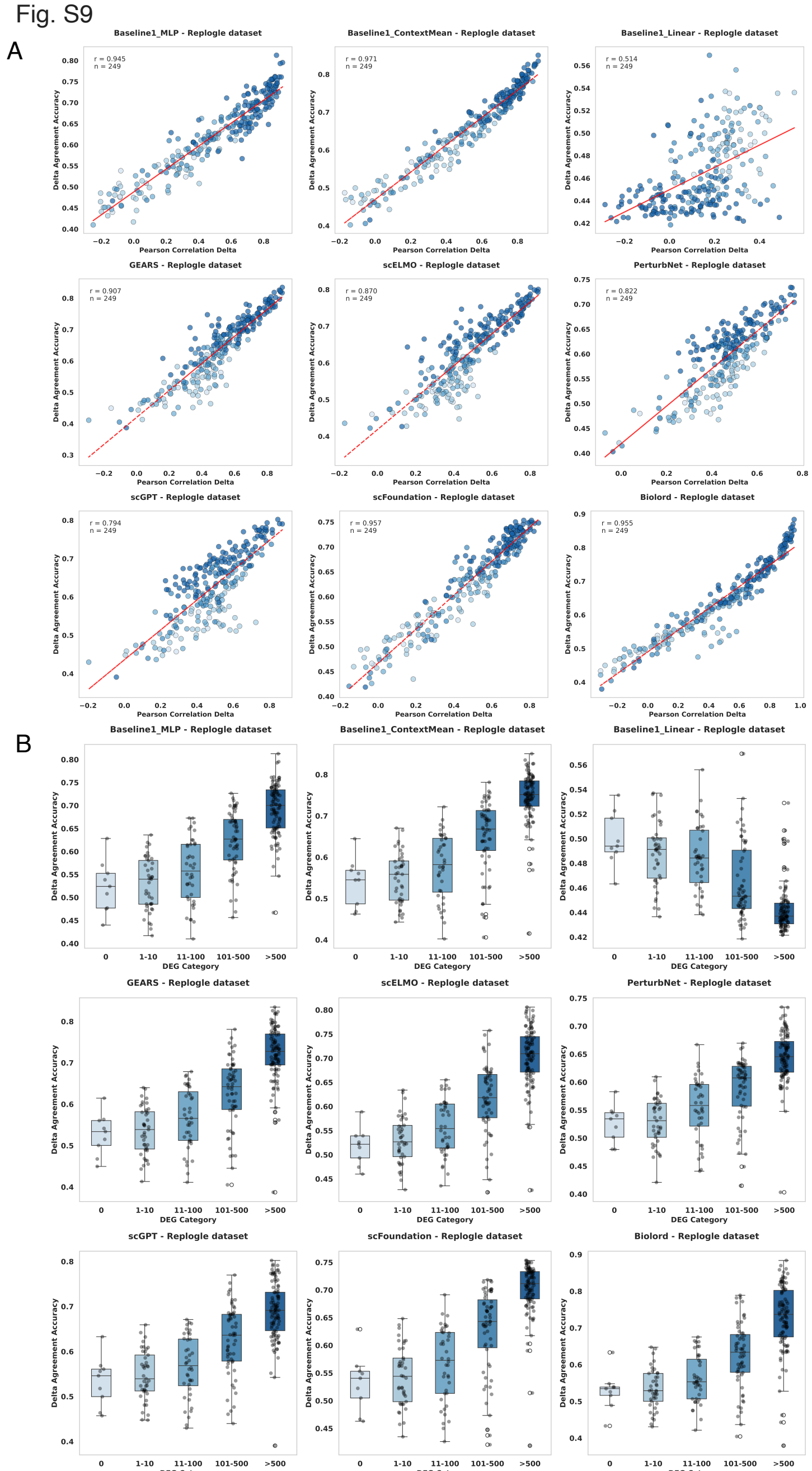

Fig. S9. Evaluation results of Task 1. **(A)** Delta direction agreement and prediction accuracy for the Baselines, GEARS, scELMO, scGPT, scFoundation, and Biolord models on the Repologle dataset. **(B)** Delta direction agreement across different effect size groups for the Baselines, GEARS, scELMO, scGPT, scFoundation, and biolord models on the Repologle dataset.

Fig. S10

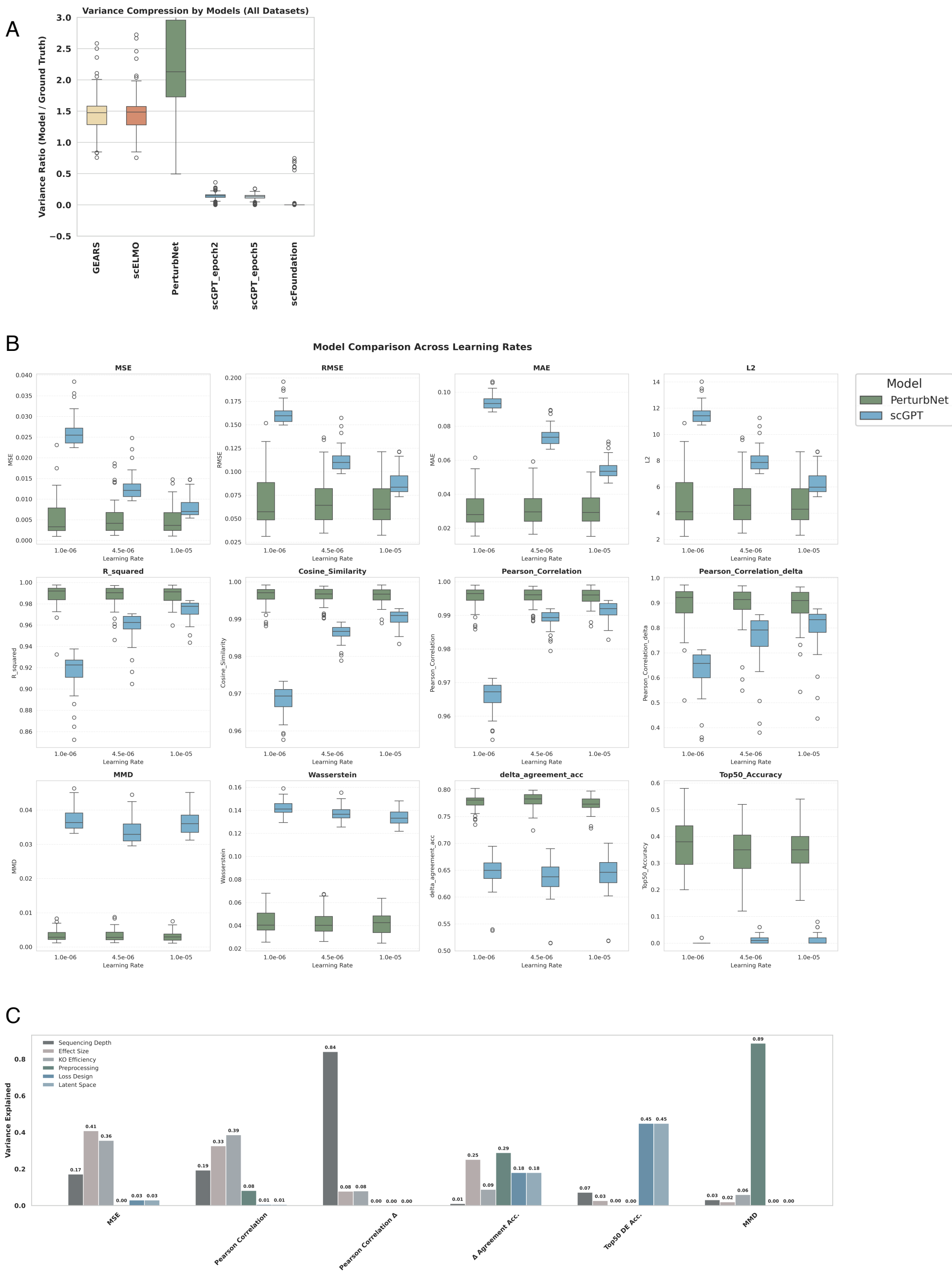

Fig. S10. Evaluation results of Task 1. (A) Variance compression across perturbations and models. Boxplots show variance ratios (predicted / ground-truth) for each model across all perturbations and datasets. **(B)** Sensitivity analysis of learning rate for PerturbNet and scGPT on the Sunshine dataset. Boxplots show performance across a range of learning rates (1e-6, 4.5e-6, 1e-5) for PerturbNet (green) and scGPT (blue) across multiple metrics. Performance rankings between the two models remain consistent across all learning rates, demonstrating that our main conclusions are robust to modest hyperparameter variation. **(C)** Variance decomposition analysis showing the relative contribution of dataset-level factors (sequencing depth, perturbation effect size, and KO efficiency) and model-level factors (preprocessing strategy, loss function design, and latent space architecture) to prediction performance across evaluation metrics.

Fig. S11

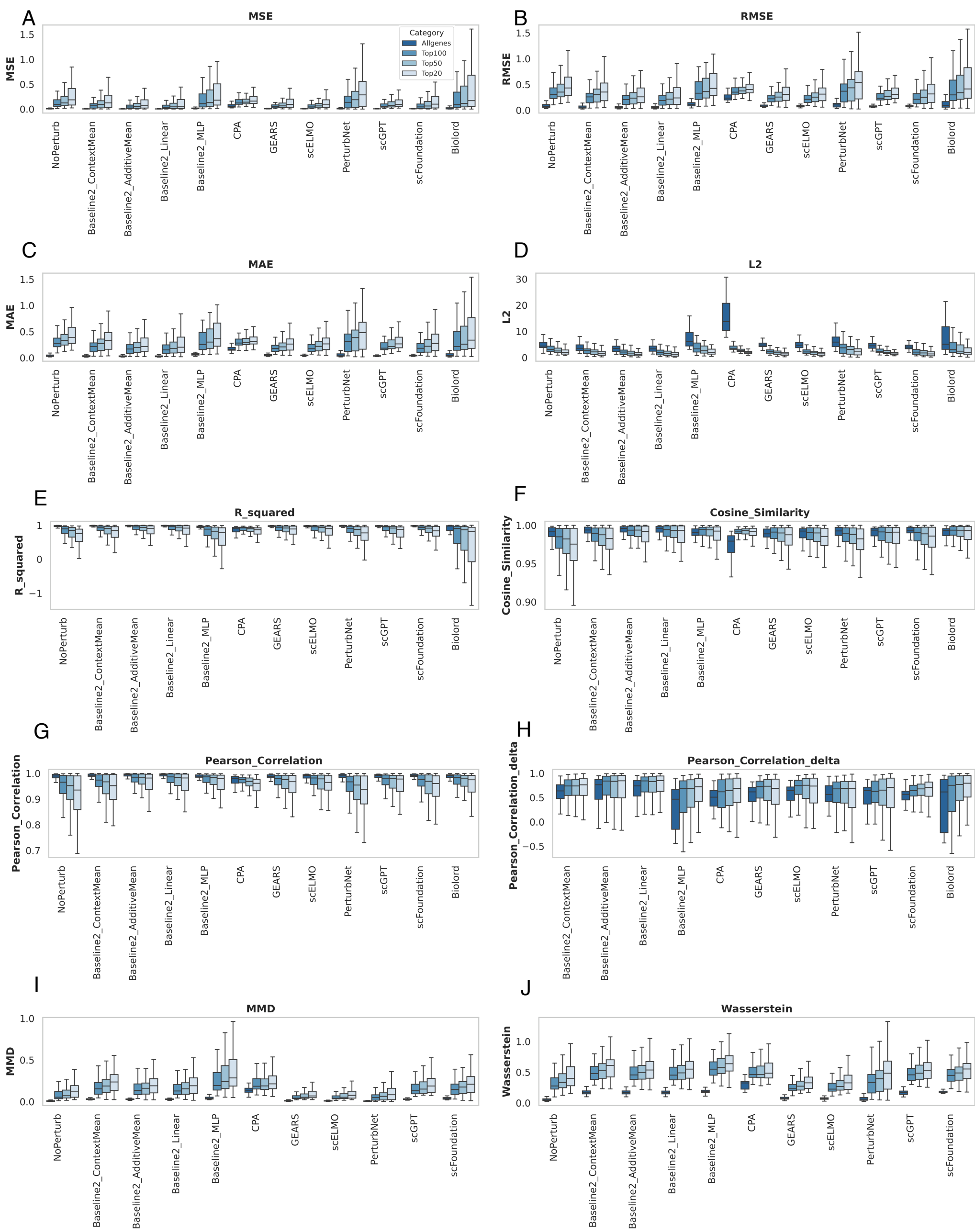

Fig. S11. Evaluation results of Task 2. Metrics aggregated on all datasets were computed across all models using four gene sets: all genes, top 100 DEGs, top 50 DEGs, and top 20 DEGs (ranked by absolute scores). Ten evaluation metrics are reported: **(A)** MSE, **(B)** RMSE, **(C)** MAE, **(D)** L2 distance, **(E)** R^2, **(F)** Cosine similarity, **(G)** Pearson correlation, **(H)** Pearson correlation delta, **(I)** MMD, and **(J)** Wasserstein distance.

Fig. S12

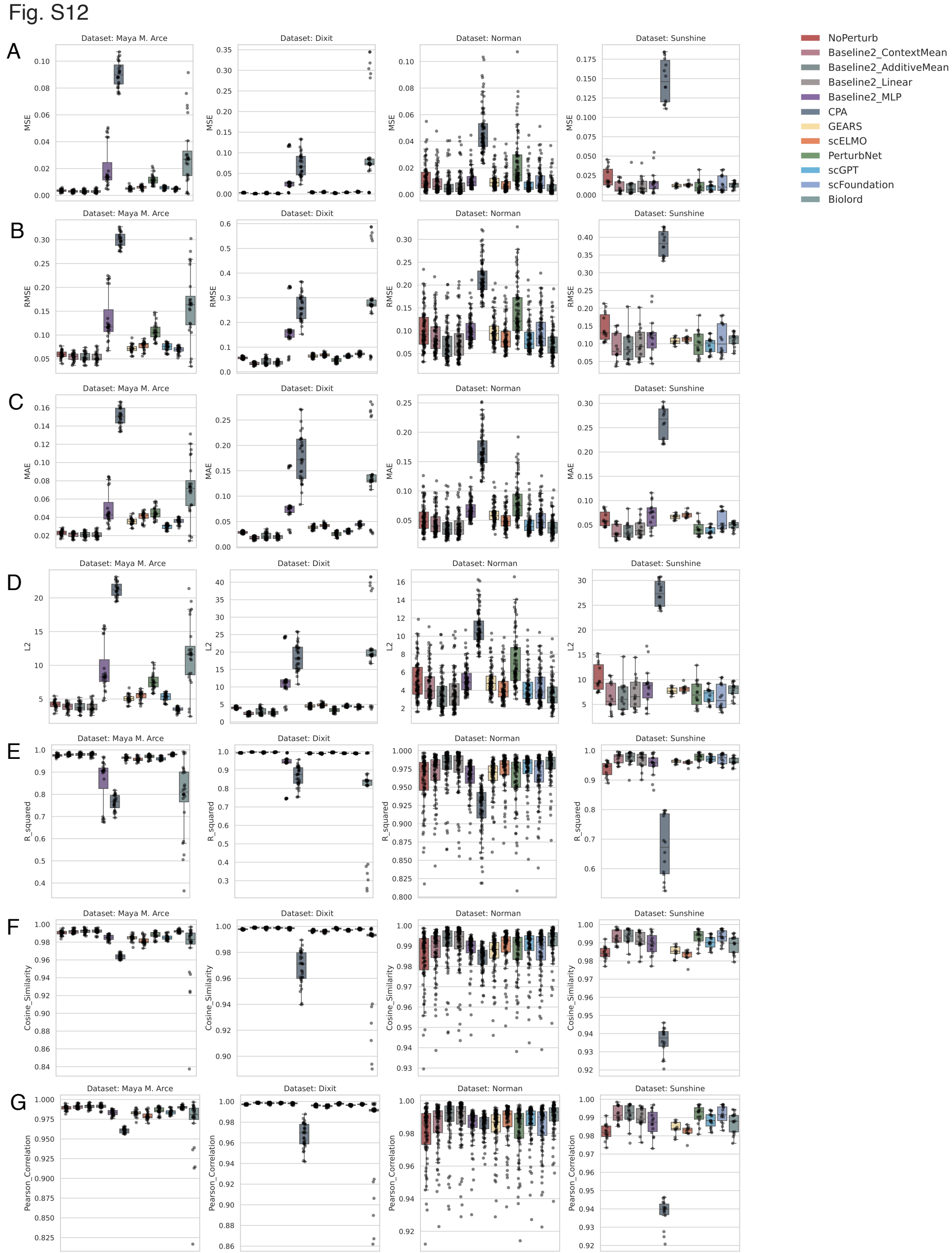

Fig. S12. Evaluation results of Task 2. Metrics were computed across all datasets and models using all genes. Each point represents a perturbation tested in the benchmark task. The evaluated datasets include Maya M. Arce, Dixit, Norman, and Sunshine. Seven evaluation metrics are reported on this figure: **(A)** MSE, **(B)** RMSE, **(C)** MAE, **(D)** L2 distance, **(E)** R^2, **(F)** Cosine similarity, and **(G)** Pearson correlation.

Fig. S13

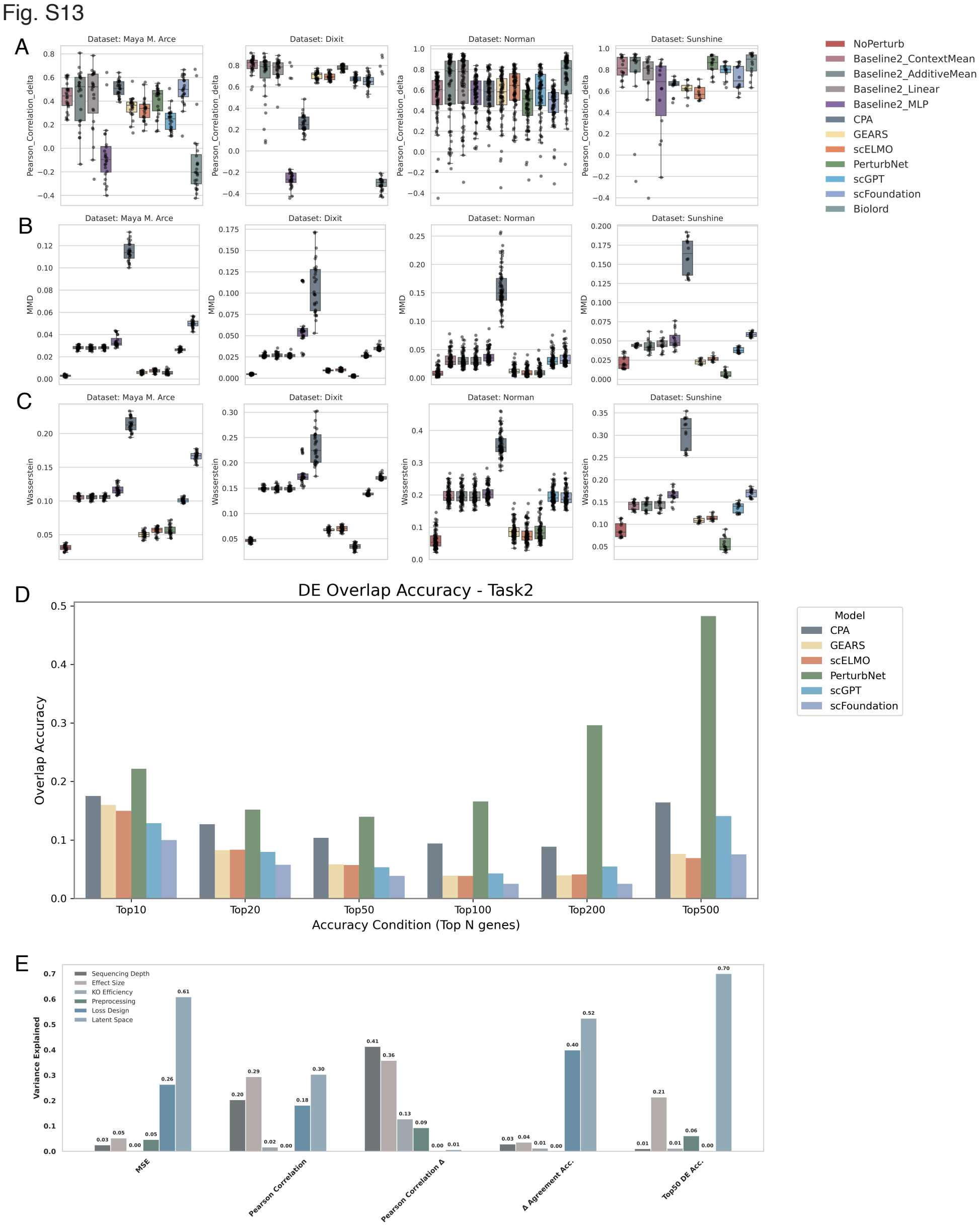

Fig. S13. Evaluation results of Task 2. **(A)** Pearson correlation delta, **(B)** MMD, and **(C)** Wasserstein distance across all datasets and models using all genes. Each point represents a perturbation tested in the benchmark task. Datasets include Maya M. Arce, Dixit, Norman, and Sunshine. **(D)** Differentially expressed (DE) gene overlap accuracy between true and predicted perturbations versus control, aggregated on all datasets across all models. **(E)** Variance decomposition analysis showing the relative contribution of dataset-level factors (sequencing depth, perturbation effect size, and KO efficiency) and model-level factors (preprocessing strategy, loss function design, and latent space architecture) to prediction performance across evaluation metrics.

Fig. S14

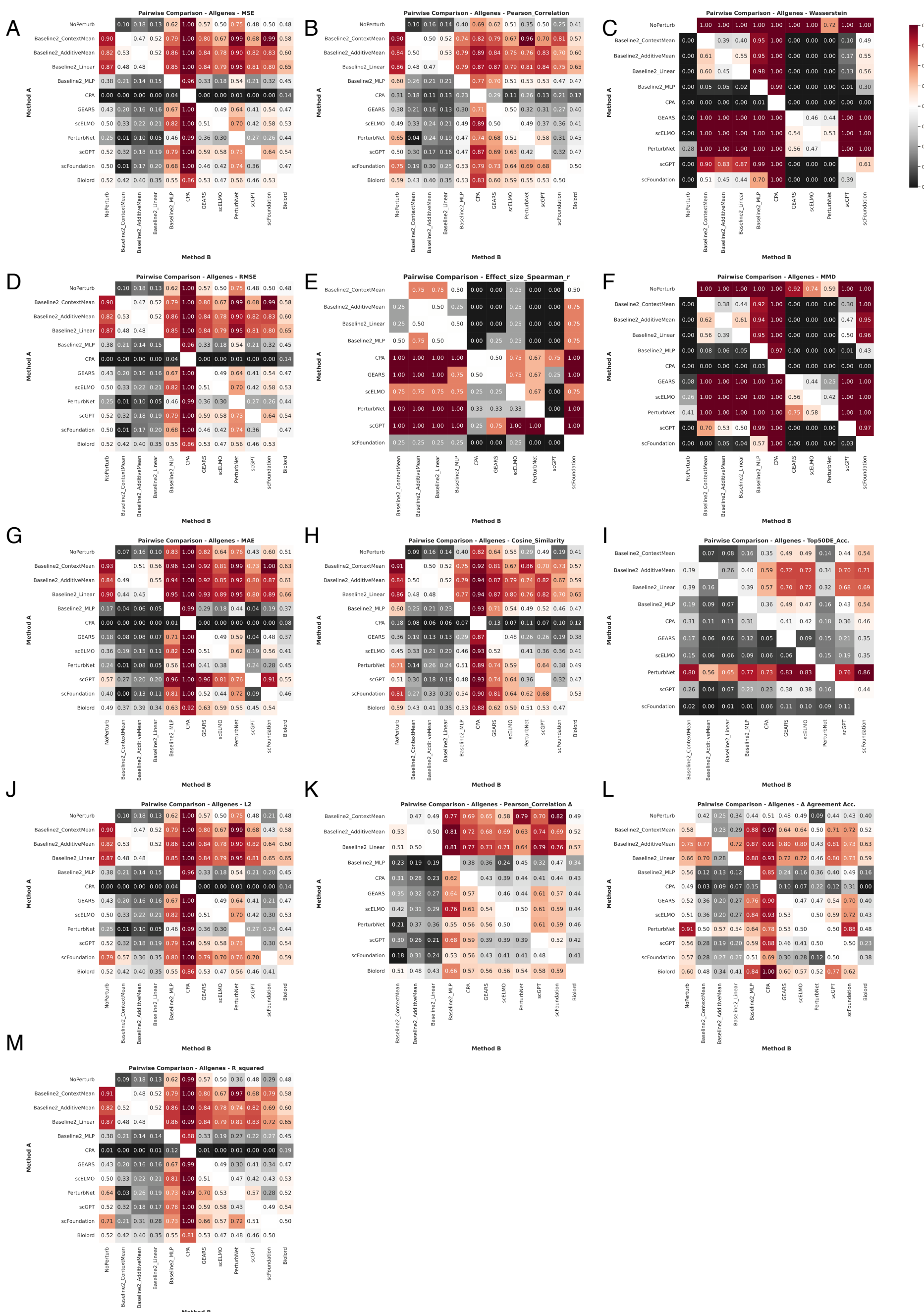

Fig. S14. Evaluation results of Task 2. (A-M) Pairwise outperformance heatmaps. Each panel corresponds to one evaluation metric: **(A)** MSE, **(B)** Pearson correlation, **(C)** Wasserstein distance, **(D)** RMSE, **(E)** Effect size correlation (Spearman r), **(F)** MMD, **(G)** MAE, **(H)** Cosine similarity, **(I)** Top 50 DE gene overlap accuracy, **(J)** L2 distance, **(K)** Pearson correlation delta, **(L)** delta direction agreement accuracy, and **(M)** R^2. Each cell (A, B) shows the fraction of dataset–perturbation conditions in which method A achieves a better rank than method B for the corresponding metric. Rankings are computed independently within each condition across all models, with ties handled using dense ranking.

Fig. S15

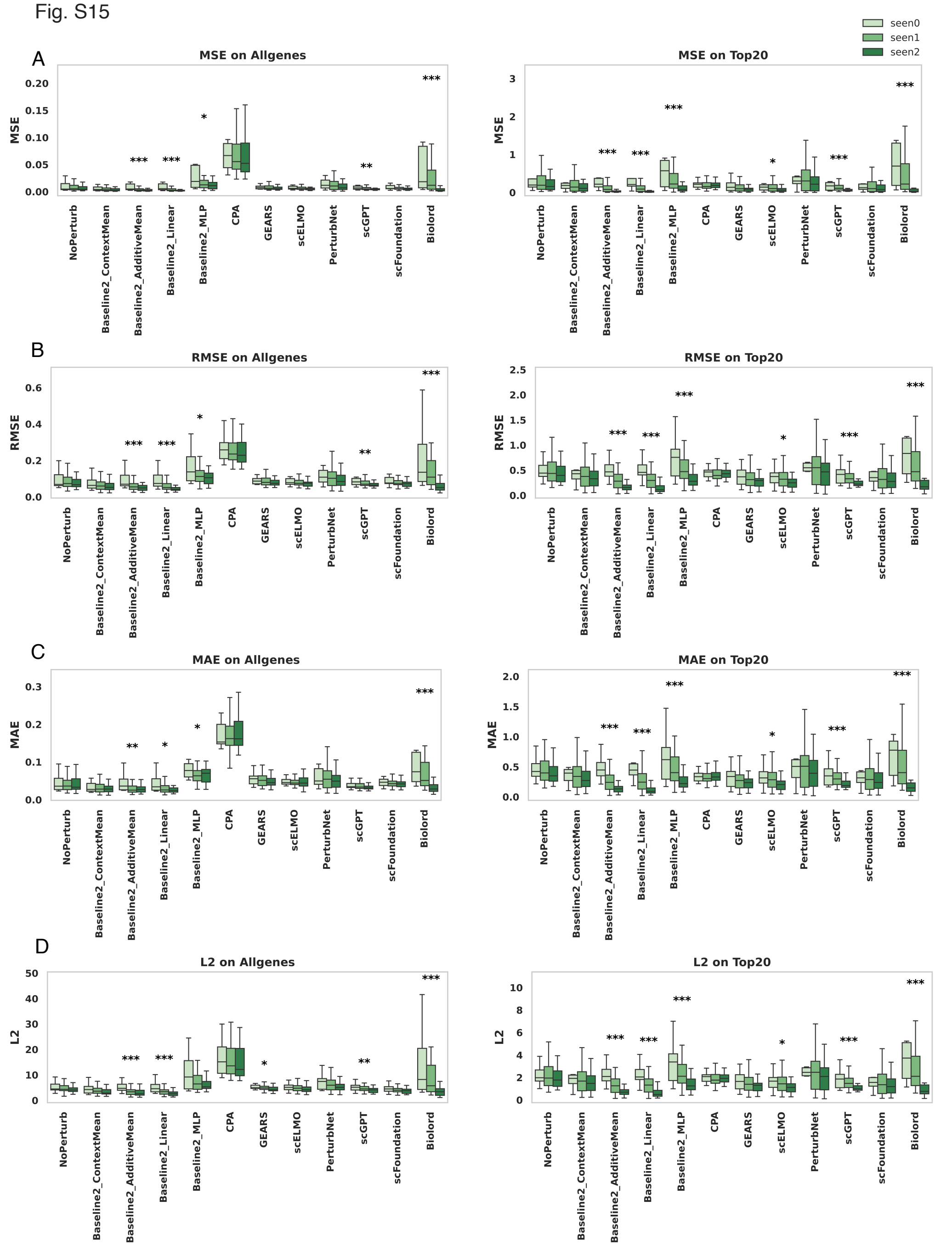

Fig. S15. Evaluation results of Task 2. Metrics aggregated across all datasets were computed for each model and seen level using all genes and the top 20 DEGs. Each model is plotted with seen0, seen1, and seen2. Four evaluation metrics are reported in this figure: (**A**) MSE, (**B**) RMSE, (**C**) MAE, and (**D**) L2 distance.

Fig. S16

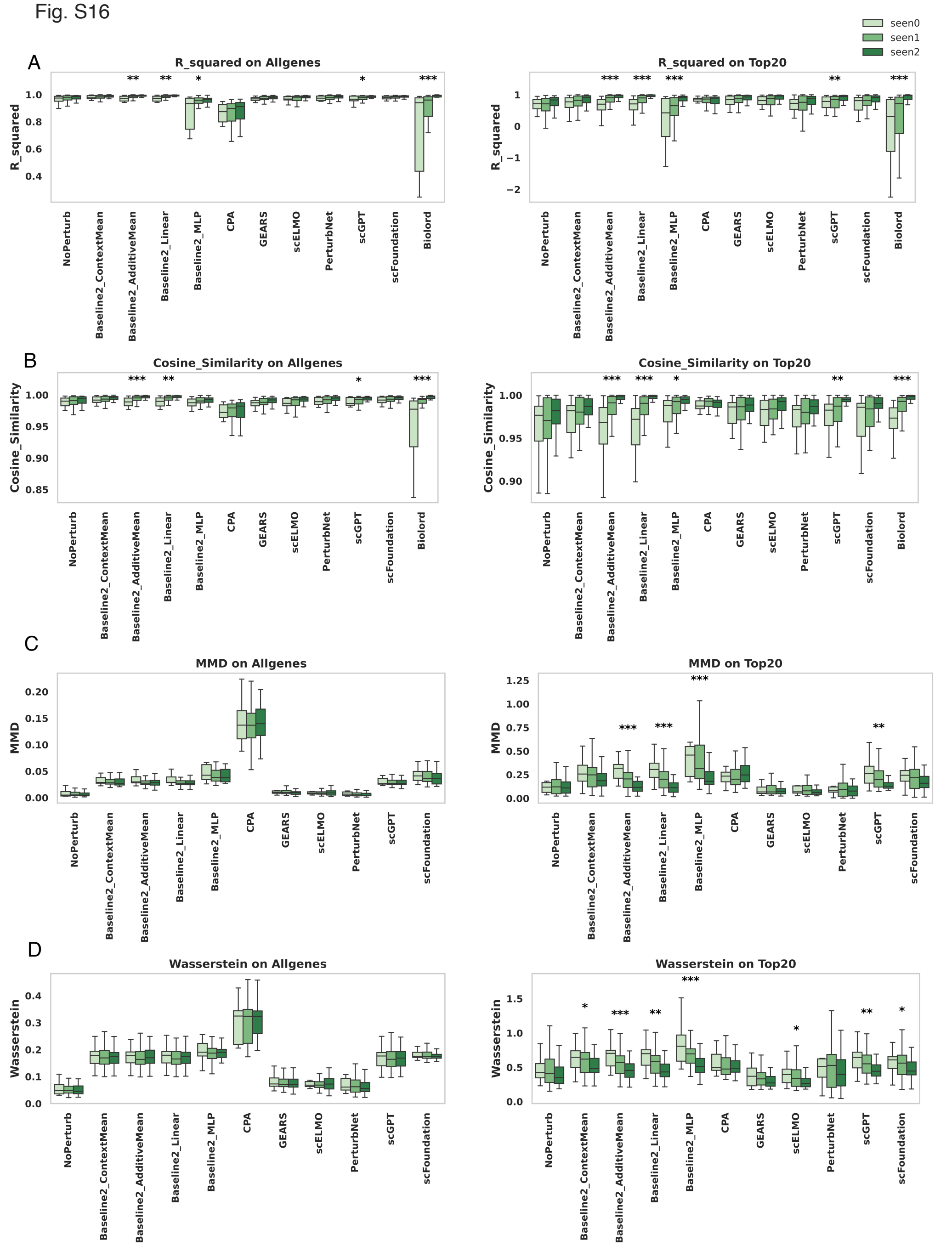

Fig. S16. Evaluation results of Task 2. Metrics aggregated across all datasets were computed for each model and seen level using all genes and the top 20 DEGs. Each model is plotted with seen0, seen1, and seen2. Four evaluation metrics are reported in this figure: (**A**) R^2, (**B**) Cosine similarity, (**C**) MMD, and (**D**) Wasserstein distance. (**E**) Pearson correlation and Pearson correlation delta calculated using the top 20 DEGs only.

Fig. S17

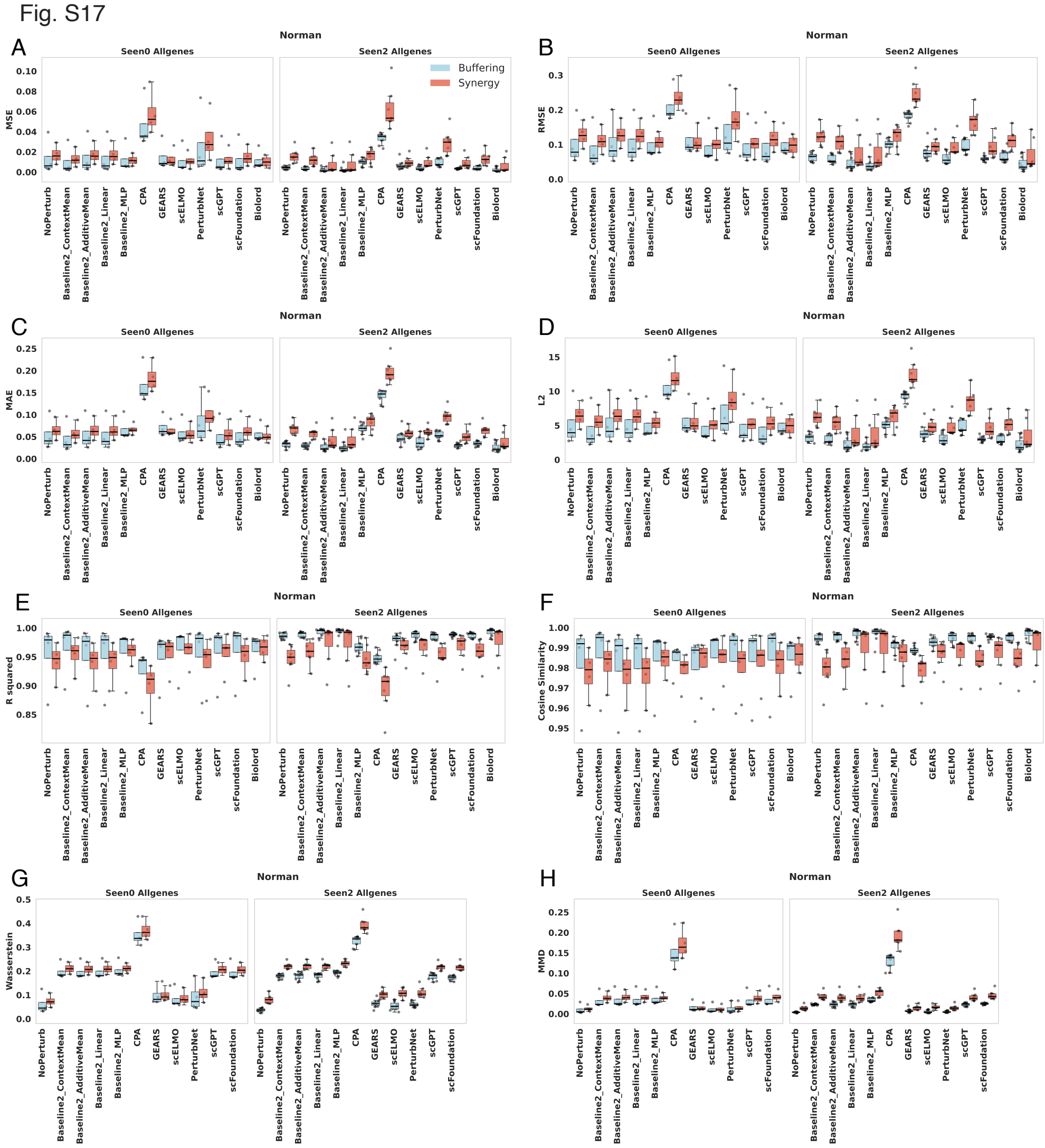

Fig. S17. Evaluation results of Task 2. Performance comparison between buffering and synergistic combinations for seen0 and seen2 conditions across different models on all genes of the Norman dataset. Metrics compared are **(A)** MSE, **(B)** RMSE, **(C)** MAE, **(D)** L2 distance, **(E)** R^2, **(F)** Cosine similarity, **(G)** Wasserstein distance, and **(H)** MMD.

Fig. S18

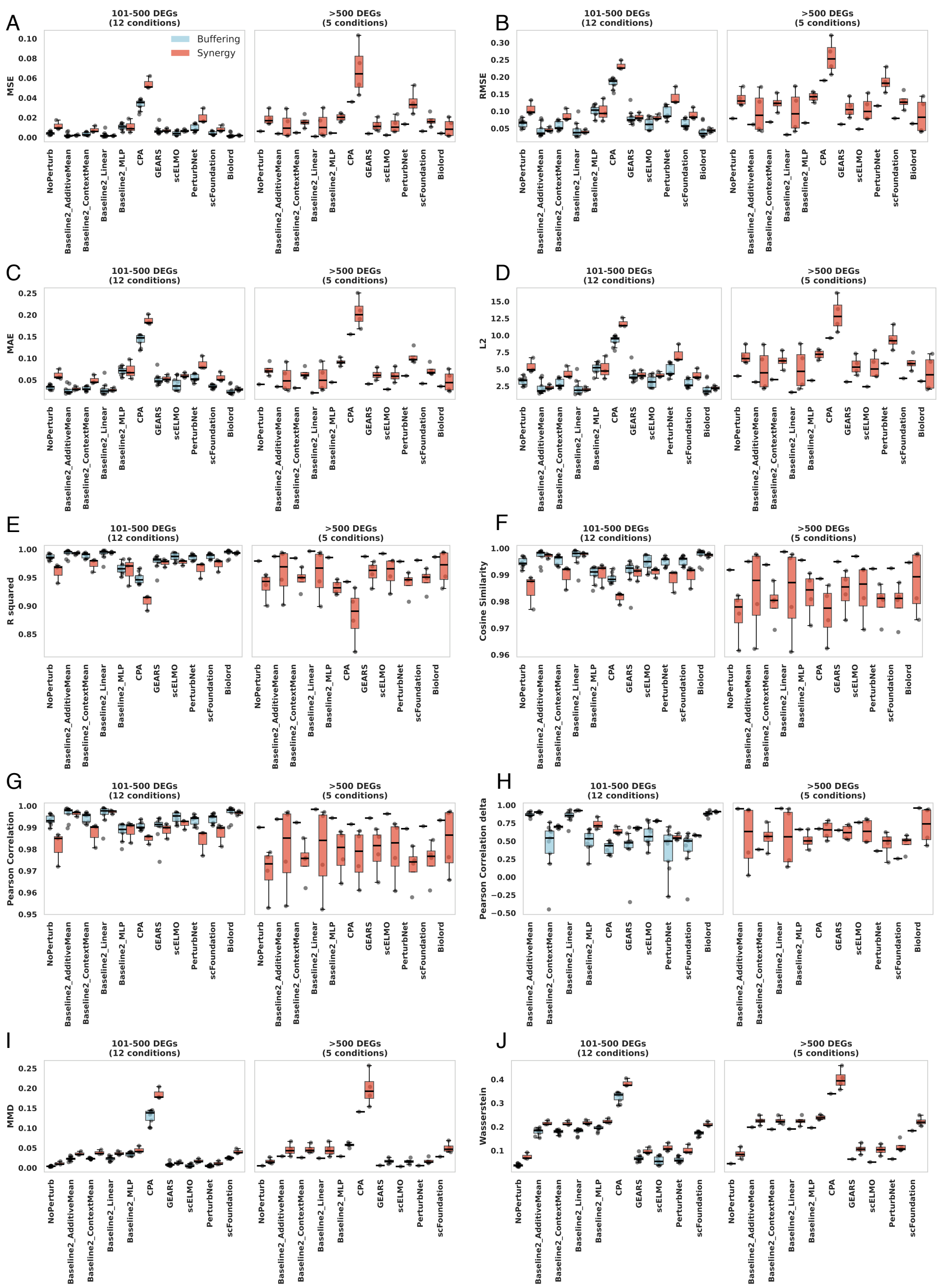

Fig. S18. Evaluation results of Task 2. Performance comparison between buffering and synergistic combinations for gene pairs grouped by perturbation effect size , including 101–500 DEGs and ¿500 DEGs, evaluated under seen0 test conditions across different models on all genes of the Norman dataset. Metrics compared are **(A)** MSE, **(B)** RMSE, **(C)** MAE, **(D)** L2 distance, **(E)** R^2, **(F)** Cosine similarity,**(G)** Pearson correlation, **(H)** Pearson correlation delta, **(I)** MMD, and **(J)** Wasserstein distance.

Fig. S19

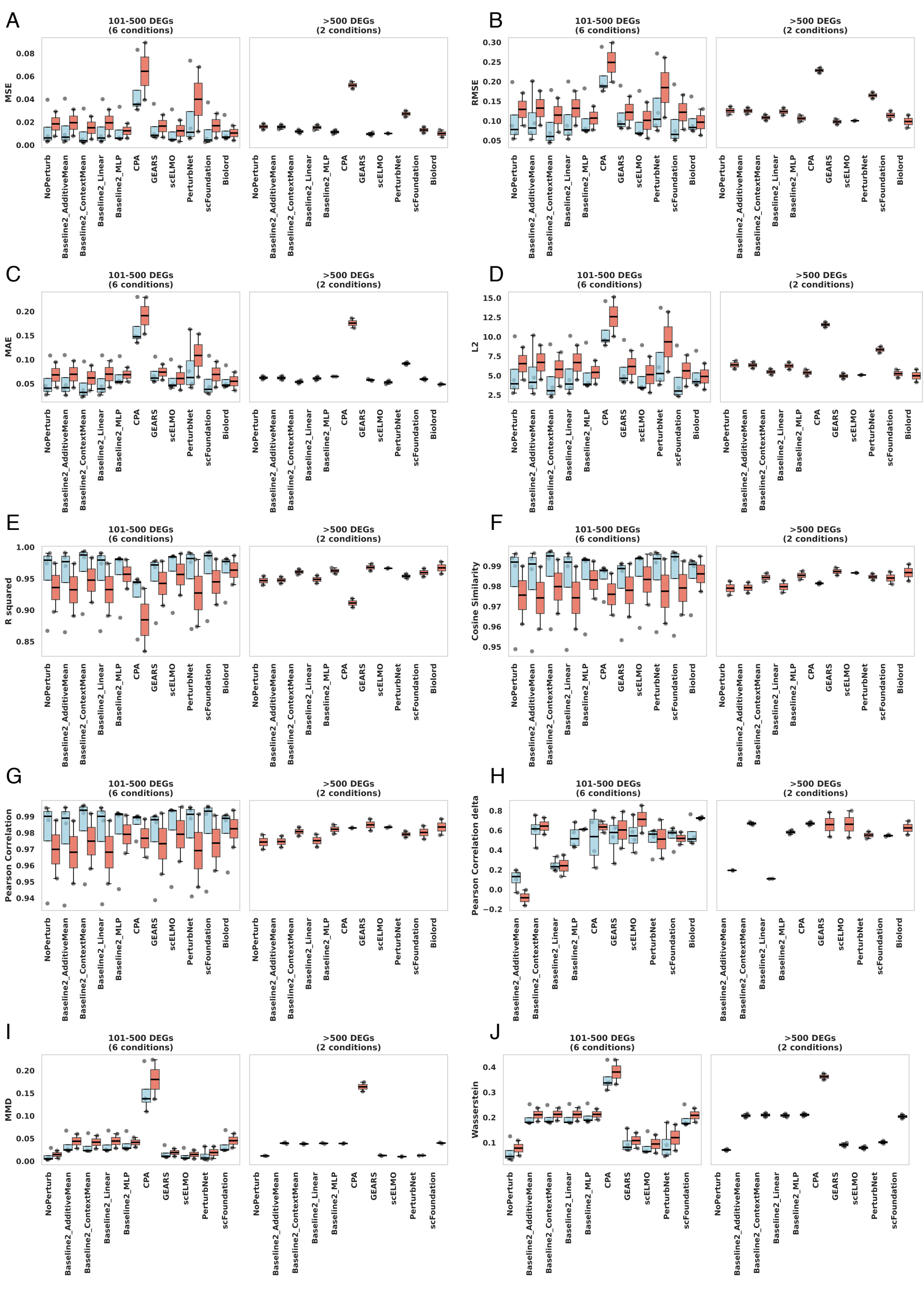

Fig. S19. Evaluation results of Task 2. Performance comparison between buffering and synergistic combinations for gene pairs grouped by perturbation effect size , including 101–500 DEGs and ¿500 DEGs, evaluated under seen0 test conditions across different models on all genes of the Norman dataset. Metrics compared are **(A)** MSE, **(B)** RMSE, **(C)** MAE, **(D)** L2 distance, **(E)** R^2, **(F)** Cosine similarity,**(G)** Pearson correlation, **(H)** Pearson correlation delta, **(I)** MMD, and **(J)** Wasserstein distance.

Fig. S20

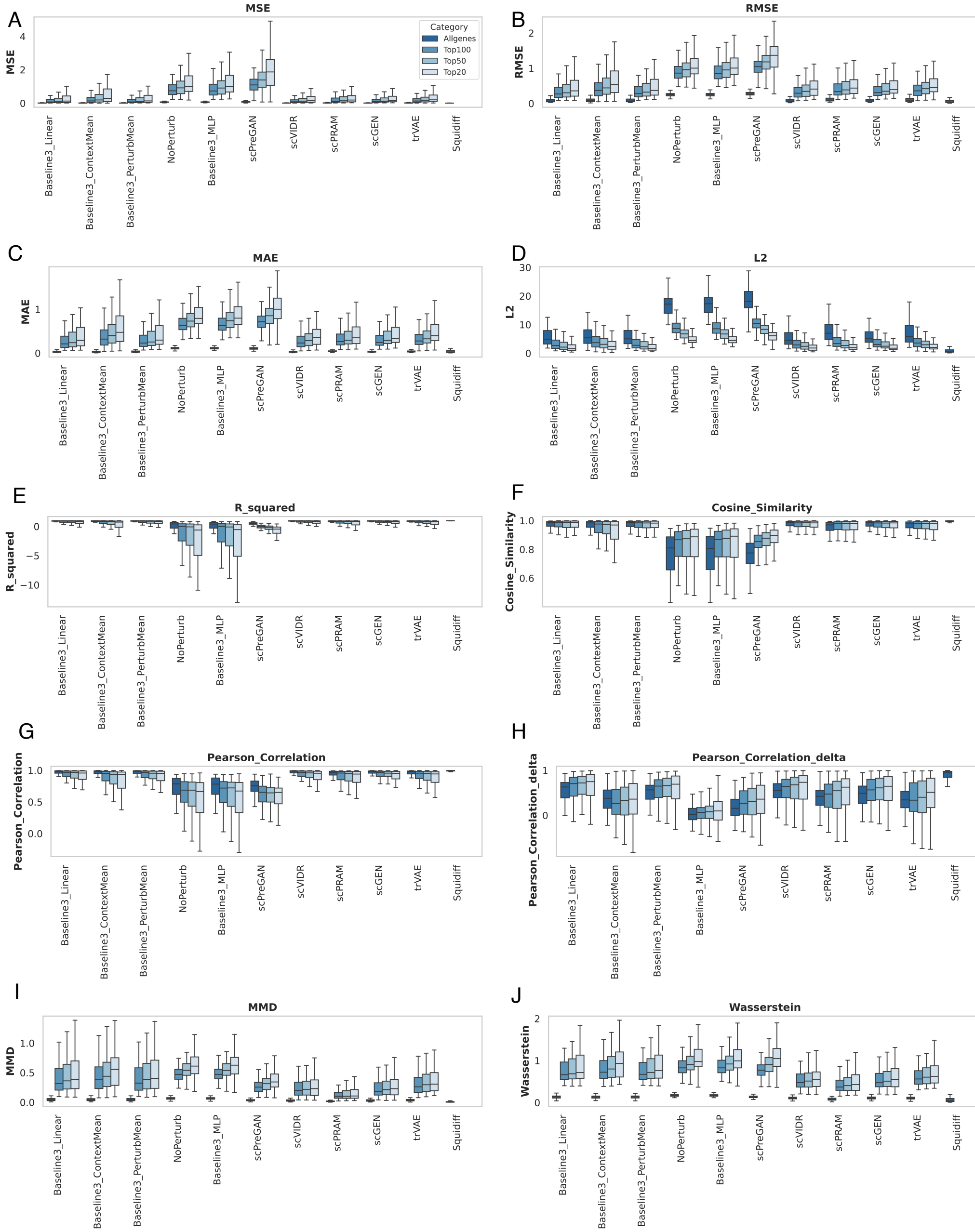

Fig. S20. Evaluation results of Task 3. Metrics aggregated on all datasets were computed across all models using four gene sets: all genes, top 100 DEGs, top 50 DEGs, and top 20 DEGs (ranked by absolute scores). Ten evaluation metrics are reported: (**A**) MSE, (**B**) RMSE, (**C**) MAE, (**D**) L2 distance, (**E**) R^2, (**F**) Cosine similarity, (**G**) Pearson correlation, (**H**) Pearson correlation delta, (**I**) MMD, and (**J**) Wasserstein distance.

Fig. S21

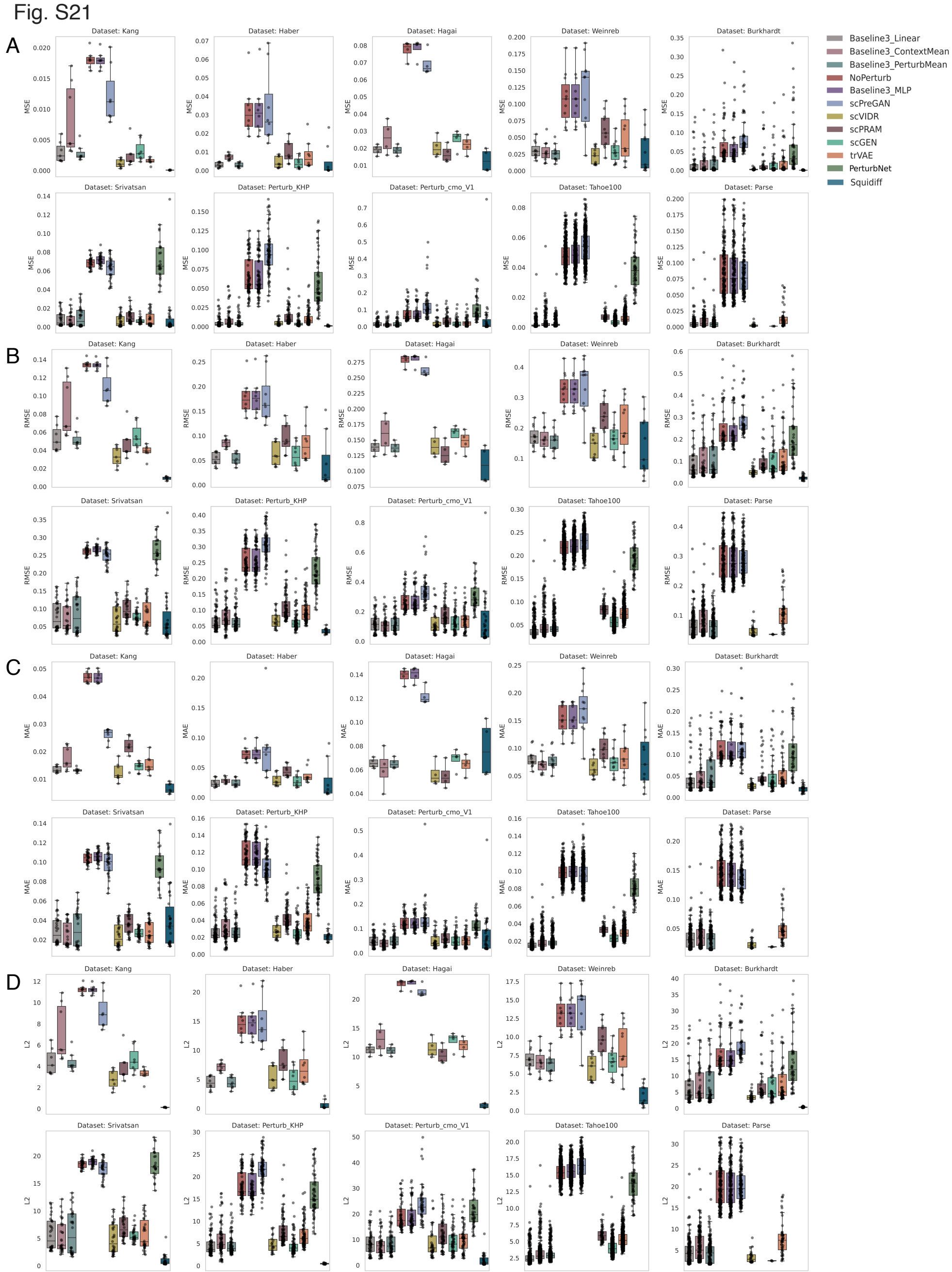

Fig. S21. Evaluation results of Task 3. Metrics were computed across all datasets and models using all genes. Each point represents a perturbation tested in the benchmark task. The evaluated datasets include Kang, Haber, Hagai, Weinreb, Burkhardt, Srivatsan, Perturb_KHP, Perturb_cmo_V1, Tahoe100, and Parse. Four evaluation metrics are reported on this figure: (**A**) MSE, (**B**) RMSE, (**C**) MAE, and (**D**) L2 distance.

Fig. S22

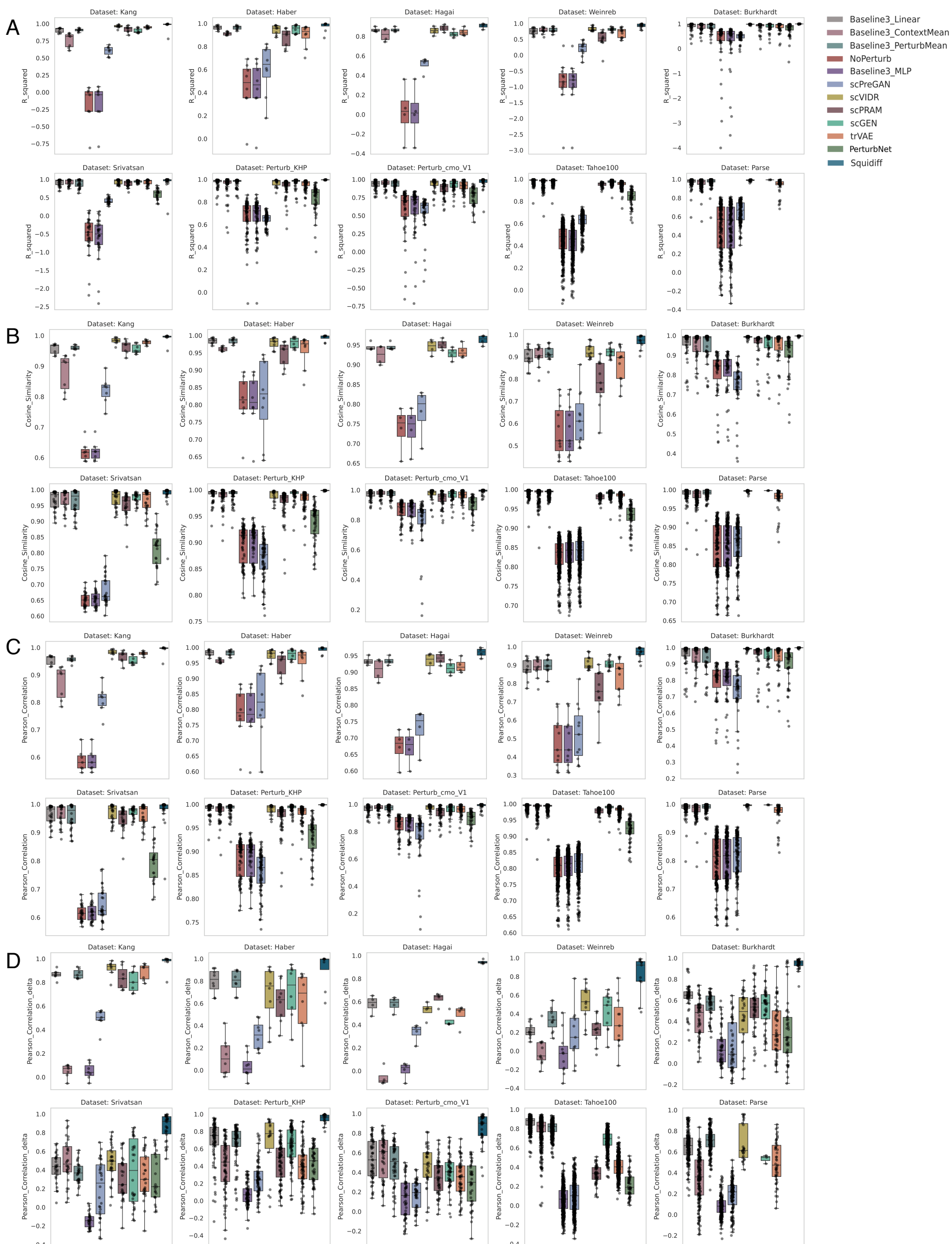

Fig. S22. Evaluation results of Task 3. Metrics were computed across all datasets and models using all genes. Each point represents a perturbation tested in the benchmark task. The evaluated datasets include Kang, Haber, Hagai, Weinreb, Burkhardt, Srivatsan, Perturb_KHP, Perturb_cmo_V1, Tahoe100, and Parse. Four evaluation metrics are reported on this figure: (A) R^2, **(B)** Cosine similarity, **(C)** Pearson correlation, and **(D)** Pearson correlation delta.

Fig. S23

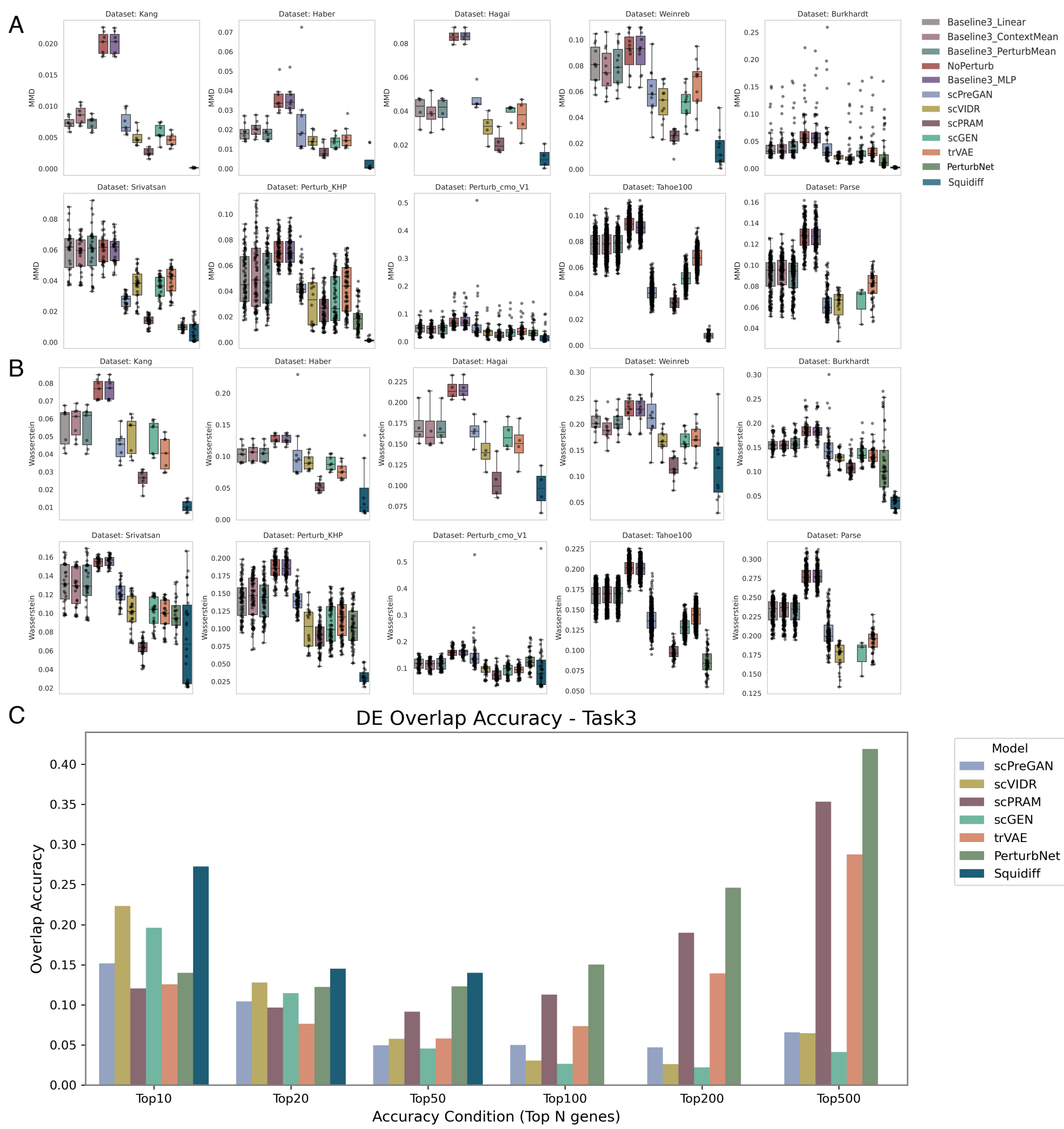

Fig. S23. Evaluation results of Task 3. **(A)** MMD and **(B)** Wasserstein distance across all datasets and models using all genes. Each point represents a perturbation tested in the benchmark task. Datasets include Kang, Haber, Hagai, Weinreb, Burkhardt, Srivatsan, Perturb_KHP, Perturb_cmo_V1, Tahoe100, and Parse. **(C)** Differentially expressed (DE) gene overlap accuracy between true and predicted perturbations versus control, aggregated on all datasets across all models.

Fig. S24

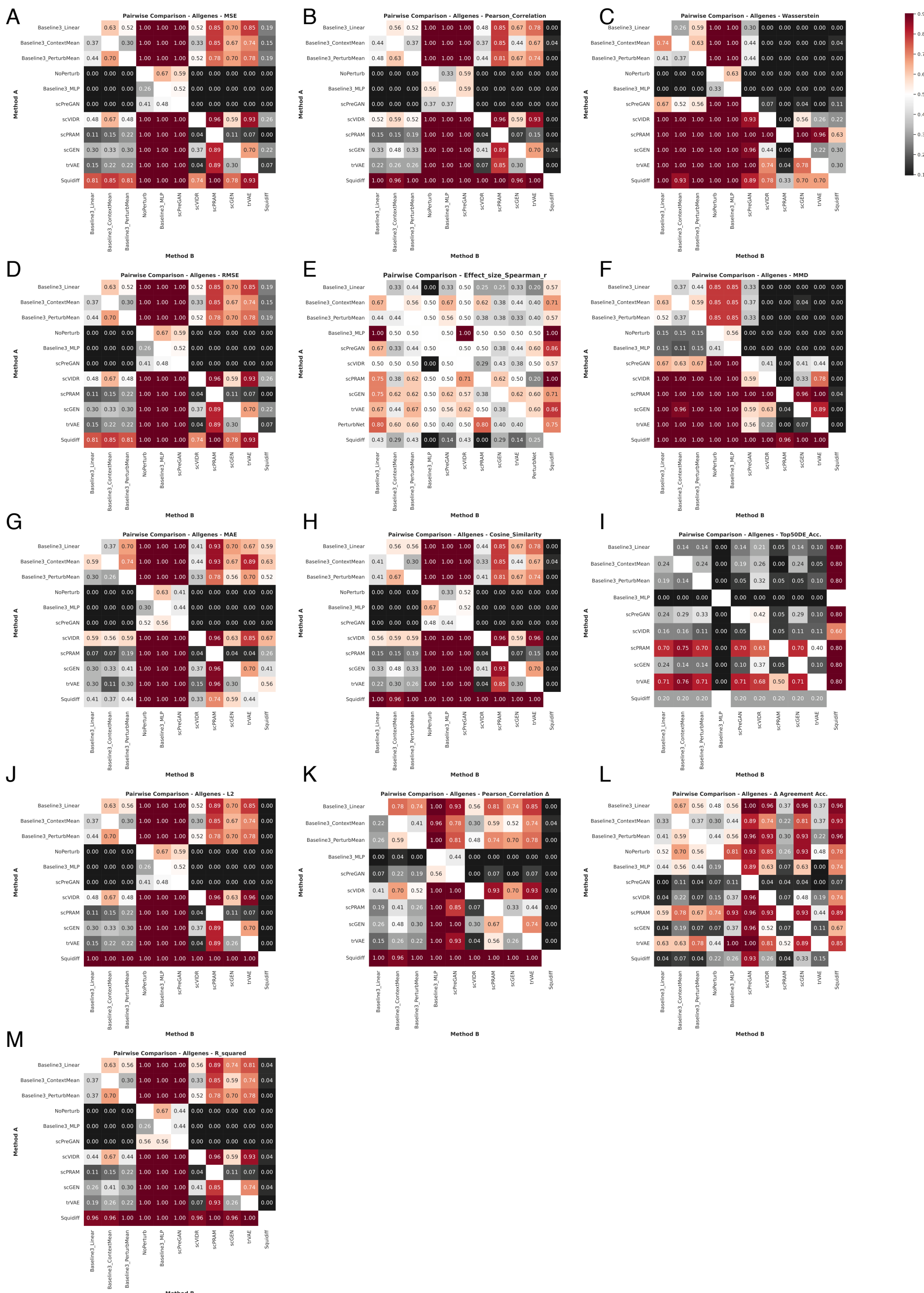

Fig. S24. Evaluation results of Task 3. (A-M) Pairwise outperformance heatmaps. Each panel corresponds to one evaluation metric: **(A)** MSE, **(B)** Pearson correlation, **(C)** Wasserstein distance, **(D)** RMSE, **(E)** Effect size correlation (Spearman r), **(F)** MMD, **(G)** MAE, **(H)** Cosine similarity, **(I)** Top 50 DE gene overlap accuracy, **(J)** L2 distance, **(K)** Pearson correlation delta, **(L)** delta direction agreement accuracy, and **(M)** R^2. Each cell (A, B) shows the fraction of dataset–perturbation conditions in which method A achieves a better rank than method B for the corresponding metric. Rankings are computed independently within each condition across all models, with ties handled using dense ranking.

Fig. S25

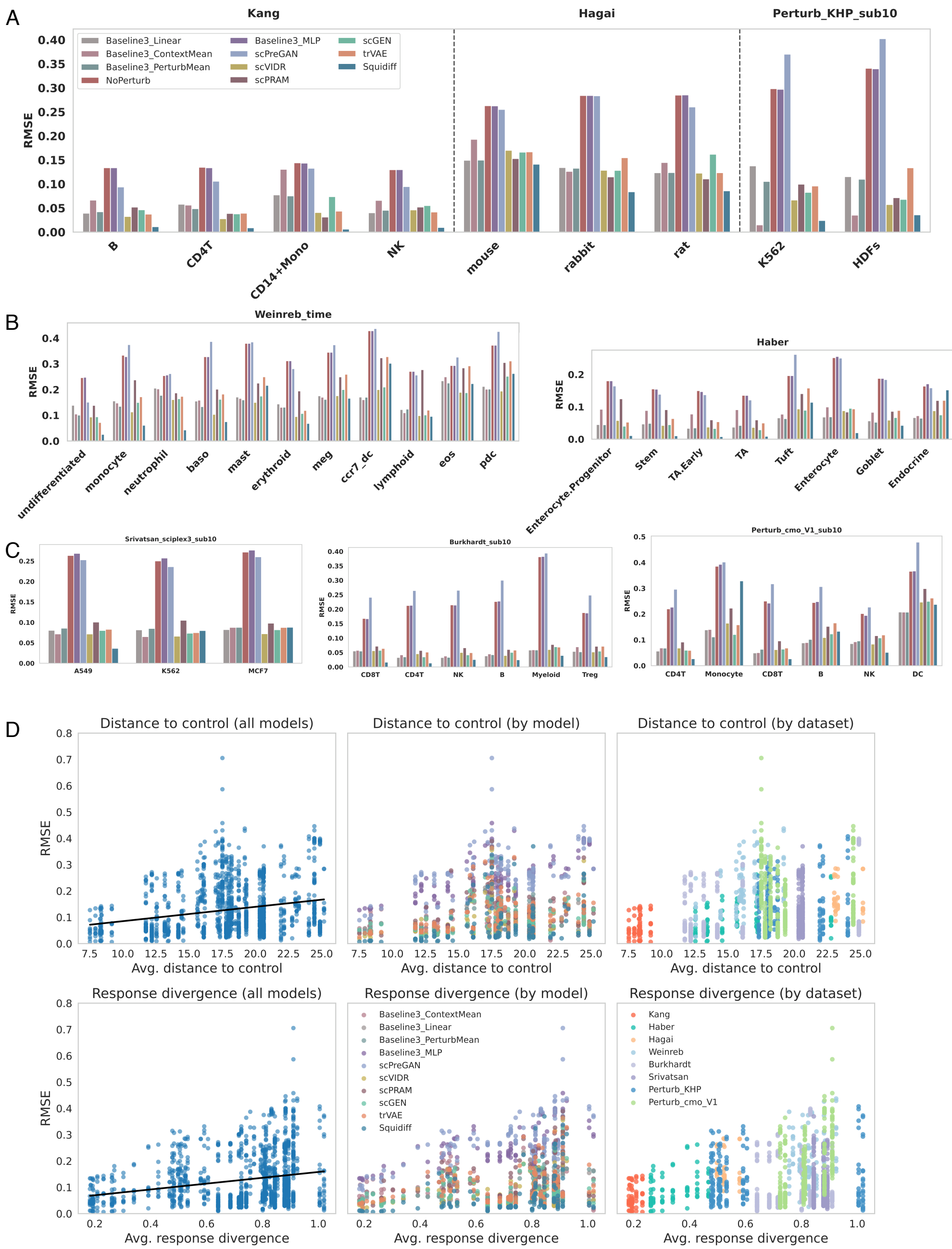

Fig. S25. Evaluation results of Task 3. (A-C) RMSE evaluation across all models and cell types of the eight datasets. **(D)** Relationship between cross-cell-type distances and Task 3 performance. RMSE versus average distance to control (top) and response divergence (bottom), shown for all models combined (left), by model (middle), and by dataset (right).

Fig. S26

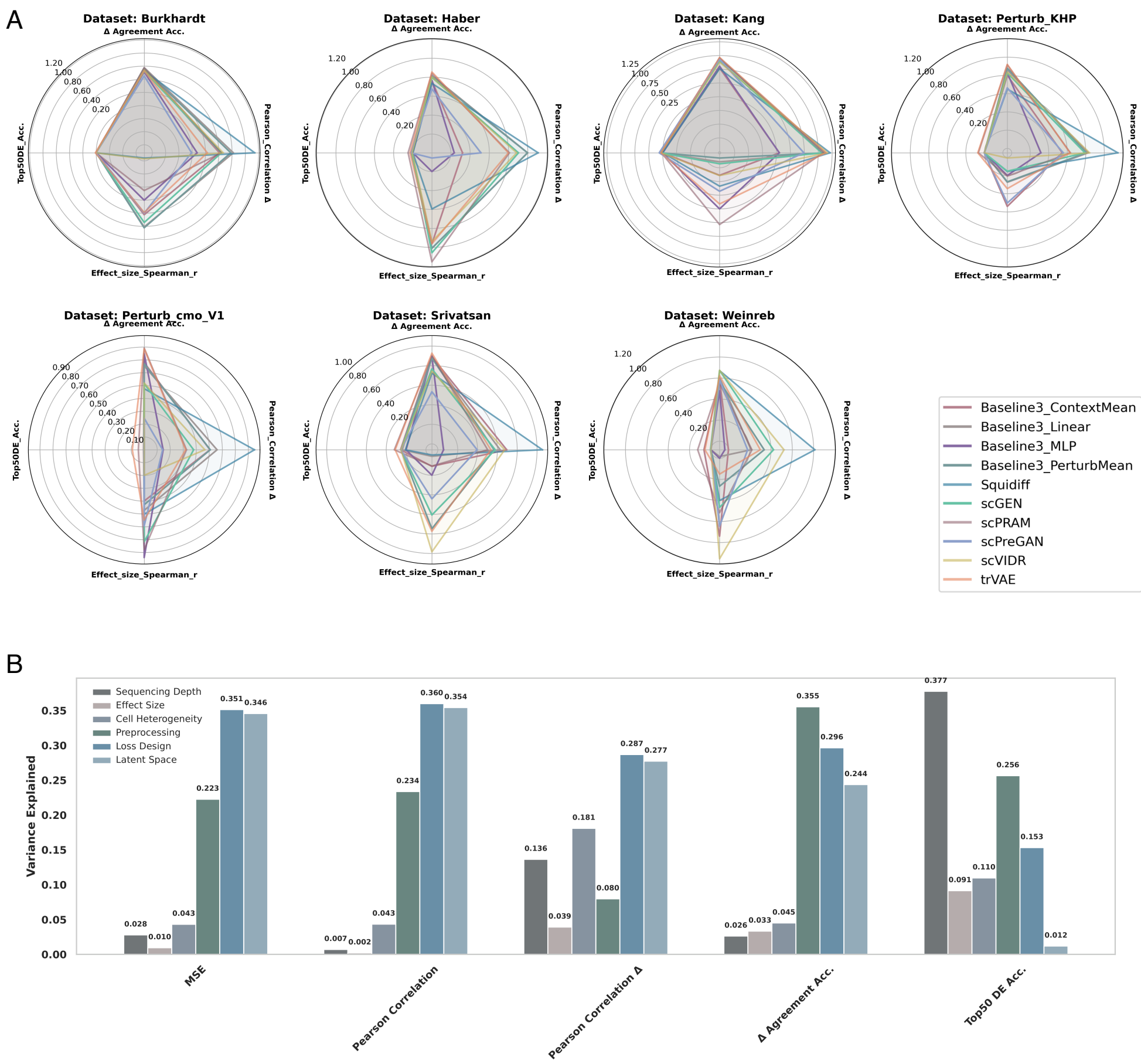

Fig. S26. Evaluation results of Task 3. (A) Top 50 DEG overlap accuracy, effect size correlation (Spearman r), Pearson's correlation coefficients on delta expressions, and delta direction accuracy across all tested models and datasets for cell state transfer prediction. **(B)** Variance decomposition analysis showing the relative contribution of dataset-level factors (sequencing depth, perturbation effect size, and cell heterogeneity) and model-level factors (preprocessing strategy, loss function design, and latent space architecture) to prediction performance across evaluation metrics.

Fig. S27

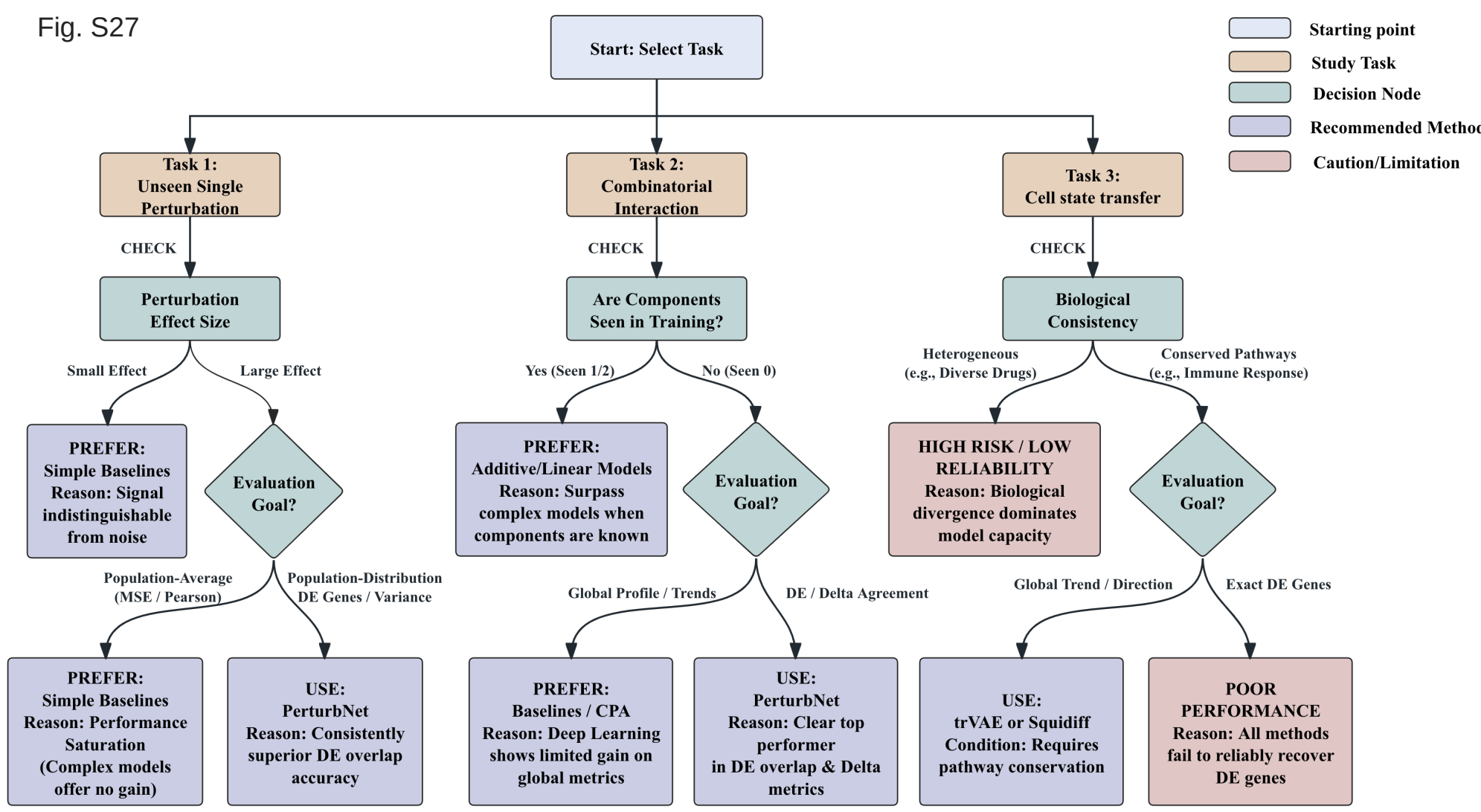

Fig. S27. Decision framework for perturbation preduction method selection.

Table S1.

Detailed descriptions of datasets used in this study.

Species	Task	Source paper	Data name	Cell type (cell line)	Download	Paper	Doi	DE count description	Intertwined factors
human	Task1	Datlinger	DatlingerBock2017.h5ad	Jurkat cells	https://zenodo.org/records/7041849	Pooled CRISPR screening with single-cell transcriptome readout	10.1038/nmeth.4177	Mostly 0 DEGs (≈95% of conditions show 0 DEGs)	Older tech; low sequencing depth; pooled screening; small cell numbers; stringent thresholds
human	Task1	Datlinger	DatlingerBock2021.h5ad	Jurkat cells	https://zenodo.org/records/7041849	Pooled CRISPR screening with single-cell transcriptome readout	10.1038/nmeth.4177	Mixed but many low-DE conditions (substantial fraction with 0 or 1-10 DEGs)	Improved over 2017 but still modest depth; small N per condition
human	Task1	Adamson	Adamson.h5ad	K562	https://dataverse.harvard.edu/api/access/datafile/6154417	A Multiplexed Single-Cell CRISPR Screening Platform Enables Systematic Dissection of the Unfolded Protein Response	10.1016/j.cell.2016.11.048	All perturbations show strong responses — >500 DEGs for every condition (user-corrected)	CRISPRi targeting UPR; strong biological effect size; high sequencing depth; many cells per perturbation
human	Task1	Joung	Fengzhang2023.h5ad	hESCs	From author	A transcription factor atlas of directed differentiation	10.1016/j.cell.2022.11.026	Large responses; many conditions with >500 DEGs	TF overexpression; modern tech; many cells
human	Task1	Frangieh	FrangiehIzar2021_RNA.h5ad	Melanoma cells	https://zenodo.org/records/7041849	Multimodal pooled Perturb-CITE-seq screens in patient models define mechanisms of cancer immune evasion	10.1038/s41588-021-00779-1	Many subtle responses; high heterogeneity across patient models	Patient-derived models; multimodal readout; heterogeneity reduces RNA-only signal
human	Task1	Papalexi	PapalexiSatija2021_eccite_RNA.h5ad	THP-1	https://zenodo.org/records/7041849	Characterizing the molecular regulation of inhibitory immune checkpoints with multimodal single-cell screens	10.1038/s41588-021-00778-2	Small-to-moderate; mixed distribution with some strong hits	Multimodal eCCITE-seq; immune context; variable effect sizes
human	Task1	Replogle	ReplogleWeissman2022_rpe1.	RPE1	https://zenodo.org/records/7041849	Mapping information-rich genotype-phenotype landscapes with	10.1016/j.cell.2022.05.013	Many moderate-to-large responses; notable fraction >500 DEGs	Genome-scale CRISPRi; RPE1 cells; high coverage and good experimental design

			h5ad			genome-scale Perturb-seq			
human	Task1	Tian	TianKampmann2021_CRISPRi.h5ad	iPSC-induced neuron	https://zenodo.org/records/7041849	Genome-wide CRISPRi/a screens in human neurons link lysosomal failure to ferroptosis	10.1038/s41593-021-00862-0	Mostly low — many conditions in 0 – 10 DEGs	Differentiated neurons; low transcriptional plasticity; CRISPRi efficiency variable
human	Task1	Tian	TianKampmann2021_CRISPRa.h5ad	iPSC-induced neuron	https://zenodo.org/records/7041849	Genome-wide CRISPRi/a screens in human neurons link lysosomal failure to ferroptosis	10.1038/s41593-021-00862-0	Many near−zero responses	CRISPRa in neurons may be constrained; limited effect size
human	Task1	Xu	Junyue_Cao.h5ad	A375	From author	Uncovering Convergent Cell State Dynamics Across Divergent Genetic Perturbations Through Single-Cell High-Content CRISPR Screening	https://doi.org/10.1101/2025.05.02.651939	Mostly moderate−to−large responses (many 101−500)	High−content CRISPRi; large effects for some perturbations; good protocols
human	Task1	Virtual Cell Challenge	vcc_train_data.h5ad	hESCs	https://virtualcellchallenge.org/app/datasets	-	-	−	−
human	Task1/2	Dixit	DixitRegev2016.h5ad	K562	https://zenodo.org/records/7041849	Perturb-seq: dissecting molecular circuits with scalable single-cell RNA profiling of pooled genetic screens.	10.1016/j.cell.2016.11.038	Many moderate responses (11 – 500)	Early perturb−seq; solid design but older tech
human	Task1/2	Norman	NormanWeissman2019_filtered.h5ad	K562	https://dataverse.harvard.edu/api/access/datafile/6154020	Exploring genetic interaction manifolds constructed from rich single-cell phenotypes	10.1126/science.aax4438	Majority moderate−to−large	CRISPRa; rich phenotypes; high−quality design
human	Task1/2	Sunshine	Sunshine2023_CRISPRi_sarscov2.h5ad	Calu-3 cells	https://www.ncbi.nlm.nih.gov/geo/query/acc.cg	Systematic functional interrogation of SARS-CoV-2 host factors using	10.1038/s41467-023-41788-4	Very large responses; majority >500 DEGs	Viral host factor perturbations; highly responsive cells; modern Perturb−seq

					i?acc=GSE208240	Perturb-seq			
human	Task1/2	Maya M. Arce	Arce_MM_CRISPRi.h5ad	CD4+ T cells	GSE278572	Central control of dynamic gene circuits governs T cell rest and activation	10.1038/s41586-024-08314-y	Mostly low-to-moderate (1–100 DEGs) with few large hits	T cell context; immune activation states; moderate depth
human	Task3	Kang	Kang.h5ad	B, CD14+Mono, CD4T, CD8T, Dendritic, FCGR3A+Mono, NK	https://github.com/theislab/scgen-reproducibility/blob/master/code/DataDownloader.py	Multiplexed droplet single-cell RNA-sequencing using natural genetic variation	10.1038/nbt.4042	Small sample size and mixed responses; some large responses but n small	IFN-β stimulation in PBMCs; highly responsive stimulus but limited conditions
mouse	Task3	Haber	Haber.h5ad	Endocrine, Enterocyte, Enterocyte.Progenitor, Goblet, Stem, TA, TA.Early, Tuft	https://www.dropbox.com/s/7ngt0hv21hl2exn/train_hpoly.h5ad?dl=1	A single-cell survey of the small intestinal epithelium	10.1038/nature24489	Small dataset; many moderate responses (101–500 bins)	Developmental context; tissue-specific responses; small n
mouse	Task3	Weinreb	Weinreb.h5ad	baso, ccr7_dc, eos, erythroid, lymphoid, mast, meg, monocyte, neutrophil, b, pdc, undifferentiated	https://www.research-collection.ethz.ch/handle/20.500.11850/609681	Lineage tracing on transcriptional landscapes links state to fate during differentiation	10.1126/science.aaw3381	Mixed across bins; time-dependent signals	Differentiation timecourses; lineage tracing; dynamic signals
cross-species	Task3	Hagai	Hagai.h5ad	mouse, pig, rabbit, rat	https://www.dropbox.com/s/eprgwhd98c9quiq/train_species.h5ad?dl=1	Gene expression variability across cells and species shapes innate immunity	10.1038/s41586-018-0657-2	Very large responses for all conditions (small n; all >500)	LPS innate immune stimulation; cross-species comparisons; highly responsive

human	Task3	Srivatsan	Srivatsan_sciplex3_sub10.h5ad	MCF7, A549, K562	https://zenodo.org/records/7041849	Massively multiplex chemical transcriptomics at single-cell resolution	10.1126/science.aax6234	Mixed but many moderate responses (11 – 500 range)	Chemical perturbations; dose/target-dependent variability; multiplexing
human	Task3	Burkhardt	Burkhardt_sub10.h5ad	CD4T, CD8T, Treg, B, myeloid, NK	https://www.kaggle.com/competitions/open-problems-single-cell-perturbations/data	Single-cell perturbation prediction: generalizing experimental interventions to unseen contexts. In Single-cell perturbation prediction competition.	-	Mixed distribution across bins; heterogeneous responses per compound	Drug screens; variable mechanisms/doses; competition benchmark
human	Task3	Tahoe	Tahoe100_sub10.h5ad	CVCL_1125, CVCL_0292, CVCL_1693, CVCL_0099, CVCL_1717, CVCL_1055, CVCL_1550, CVCL_1097, CVCL_0334, CVCL_1056, CVCL_1098, CVCL_1635, CVCL_0366, CVCL_0504, CVCL_1239, CVCL_0131, CVCL_1285, CVCL_0332, CVCL_1724, CVCL_1094, CVCL_0397, CVCL_0320, CVCL_0152, CVCL_1478, CVCL_0399, CVCL_0218, CVCL_1495, CVCL_0428, CVCL_1517,	https://github.com/ArcInstitute/arc-virtual-cell-atlas/tree/main	Tahoe-100M: A Giga-Scale Single-Cell Perturbation Atlas for Context-Dependent Gene Function and Cellular Modeling	https://doi.org/10.1101/2025.02.20.639398	Mostly 11 – 100 DEGs (≈ 70% in 11 – 100 bin), few >500	Large-scale drug atlas; many contexts; many moderate responses

				CVCL_0480, CVCL_C466, CVCL_0028, CVCL_1381, CVCL_0546, CVCL_0459, CVCL_0179, CVCL_0069, CVCL_0359, CVCL_1731, CVCL_0293, CVCL_0371, CVCL_0023, CVCL_1547, CVCL_1666, CVCL_1119					
human	Task3	Parse Biosciences	Parse_10M_PBMC_cytokines.h5ad	B Intermediate/ Memory, B Naive, CD14 Mono, CD16 Mono, CD4 Memory, CD4 Naive, CD8 Memory, CD8 Naive, HSPC, ILC, MAIT, NK, NK CD56bright, NKT, Plasmablast, Treg, cDC, pDC	https://parse-wget.s3.us-west-2.amazonaws.com/10m/Parse_10M_PBMC_cytokines.h5ad	Parse Biosciences. 10 million human pbmcs in a single experiment, 2023.	-	–	–
human	Task3	This study	Perturb_KHP_sub10.h5ad	K562, HDFs, CD4T, CD8T, B, DC, Mast, Monocyte, Macrophage, Erythrocytes	-	This paper	-	Many moderate-to-large responses; substantial fraction in 101 – 500 and >500 bins (~80% combined)	Drug treatments in PBMC-like contexts; dose/target dependent; decent coverage
human	Task3	This study	Perturb_cmo_V1_sub10.h5ad	CD4T, CD8T, B, DC, Monocyte, NK	-	This paper	-	Mixed — many low responses but a subset with moderate-to-large DE counts (~10% >500)	Drug panel; early library; variable dosing/targets; modest depth

Table S2.

Overview of perturbation-related models.

Name	Year	Whether used in this study	Research tasks	Expression data embedding method	Perturbation data embedding method	Model architecture	Published journal or conference	Title	DOI	Baseline model or method
Rachel et al	2018	No	Predicting cell-specific drug perturbation profiles using available expression data from related conditions（other drugs and cell types）to fill gaps remain in this combinatorial drug-cell space.	Arranges existing profiles into a three-dimensional array (or tensor) indexed by drugs, genes, and cell types	Uses either local (nearest-neighbors) or global (tensor completion) information to predict unmeasured profiles	Arranges existing profiles into a three-dimensional array (or tensor) indexed by drugs, genes, and cell types, and then uses either local (nearest-neighbors) or global (tensor completion) information to predict unmeasured profiles	Pacific Symposium on Biocomputing 2018	Cell-specific prediction and application of drug-induced gene expression profiles	10.1142/9789813235533_0004	CPA is compared with existing methods using cross-validation for gene expression prediction and DEGs, focusing on input data volume and cell-specificity.
DTD	2019	No	Infer potential drug-target-disease interactions for rational drug repositioning	Three-way drug × target × disease tensor *X* of size n1 × n2 × n3, in which n1, n2, and n3 denote the number of drugs, targets, and diseases, respectively. where each entry in the tensor represents the interaction between drugs, targets, and diseases, and is derived from online data sources describing these entities.	Perturbation information is embedded by incorporating multiple auxiliary information sources of drugs, targets, and diseases into the model. This includes chemical structures, side effects, protein sequences, and phenotype descriptions obtained from various online databases, which are then used to form similarity or affinity matrices that are integrated into the tensor factorization process.	The DTD model structure involves a coupled tensor-matrix factorization that leverages Tucker Decomposition to learn a third-order drug × target × disease tensor. It also includes the incorporation of multiple auxiliary feature matrices coupled in the "drug", "target", and "disease" modes, respectively, and optimizes the factor matrices and the core tensor on the Grassmann manifold.	The World Wide Web Conference, 2019	Modeling Relational Drug-Target-Disease Interactions via Tensor Factorization with Multiple Web Sources	10.1145/3308558.3313476	no
CPA	2023	Yes	Predict scRNA-seq perturbation responses across combinations of conditions such as dosage, time, drug,	Encodes gene expression using a neural network into a lower dimensional latent space that is	Given a scRNA-seq dataset, the perturbations applied, and covariates describing the	The CPA model comprises an encoder neural network that learns additive embeddings for a cell's basal state, perturbations, and covariates,	Molecular Systems Biology	Predicting cellular responses to complex perturbations	10.15252/msb.202211517	Two baseline models validate the CPA model: random baseline compares gene

			and genetic knock-out	eventually decoded back to an approximate gene expression matrix, as close as possible to the original one.	experimental setting, CPA decomposes the data into a collection of embeddings (representations) associated with the cell type, perturbation, and other external covariates.	which are disentangled through a discriminator classifier. These embeddings are then linearly integrated and decoded using a non-linear neural network to predict gene expression, allowing the model to capture complex, non-additive effects of perturbation combinations.		in high-throughput screens		expression means, and linear baseline evaluates combinatorial perturbation predictions.
CellBox	2021	No	Predicts cellular responses to perturbations, including unseen combinations of drugs, by modeling the dynamic behavior of cellular systems through differential equations.	The model uses a set of ordinary differential equations (ODEs) to represent interactions between proteins and phenotypic variables, which are learned from perturbation data to accurately simulate the dynamic cellular responses.	Perturbation information is embedded in the model by linking perturbations, such as drug treatments, to changes in protein levels and cellular phenotypes through interaction parameters in the ODEs, which are optimized to fit experimental data.	The CellBox model structure consists of a dynamic network of interactions between molecular and phenotypic variables, represented by ODEs. The parameters of these equations are trained using perturbation data to predict the system's response to both observed and unobserved conditions.	Cell systems	CellBox: Interpretable Machine Learning for Perturbation Biology with Application to the Design of Cancer Combination Therapy	10.1016/j.cels.2020.11.013	CellBox is compared with a random model to predict drug response, focusing on test splits and OOD data.
CellDrift	2022	No	Identifying covarying temporal patterns of various cell types in response to perturbations	Utilizing GLM with a negative binomial distribution to model the raw count of single-cell data for each gene and cell	Perturbation information is embedded through the inclusion of cell-type and perturbation group indicators in the GLM as covariates. The model uses one-hot matrices to represent whether a cell belongs to a specific cell type or perturbation group, and an interaction term is added to account for distinct responses of different cell types to the same perturbation.	The model structure of CellDrift involves a GLM-based approach that first captures the main effects and interaction effects of cell types and perturbations. It then uses the derived contrast coefficients as input for FDA methods, such as functional principal component analysis (FPCA) and fuzzy C-means clustering, to identify temporal patterns in the perturbation responses.	Briefings in Bioinformatics	CellDrift: inferring perturbation responses in temporally sampled single-cell data	10.1093/bib/bbac324	CellDrift is compared with traditional statistical methods like Wilcoxon and t-tests, addressing batch effects and data sparsity.

MultiCPA	2022	No	Predict multimodal perturbation responses at the single-cell level, encodes and learns phenotypic drug response across different cell types, doses and drug combinations.	Constructing joint feature vectors from the observed data of both modalities, proteins (xP) and genes (xG). In the concatenation-based model, these feature vectors are concatenated to form a joint embedding, Zjoint, which is sampled from the posterior distribution q(Zjoint|xG, xP) estimated by a shared encoder.	Using adversarial networks that take the joint embedding Zjoint as input and are trained to predict the perturbations and cell covariates. This process forces the encoder to produce a basal cellular state, Zbasal, which captures the effects of the perturbations. The perturbation and covariate embeddings are then combined with Zbasal in the latent space for decoding and prediction tasks.	The model structure of MultiCPA consists of an encoder-decoder framework with variational autoencoders (VAEs) and adversarial networks. It includes modality-specific decoders that attempt to reconstruct the corresponding input data. The model has two alternative mixture models for integrating multimodal perturbation profiles: concatenation and PoE, with the concatenation model outperforming the PoE model in predicting protein data. The model also includes a loss function that combines gene and protein data reconstruction losses, KL divergence terms, and adversarial losses for training.	ICML CompBio 2023	MultiCPA: Multimodal Compositional Perturbation Autoencoder	10.1101/2022.07.08.499049	Random model behavior is used to validate MultiCPA in predicting multimodal perturbation responses, with evaluation using Rsquared and FID metrics.
PerturbNet	2022	Yes	Predict the distribution of cell states induced by unseen chemical or genetic perturbations	Training variational autoencoders (VAEs) on large expression datasets, these VAEs encode the gene expression data into latent representations that capture the underlying structure of the cell states.	For chemical perturbations, it uses a ChemicalVAE that encodes molecular structures into low-dimensional vectors based on their SMILES strings. For genetic perturbations, it uses a GenotypeVAE that represents each target gene as a one-hot vector of gene ontology annotations, and for coding sequence mutations, it uses a pre-trained ESM network to obtain latent representations of	The PerturbNet framework consists of three neural networks: a perturbation representation network, a cellular representation network, and a conditional invertible neural network (cINN) that maps from perturbations to cell states. The perturbation and cell representation networks are trained separately to encode perturbations and cell profiles into latent spaces, and the cINN learns the mapping between these spaces using high-throughput perturbation response data.	bioRxiv	PerturbNet predicts single-cell responses to unseen chemical and genetic perturbations	10.1101/2022.07.20.500854	PerturbNet is compared with a random model for predicting chemical and genetic perturbations, evaluated on Sci-Plex and LINCS-Drug datasets.

	2022				protein sequences.					
scINSIGHT	2022	No	This study develops scINSIGHT to interpret single-cell gene expression by decomposing data with NMF.	scINSIGHT embeds the expression data by representing each gene module as a non-negative linear combination of a subset of coordinated genes. It uses a matrix factorization approach to decompose the gene expression matrices into common and condition-specific components, allowing for the joint analysis of multiple single-cell samples.		The model structure of scINSIGHT is based on non-negative matrix factorization (NMF) and includes an objective function that minimizes the factorization error with constraints on the scale of condition-specific components and similarity between them. It consists of expression matrices for condition-specific and common gene modules, membership matrices for genes within these modules, and a residual matrix for each sample to account for technical noise.	Genome biology	scINSIGHT for interpreting single-cell gene expression from biologically heterogeneous data	10.1186/s13059-022-02649-3	scINSIGHT is compared with baseline models for modeling heterogeneous scRNA-seq datasets, improving predictive accuracy.
scPreGAN	2022	Yes	Predicting the response of single-cell expression to perturbation	The model embeds expression data through an encoder network, which maps the input of both unperturbed and perturbed data to a common low-dimensional latent space, extracting the common features of both datasets.	Perturbation information is embedded in scPreGAN by using the latent vectors from the encoder to feed into two generator networks, G1 and G2, which generate data corresponding to the unperturbed and the perturbed conditions, respectively. The predicted perturbed data are the output of neural networks, which do not rely on conditional variables associated with	The model structure of scPreGAN consists of an encoder network, two generator networks (G1 and G2), and two discriminator networks (D1 and D2). The encoder extracts common information from the data, and the generators reconstruct the unperturbed data and predict the corresponding perturbed data from the common latent vectors. The discriminators train against the generators to make the generated distribution more realistic.	Bioinformatics	scPreGAN, a deep generative model for predicting the response of single-cell expression to perturbation	10.1093/bioinformatics/btac357	scGen, trVAE, and stVAE are compared with scPreGAN for out-of-sample predictions, assessing which model predicts gene expression responses best.

					specific perturbations.					
cycleCDR	2024	No	Utilizing cycle consistency learning for the interpretable modeling of cellular responses to drug perturbations	The model uses an encoder-decoder architecture where the encoder maps the input cellular states (expression data) into a low-dimensional latent space, creating a representation that captures the essential features of the cellular states.	The model employs a graph attention network (GAT) to encode the drug perturbation into a latent space representation, where the molecular structure of the drug is represented as a graph, with nodes as atoms and edges as chemical bonds. The GAT calculates attention scores to aggregate the features of neighboring nodes, forming the drug's perturbation embedding.	The model structure consists of two autoencoders (encoder-decoder pairs) that collaboratively learn to predict cellular responses. The encoders map the input cellular states and drug perturbations into a shared latent space, while the decoders reconstruct the perturbed and unperturbed cellular states. The model also includes cycle consistency and adversarial learning components to ensure that the learned mappings are accurate and generalizable.	Bioinformatics	Predicting single-cell cellular responses to perturbations using cycle consistency learning	10.1093/bioinformatics/btae248	The baseline includes VAEs and GNNs, which lack cycle consistency, a gap addressed by cycleCDR's robust and interpretable mappings.
scVIDR	2023	Yes	Predict perturbations across untested cell types and doses	The scVIDR model uses a variational autoencoder (VAE) to embed single-cell gene expression data into a low-dimensional latent space, capturing the essential features of the cellular transcriptomic state. This latent space is then used to model the effects of chemical perturbations (scVIDR)	The model incorporates perturbation information by using vector arithmetic in the latent space to estimate the changes in gene expression due to chemical exposure. It employs a regression-based method to adjust for cell-type-specific differences in response to the perturbations (scVIDR).	The scVIDR model is based on a variational autoencoder architecture with additional components for regression-based adjustment and interpolation in the latent space. It includes an encoder that maps input data to a latent space and a decoder that reconstructs gene expression data, with specific enhancements to handle dose-response predictions (scVIDR).	Patterns	Generative modeling of single-cell gene expression for dose-dependent chemical perturbations	10.1016/j.patter.2023.100817	A computational framework outperforms random predictions in accuracy for single-cell RNA-seq data perturbation modeling.

Unagi	2023	No	Captures the complex cellular dynamics underlying disease progression, enhancing drug perturbation modeling and discovery.	The model embeds expression data by using a Variational Autoencoder-Generative Adversarial Network (VAE-GAN) architecture. It processes single-cell data into a low-dimensional latent space while accounting for the zero-inflated and noisy nature of such data through graph convolutional layers	Perturbation information is embedded by leveraging the learned latent space representations to simulate drug impacts. UNAGI manipulates the latent space informed by real drug perturbation data from the CMAP database, allowing for the simulation of drug effects on cellular states.	The model structure of UNAGI includes a VAE-GAN framework paired with graph convolutional layers. It also involves an iterative process that refines cell embeddings based on disease-informed gene markers, followed by in-silico perturbation analysis to predict therapeutic efficacy		Unagi: Deep Generative Model for Deciphering Cellular Dynamics and In-Silico Drug Discovery in Complex Diseases	10.21203/rs.3.rs-3676579/v1	Unagi is compared with traditional methods for predicting drug responses across cell types, addressing limitations of VAEs and scGen.
CINEMA-OT	2023	No	CINEMA-OT is a causal framework for perturbation effect analysis to identify individual treatment effects and synergy at the single cell level.	The model embeds expression data by applying independent component analysis (ICA) to decompose the data into confounding and treatment-associated signals. It then uses optimal transport to match cells across conditions, generating a causal matching plan that reflects counterfactual cell pairs.	Perturbation information is embedded through the identification of treatment-associated factors using ICA and by calculating the individual treatment effects (ITE) based on the optimal transport matching. This allows the model to capture the causal perturbation responses and perform detailed analysis on the treatment effects.	The model structure of CINEMA-OT involves an ICA-based approach to separate confounding factors from treatment effects, followed by the application of optimal transport for matching cells across conditions. This is supported by a deep learning framework that includes variational autoencoders (VAEs) and generative adversarial networks (GANs) to manage diverse data distributions and to ensure the synthetic quality of the representations.	Nature Method	Causal identification of single-cell experimental perturbation effects with CINEMA-OT	10.1038/s41592-023-02040-5	CINEMA-OT is compared with differential-abundance methods for synergy analysis and response clustering in high-throughput experiments.
ChemCPA	2023	No	Predict cellular responses to novel drug perturbations at a single-cell resolution	The model embeds expression data by using an encoder-decoder architecture that maps measured gene expression states to a latent vector space.	Embedded by combining the latent vector representation of the basal state with additive attribute embeddings. Specifically, the model uses a	The model structure of chemCPA consists of three main parts: (1) the gene expression encoder and decoder, (2) the attribute embedders, and (3) the adversarial classifiers. The encoder maps gene	NeurIPS 2022	Predicting Cellular Responses to Novel Drug Perturbations at a Single-Cell Resolution	10.48550/arXiv.2204.13545	A baseline model predicts drug responses using gene expression data, focusing on gene expression changes due to drugs.

				This is achieved through a multi-layer perceptron (MLP) that encodes the gene expression data into a lower-dimensional latent space.	perturbation network that consists of a molecule encoder, a perturbation encoder, and a dosage scaler to generate embeddings for drugs and dosages.	expressions to a latent space, the attribute embedders translate drug and cell line information into embeddings, and the adversarial classifiers ensure that the latent space representations are invariant to the attributes, thus providing disentangled representations.				
DREEP	2023	No	Predicting drug response from single-cell expression profiles of tumours	DREEP uses gf-icf normalization to extract the top relevant genes from each cell. These gene sets are then used as input for Gene Set Enrichment Analysis (GSEA) against each Genomic Profiles of Drug Sensitivity (GPDS) ranked-list to predict the sensitivity of a cell to a specific drug.	The model embeds perturbation information by correlating the expression of each gene with the potency of a drug across cell lines. It then constructs GPDS ranked lists where the degree of Pearson correlation coefficient reflects the gene's importance in predicting the effect of a small molecule.	The model structure involves normalizing single-cell RNA-seq data, performing GSEA for each cell against GPDS ranked lists, and estimating enrichment scores to determine drug sensitivity. It also includes a process for converting single-cell predictions to cell-line level predictions and for comparing predicted drug responses with experimental data to validate its accuracy.	BMC Medicine	Predicting drug response from single-cell expression profiles of tumours	10.1186/s12916-023-03182-1	Traditional machine learning algorithms predict drug responses using cancer cell line gene expression, with limitations in heterogeneity.
ontoVAE	2023	No	OntoVAE can perform predictive modeling, simulating the effects of genetic or drug-induced perturbations.	Expression data is embedded through the encoder part of the VAE, which compresses the high-dimensional input data into a lower-dimensional latent space. The decoder then attempts to reconstruct the input data from this latent space representation.	Perturbation information is embedded by modulating the values of input features in silico and monitoring how these changes propagate through the network. This allows OntoVAE to simulate the effects of drug treatment or genetic alterations.	OntoVAE has a multi-layer, sparse decoder that reflects the structure of the incorporated ontology. The latent space represents the root layer of the ontology, and each subsequent layer of the decoder corresponds to a depth level of the ontology, with terms becoming more specific towards the reconstruction layer made up of genes. The model uses skip connections to allow information flow across layers, similar to DenseNet, and restricts decoder weights to be positive to preserve	Bioinformatics	Biologically informed variational autoencoders allow predictive modeling of genetic and drug-induced perturbations	10.1093/bioinformatics/btad387	Models like Tybalt, LDVAE, and VEGA improve VAE interpretability but struggle with complex biological networks; OntoVAE improves interpretability.

						directionality.				
CellOT	2023	No	Designed to learn the response of individual cells to a given perturbation by mapping unpaired distributions of control and perturbed cells. It uses the theory of optimal transport and input convex neural architectures to predict the perturbed state of cells.	The expression data is embedded by applying CellOT to latent representations learned by an autoencoder, which helps capture the complex, high-dimensional nature of the data while preserving the cellular heterogeneity	Perturbation information is embedded by learning optimal transport maps that align control and perturbed populations, where these maps capture the minimal changes required to transition cells from their unperturbed to perturbed states	The model structure involves input convex neural networks (ICNNs) that parameterize the dual potentials of the optimal transport problem. CellOT uses a pair of ICNNs to learn the optimal transport map, which is then used to predict the perturbed state of cells based on their control state. The model is trained using an alternate max-min optimization strategy	Nature Method	Learning single-cell perturbation responses using neural optimal transport	10.1038/s41592-023-01969-x	Autoencoder-based models like cAE and scGen, and the identity baseline, are compared for simulating perturbations and capturing non-linear data structures.
CellOracle	2023	No	CellOracle is a python library for in silico gene perturbation analyses using single-cell omics data and Gene Regulatory Network models.	CellOracle embeds expression data by constructing cell-type- or cell-state-specific GRN configurations using cluster-wise regularized linear regression models with multi-omics data. It integrates single-cell RNA sequencing (scRNA-seq) and single-cell assay for transposase-accessible chromatin using sequencing (scATAC-seq) data to generate these configurations.	The model embeds perturbation information by simulating the global downstream shifts in gene expression following knockout (KO) or overexpression of TFs. It propagates the shift in gene expression through the GRN models to estimate the broad, downstream effects of TF perturbation.	The model structure of CellOracle consists of a machine-learning-based approach that integrates multimodal data to build custom GRN models. It uses a combination of computational perturbation with GRN modeling, which includes constructing GRNs, simulating gene expression shifts, estimating cell-identity transition probabilities, and converting these into a low-dimensional vector representation for intuitive visualization.	Nature	Dissecting cell identity via network inference and in silico gene perturbation	10.1038/s41586-022-05688-9	The baseline model assumes no perturbation or linearly propagates perturbation effects to predict gene expression changes.

scGEN	2019	Yes	Based on the unperturbed and perturbed data of another cell, the unperturbed data of that cell, predict the genes expression of that cell. Only can predict one perturbation.	A variational autoencoder model was used to encode single-cell gene expression data. The presentation data is mapped to a low-dimensional latent space	By encoding the perturbed cell data and the unperturbed cell data into the latent space, the perturbation vector can be calculated.	The model takes VAE as the basis model, and the gene information is input into the model encoder, and the project gene expression measurements into a latent space. The perturbation vector can be obtained by subtracting the latent representation of unperturbed cells from the latent representation of perturbed cells. The decoder can turn the latent representation of perturbed cells into gene expression.	Nature Method	scGen predicts single-cell perturbation responses	10.1038/s41592-022-00534-z	GANs, CVAEs, and style transfer GANs face challenges predicting out-of-sample responses due to data complexity.
scDiff	2023	No	The model mainly used to recover the clean single cell gene expression given the corrupted signal with added Gaussian noise to time step t.	use a linear mapping to project the noised gene expression into and further mix it with the sinusoidal time embedding	Used conditioners to extract a set of numerical representations of an input condition.	scDiff follows a general encoder-decoder design and consists of four main components: (1) input expression embedder; (2) various conditioners,where each converts a specific condition of the input cell into a sequence of dense numerical vectors; (3) a cross-attention encoder, which combines the input embeddings with the corresponding conditioners and transforms them into the hidden representation of the input cell; and (4) a linear decoder, that projects the hidden representation back to the input cell's noise free gene expression	bioRxiv	A General Single-Cell Analysis Framework via Conditional Diffusion Generative Models	10.1038/s41592-023-01202-4	ACTINN, DCA, and GEARS are task-specific methods for cell annotation, missing value imputation, and perturbation prediction.
ContrastiveVI	2023	No	The model can decompose the variations into shared and target-specific factors of variation	A shared encoder network transforms a cell into the parameters of the posterior distribution for a low-dimensional set of latent factors, which shares across	A second encoder encodes target data points into the parameters of the posterior distribution for a second set of latent factors encoding variations, which enriches in the target	The model is based on VAE, it has two encoders, one is shared encoder, which encodes shared latent variables, the other is a target encoder, which can encode target varibles. The decoder can regenerate backgroud and target information.	Nature Method	Isolating salient variations of interest in single-cell data with contrastiveVI	10.1038/s41592-023-01955-3	ContrastiveVI models are compared with treatment-based models but struggle to separate treatment-induced effects.

				target and background data.	dataset and not present in the background.					
samsVAE	2024	No	SAMS-VAE (Sparse Additive Mechanism Shift Variational Autoencoder) uses a sparse additive mechanism to generate perturbation data of cells.	The gene expression data is compressed into a low-dimensional potential space using an encoder.	model perturbations as inducing sparse latent offsets	The model is based on VAE, it has a encoder, which encodes the unperturbed cell's data into latent representation z^b. And the model models perturbations as inducing sparse latent. Offsets z^p. Input the sum of z^b and z^p into decoder, we can get the perturbed data.	arXiv	Modelling Cellular Perturbations with the Sparse Additive Mechanism Shift Variational Autoencoder	10.1038/s41592-022-01201-2	Standard VAEs lack the ability to capture sparse, global intervention effects, unlike the proposed SAMS-VAE.
STAMP	2024	No	The model can predict single/multiple genetic perturbation outcomes, genetic outcomes across cell lines, identify key regulatory genes and pathways on small samples and reveal precise genetic interactions.	gene embeddings derived from large single-cell foundational models or gene encoders	Given a perturbation set, STAMP retrieves the gene embedding of each element of that set	The model is consist of three pars, each part can solve one subtask. By resolving three progressive subtasks in a divide-and-conquer manner, we can do genetic perturbation prediction	Nature computational science	Toward subtask decomposition-based learning and benchmarking for genetic perturbation outcome prediction and beyond	10.1038/s43588-024-00698-1	Traditional genetic perturbation models fail with complex multi-perturbation scenarios compared to Biolord.
Biolord	2024	Yes	this model predicts cellular response to unseen drugs and genetic perturbations based on disentangling single-cell data	The encoder of model can respectively embed known attributes and unknown attributes into deomposed latent space	The encoder of model can respectively embed known attributes and unknown attributes into deomposed latent space	biolord's encoder encodes each attribute separately along with a single encoding of the unknown attributes. These define a decomposed latent space. And take the ecomposed latent space as input, the generative module provides measurement predictions.	Nature Biotech	Disentanglement of single-cell data with biolord	10.1038/s41587-023-02079-x	Traditional models struggle with multi-omic data and predict unseen conditions less effectively than Biolord.
Pdgrapher	2024	No	PDGRAPHER predicts a candidate set of therapeutic targets to shift cell gene expression from diseased to treated state.			PDGRAPHER has a perturbagen discovery module to computes a candidate set of therapeutic targets,and a response prediction module to predicts the response of the diseased sample to the predicted candidate perturbagen.	bioRxiv	Combinatorial prediction of therapeutic perturbations using causally-inspired neural networks	10.1038/s41592-024-00679-3	Target-driven drug discovery models are limited in capturing phenotypic responses to compounds.

TAT	2024	No	The model is used to predict compound activity.(transcriptomics-to-activity Transformer (TAT) models that map gene-expression profiles attained over compound treatment at multiple concentrations in a single profiling assay to compound activity in other dose-response assays)	The model projects linearly the gene-expression outcomes at individual concentrations in an input multiconcentration profile onto a learned 512-D feature space. After injecting the positional information that implicitly encodes the compound concentration, the resulting concentration-informed gene-expression embeddings.	positional informationimplicitly encodes the compound concentration	The model take Multi-concentration transcriptomic profile as input, projects linearly the gene-expression outcomes at individual concentrations, injects positional information encoding the concentration information, and feeds the resulting vectors onto a Transformer encoder. It averages the transformed tokens and projects the mean embedding onto a pAC50(the negative logarithm of the half-maximal activity concentration) prediction.	Journal of Chemical Information and Modeling	Compound Activity Prediction with Dose-Dependent Transcriptomic Profiles and Deep Learning	10.1021/acs.jcim.3c01855.	/
scVAE	2024	No	The model used to predict likelihood parameters for modeling gene expression levels and perturbation labels.	the background encoder encode the cell variations present in the control cells to a low-dimensional random vector.	Salient encoder encoder variations due to perturbations	The model incorporates two distinct encoders: a background encoder, capturing biological attributes and a salient encoder, specifically targeting perturbation effects. For generative part, both latent embeddings are fed into a decoder that outputs the likelihood parameters for modeling gene expression levels. Based on the salient embedding, a perturbation classifier discerns perturbation labels.	arXiv	A Supervised Contrastive Framework for Learning Disentangled Representations of Cell Perturbation Data	10.1038/s41592-024-00716-3	Noise-contrastive estimation (NCE) and InfoNCE are baseline contrastive loss methods that assume specific data distributions.
Cell PaintingCNN	2024	No	learn representations from cell images to study the effects of different perturbations on cells	The proposed model mainly deals with phenotypic data based on cell microscopic images, rather than directly dealing with gene expression data.		deep convolutional neural network architecture used in Cell Painting CNN is the EfficientNet, which trained to extract features from single cells	Nature Communications	Learning representations for image-based profiling of perturbations	10.1038/s41467-024-45999-1	Image-based models for cellular phenotypic effects struggle to account for confounding factors in learned representations.

scDisInFact	2024	No	This method can be used to do batch effect removal, condition-associated key gene detection, and perturbation prediction	scDisInFact effectively disentangles the gene expression data into the shared biological factors (shared-bio factors), unshared biological factors (unshared-bio factors), and technical batch effect	An unshared encoder learns unshared-bio factors, which represent biological variations that are related to the condition effect	scDisInFact uses an encoding network to learn the disentangled latent factors, and uses a decoding network to generate gene expression data from the latent factors.	Nature Communications	scDisInFact: disentangled learning for integration and prediction of multi-batch multi-condition single-cell RNA-sequencing data	10.1038/s41467-024-45227-w	Batch effect removal and perturbation prediction methods fail to distinguish between technical and biological effects.
CellCap	2024	No	CellCap uncovers the relationship between cell state and perturbation response	CellCap uses a multi-layer neural network to project the observed gene count matrix into a “basal state”.	The perturbation embedding is related to the usage amplitude of response programs, which are represented as a matrix where each row corresponds to a distinct response program. The values in the matrix range from (−1, 1), and the usage amplitude of each response program needs to be inferred.		bioRxiv	Modeling interpretable correspondence between cell state and perturbation response with CellCap	10.1038/s41592-024-01271-2	Traditional perturbation response models fail to capture the complexity and interpretability of cell states and perturbations.
VEGA	2021	No	See if the cells have a significant response before and after perturb	VEGA consists of a deep nonlinear encoder and a masked linear decoder representing single-cell transcriptome data to a low-dimensional interpretive latent space.		The model takes VAE as the basis model. By encoding single-cell transcriptomics data,we can get a lower-dimensional interpretable latent space z that approximates a set of user-supplied gene modules.By comparing latent space in different cell states, it is possible to determine whether perturbation has an effect on cells	Nature Communications	VEGA is an interpretable generative model for inferring biological network activity in single-cell transcriptomics	10.1038/s41467-021-26017-0	VEGA improves biological network analysis interpretability in single-cell transcriptomics using gene annotations.
trVAE	2019	Yes	Based on the unperturbed and perturbed data of another cell, the	Input randomized batches of data and condition into encoder, it can	Transforming the condition (unperturbed-> perturbed), generate	The decoder part has an MMD layer. When decoding, transform batches of the source condition to obtain the	arXiv	Conditional out-of-sample generation for un paired data	10.48550/arXiv.1910.01791	trVAE applies maximum mean discrepancy (MMD) in its

			unperturbed data of that cell, predict the genes expression of that cell. Can use forpredicting different pertubations.	generate the latent representation for this data.	the latent representation for perturbed data.	data after cell disturbance		using trVAE		decoder layer for better generalization in out-of-sample generation.
sVAE+	2023	No	Based on the unperturbed and perturbed data of another cell, the unperturbed data of that cell, predict the genes expression of that cell. Can use forpredicting different pertubations.	Input gene expression profiles of cells under different genetic or chemical perturbations, as well as the intervention label to encorder, we can get latent representation of different intervention.	Input gene expression profiles of cells under different genetic or chemical perturbations, as well as the intervention label to encorder, we can get latent representation of different intervention.	Survival related features are extracted from the latent variables generated by the encoder, and survival time and event probability are predicted by the survival module. The decoder can turn the latent representation of perturbed cells into gene expression.	2nd Conference on Causal Learning and Reasoning	Towards Causal Representation Learning	10.48550/arXiv.2102.11107	scRank predicts drug-responsive cell types from scRNA-seq data using gene regulatory network models for precision therapy.
graphVCI	2023	No	Based on the unperturbed and perturbed data of another cell, the unperturbed data of that cell, predict the genes expression of that cell. Can use forpredicting different pertubations.	Input graph G (e.g. gene relation graph), outcome Y (e.g. gene expressions), covariates X (e.g. cell types, donors, etc.) and treatment T (e.g. drug perturbation) into encoder, it can generate the latent representation for this data.	Input graph G (e.g. gene relation graph), outcome Y (e.g. gene expressions), covariates X (e.g. cell types, donors, etc.) and treatment T (e.g. drug perturbation) into encoder, it can generate the latent representation for this data.	For predicting gene expresions, the model take graph G (e.g. gene relation graph), outcome Y (e.g. gene expressions), covariates X (e.g. cell types, donors, etc.) and treatment T (e.g. drug perturbation) as input of encoder,and generate latent representation. Then input latent representation and treatment to decorder, it can generate gene expressions.	ICLR 2023	Predicting Cellular Responses with Variational Causal Inference and Refined Relational Information	10.48550/arXiv.2210.00116	A graph variational Bayesian causal inference model enhances drug discovery and personalized medicine using gene regulatory networks.
scRank	2024	No	Input single-cell transcriptome data and direct drug targets of interest to generate a drug-reactive cell type ranking		Input single-cell transcriptomic data and drug direct targets of interest. It will reconstruct the gene regulatory network from the expression profiles.(GRN), simulate the effect of the insilico drug perturbation in the GRN. (tGRN), and estimate the		Cell Reports Medicine	scRank infers drug-responsive cell types from untreated scRNA-seq data using a target-perturbed gene regulatory network	10.1016/j.xcrm.2024.101568	This paper combines machine learning and graphical causality to uncover high-level causal variables for transfer learning and generalization.

					perturbation. By compare the perturbation score, it can generate drug-responsive cell-type rank.					
Gears	2023	Yes	Predict the outcomes of genetic perturbations, including those that have never been experimentally tested, and identify genetic interactions such as synergies and suppressions	Single-cell RNA sequencing data is used to capture the transcriptional responses of genes in perturbed conditions.	GEARS uses a knowledge graph of gene-gene relationships combined with deep learning to simulate perturbation outcomes.	The model architecture involves integrating a deep learning approach with a knowledge graph for gene activation and repression, along with a graph-based neural network for learning gene interactions.	Nature Biotechnology	Predicting transcriptional outcomes of novel multigene perturbations with GEARS	10.1038/s41587-023-01905-6	GEARS outperforms existing methods in predicting transcriptional outcomes, especially for multigene perturbations.
scFoundation	2023	Yes	The paper focuses on developing a large-scale model that can generalize across single-cell transcriptomic data and predict gene expression patterns.	Single-cell RNA sequencing data, which is embedded using advanced machine learning techniques for better representation of gene expression profiles.	Although not explicitly stated, the model can likely be applied to data involving perturbations based on its scalability to various conditions and datasets.	The model architecture is based on a foundation model approach, which leverages large-scale data to generalize across single-cell RNA-seq experiments, using state-of-the-art neural networks.	Nature Biotechnology	Large-Scale Foundation Model on Single-cell Transcriptomics	10.1101/2023.05.29.542705	The foundation model scales better and predicts gene expression more accurately than traditional single-cell transcriptomics methods.
scGPT	2024	Yes	The research focuses on constructing and applying a foundation model (scGPT) to single-cell multi-omics data to advance genetic and cellular biology research.	Single-cell sequencing data, representing gene expression across different cell types, is embedded using GPT-like transformers.	The model can likely be extended to perturbation data through generative modeling of cellular responses, though specific methods are not fully detailed in the extract.	The architecture is based on large pre-trained transformers (similar to GPT models) applied to single-cell sequencing data for building a generalized, scalable model.	Nature Methods	scGPT: Toward Building a Foundation Model for Single-cell Multi-omics Using Generative AI	10.1038/s41592-024-02101-0	scGPT generalizes across diverse multi-omics datasets more efficiently than existing single-cell analysis methods.
scELMo	2024	Yes	The study develops and applies scELMo to analyze single-cell RNA sequencing data, aiming to demonstrate that LLM-generated embeddings can improve the analysis of single-cell gene expression data.	scELMo uses embeddings from large language models, adapted to represent the gene expression profiles of single cells in a way that preserves their biological context.	The method likely incorporates perturbation data indirectly by learning representations from large datasets, though the exact perturbation-specific details are not elaborated in the	scELMo adapts the architecture of pre-trained language models to the task of single-cell RNA sequencing data analysis. It uses a generative pre-training approach similar to standard LLMs.	Nature Methods	scELMo: Embeddings from Language Models are Good Learners for Single-cell Data Analysis	10.1101/2023.12.07.569910	scELMo's LLM-based embeddings outperform traditional models for specific single-cell data analysis tasks.

					provided text.					
gene compass	2024	No	The primary task is to develop and apply the GeneCompass model to uncover universal gene regulatory mechanisms that span different species.	The model uses large-scale gene expression data, including multi-species datasets, to inform and enhance its predictions of gene regulatory relationships.	Although the model is primarily designed to study gene regulation, perturbations across species are likely considered in its analysis to better capture the impact of regulatory changes.	GeneCompass is a knowledge-informed foundation model that combines biological data with machine learning techniques. The architecture leverages both inter-species genetic information and deep learning approaches to model gene regulation.	Nature Methods	GeneCompass: Deciphering Universal Gene Regulatory Mechanisms with a Knowledge-informed Cross-species Foundation Model	10.1038/s41422-024-01034-y	GeneCompass integrates multi-species data for improved scalability and accuracy
scBERT	2022	No	The main task is to develop and apply scBERT for annotating cell types in single-cell RNA-seq data, overcoming the issues present in existing annotation methods.	Single-cell RNA-seq data is embedded using the scBERT model, which leverages transformer-based bidirectional encoder representations.	While not explicitly detailed, scBERT's architecture can likely be adapted to perturbation data by considering gene-gene interactions and handling batch effects in a more robust manner.	The model architecture is based on BERT, a transformer-based approach, and is tailored for single-cell RNA-seq data, enabling more accurate cell type annotations by learning from large-scale data.	Nature Machine Intelligence	scBERT as a Large-Scale Pretrained Deep Language Model for Cell Type Annotation of Single-cell RNA-seq Data	10.1038/s42256-022-00534-z	scBERT addresses limitations in data curation and gene interaction analysis, offering enhanced annotation capabilities.
Geneformer	2024	No	The research focuses on developing and applying Geneformer, a transfer learning-based model, to map gene networks and make predictions in settings with limited data.	Gene expression data is embedded through the use of transfer learning models that are pre-trained on general datasets and fine-tuned for specific downstream tasks in gene network prediction.	Transfer learning allows the model to use general biological data to predict the impact of perturbations in gene networks, especially in cases where perturbation-specific data is scarce.	Geneformer is based on transfer learning, utilizing pre-trained deep learning models for general biological datasets, and then fine-tuning these models for specific tasks related to network biology.	Nature	Geneformer: Transfer Learning Enables Predictions in Network Biology	10.1038/s41586-023-06139-9	Geneformer leverages transfer learning and pre-trained datasets to perform well even with limited data.
UCE	2024	No	The paper focuses on developing SATURN to learn universal cell embeddings that combine RNA expression with protein embeddings from language models, allowing the	RNA-seq expression data is embedded by combining it with protein embeddings obtained from language models, which represent biological properties of genes.	Although not explicitly stated, SATURN can likely be extended to perturbation data by integrating perturbation effects with RNA and protein embeddings.	SATURN uses deep learning techniques that combine protein embeddings from language models with RNA expression data to create universal cell embeddings that are independent of species-specific genomic differences.	Nature Methods	Toward Universal Cell Embeddings: Integrating Single-cell RNA-seq Datasets Across Species with	10.1038/s41592-024-02191-z	SATURN effectively integrates cross-species RNA-seq datasets, overcoming species-specific differences and improving data

			integration of single-cell datasets from different species.					SATURN		harmonization.
cellPLM	2024	No	The paper develops CellPLM, a new pre-trained model, to address challenges in scRNA-seq data analysis, including learning cell-cell relationships and handling the noisy, limited nature of single-cell datasets.	CellPLM embeds single-cell RNA-seq data by treating cells as tokens and tissues as sentences. It also incorporates spatially resolved transcriptomic data to facilitate the learning of cell-cell relationships.	The model's design allows it to integrate perturbation data by focusing on learning cell-cell relationships, which is key in analyzing responses to perturbations.	The architecture involves treating cells as tokens and tissues as sentences, similar to language models, but adapted to the unique structure and challenges of single-cell data.	Nature Methods	CellPLM: Pre-training of Cell Language Model Beyond Single Cells	10.1101/2023.12.07.569910	CellPLM improves scRNA-seq analysis by learning cell-cell relationships more effectively.
scPRAM	2024	Yes	Predicting single-cell gene expression perturbation responses across cell types, species, and individuals using attention mechanisms. Focuses on capturing heterogeneity in perturbation responses and predicting entire perturbation response distributions rather than just average responses.	Uses a Variational Autoencoder (VAE) to encode high-dimensional, sparse cell-gene expression matrices into a lower-dimensional latent space.	Employs optimal transport based on Sinkhorn algorithm to align and match cells before and after perturbation in the latent space. Uses Euclidean distance to calculate cost matrix between pre- and post-perturbation cell states, then finds optimal coupling matrix M to pair unpaired cells and compute perturbation vectors.	Three-component architecture: (1) VAE for encoding cell expression data into latent space, (2) Optimal transport (Sinkhorn algorithm) for matching unpaired pre- and post-perturbation cells, and (3) Attention mechanism that uses test cell latent vectors as queries, training set latent vectors as keys, and perturbation vectors as values to compute weighted perturbation predictions.	Bioinformatics	scPRAM accurately predicts single-cell gene expression perturbation response based on attention mechanism	10.1093/bioinformatics/btae265	Compared against scGen, trVAE, scVIDR, scPreGAN, and CellOT
Squidiff	2025	Yes	Predict single-cell transcriptomic changes across diverse biological scenarios, including cell differentiation, gene perturbations, drug responses, and physical stimuli, enabling interpolation of transient cell states and counterfactual condition modeling.	Uses a semantic encoder neural network to map single-cell gene expression profiles into a continuous latent semantic space that captures biologically meaningful cell states and state transitions.	Encodes perturbations as directions in the semantic latent space, enabling perturbation effects to be modeled through latent interpolation or vector arithmetic; optionally incorporates drug structural embeddings for unseen compounds.	A conditional diffusion-based generative model combining a semantic encoder and a denoising diffusion implicit model (DDIM) to generate transcriptomic profiles via iterative denoising.	Nature Methods	Squidiff: predicting cellular development and responses to perturbations using a diffusion model	10.1038/s41592-025-02877-y	Squidiff is evaluated against VAE- and graph-based perturbation models (e.g., scGen, GEARS, PRNet) for predicting perturbation responses and developmental trajectories.

Table S3.
List of 22 drugs used in the Perturb_KHP dataset.

	drug	final concentration for K562	final concentration for HDFs	final concentration for PBMCs	product number	company
1	Rapamycin (Sirolimus)	1nM	10nM	10nM	S1039	Selleck
2	Metformin HCl	1mM	100uM	100uM	S1950	Selleck
3	Spermidine	10uM	10uM	10uM	S3569	Selleck
4	Nicotinamide Riboside Chloride	1uM	100uM	100uM	S2935	Selleck
5	β-Nicotinamide Mononucleotide	1mM	100uM	100uM	S5259	Selleck
6	Fisetin	1uM	1uM	1uM	S2298	Selleck
7	Dasatinib	0.001nM	0.1 nM	0.1 nM	S1021	Selleck
8	Quercetin	2uM	10uM	10uM	S2391	Selleck
9	Everolimus (RAD001)	50nM	10nM	10nM	S1120	Selleck
10	Resveratrol	1uM	10uM	1uM	S1396	Selleck
11	Navitoclax (ABT263)	10nM	100nM	10nM	S1001	Selleck
12	Canagliflozin (SGLT2 inhibitor)	2uM	2 uM	2 uM	S2760	Selleck
13	urolithin A	500nM	500nM	1 uM	S5312	Selleck
14	Alpha-Ketoglutarate	0.5mM	0.5mM	0.5mM	S6237	Selleck
15	SRT2104	2uM	2uM	2uM	S7792	Selleck
16	Adezmapimod (SB203580)	1uM	10uM	1uM	S1076	Selleck
17	Taurine	1mM	1mM	1mM	T8691	Sigma
18	Sodium Butyrate	50uM	100uM	100uM	303410	Sigma
19	FOXO4-DRI	1uM	1uM	1uM	HY-P4157	MCE
20	Platelet Factor 4 (PF4), CXCL4	2ug/ml	2ug/ml	2ug/ml	UA040186	UA.Bio
21	VPA	10uM	100uM	10uM	P4543	Sigma
22	BeiCell reprogramming cocktail	1:1 dilution in culture medium	1:1 dilution in culture medium	1:1 dilution in culture medium	BCTCiPS-001	BeiCell

Table S4.

List of 111 drugs used in the Perturb_cmo_V1 dataset.

Rapamycin	Magnolol	Deferoxamine Mesylate	DL-α-Tocopherol	Doramapimod	Edaravone
Ridaforolimus	Isosteviol	Afatinib Dimaleate	CPI203	Glutathione	Celecoxib
Hesperidin	BX517	Seletalisib	Adenosine disodium triphosphate	Quercetin	PXD-101
Honokiol	Indole-3-carbinol	INO1001	Seclidemstat	SR9011	Losartan
SB203580	Berberine hydrochloride	Levobupivacaine HCl	TDZD8	Melatonine	Metformin hydrochloride
Resveratrol	Niclosamide	Berbamine	Dapagliflozin	Coenzyme Q10	PCI24781
Everolimus	Caffeic Acid Phenethyl Ester	Gomisin A	D--Trehalose dihydrate	Idebenone	Resminostat hydrochloride
AICAR	Dasatinib	Dexamethasone	Ibrutinib	AZD6482	Givinostat hydrochloride monohydrate
ZSTK474	Chlorogenic Acid	XL147 analogue	Pirfenidone	Fasudil hydrochloride	Navitoclax
Galunisertib	Loganin	SRT1720 hydrochloride	Berberine	Caffeic Acid	Ripasudil hydrochloride dihydrate
Tofacitinib	Bilobalide	Mocetinostat	Curcumin	Astaxanthin	Cladribine
Hesperetin	Artesunate	Balsalazide disodium salt dihydrate	CHIR99021	Empagliflozin	
Serabelisib	RG2833	Nafamostat mesylate	PCI34051	CPI169	
CC223	Apabetalone	AS8351	AR42	Antrapurol	
Arctigenin	Calcitriol	KU0063794	Sivelestat	Citarinostat	
PI103	BMS345541	SRT2104	EPZ005687	Sodium Phenylbutyrate	
Larotrectinib sulfate	Berberine hydrogen sulphate	Fluorouracil	Vemurafenib	GKT137831	
CNX1351	Irbinitinib	Acetylcysteine	Fingolimod hydrochloride	Esculetin	
Filgotinib	Valproic Acid	Taurine	FG2216	PF06409577	
4EGI1	PHT427	Rosiglitazone maleate	MSDC 0602	Sinomenine hydrochloride	

Table S5.

Top 5 and bottom 5 ranked drugs from the drug dataset based on TRADE calculations.

Srivatsan_sciplex3_sub10.h5ad	Burkhardt_sub10.h5ad	Tahoe100_sub10.h5ad	Parse_10M_PBMC_sub10.h5ad	Perturb_KHP_sub10.h5ad	Perturb_cmo_V1_sub10.h5ad
Quisinostat (JNJ-26481585) 2HCl	CEP-18770 (Delanzomib)	Ouabain (Octahydrate)	IL-32-beta	VPA	Givinostat hydrochloride monohydrate
Panobinostat (LBH589)	Ganetespib (STA-9090)	Abiraterone acetate	IFN-beta	SRT2104	AR42
Dacinostat (LAQ824)	LY2090314	Piroxicam	IFN-omega	FOXO4-DRI	Mocetinostat
YM155 (Sepantronium Bromide)	IN1451	Omeprazole (sodium)	IL-4	Adezmapimod	Dexamethasone
CUDC-907	Oprozomib (ONX 0912)	Atazanavir (sulfate)	IL-15	Rapamycin	PXD-101
SGI-1776 free base	Palbociclib	Topotecan (hydrochloride)	C3a	CXCL4	Losartan
Ki8751	Linagliptin	Naproxen	TPO	Quercetin	Loganin
Altretamine	Penfluridol	Cytarabine	IL-36-alpha	Canagliflozin	Fasudil hydrochloride
Nintedanib (BIBF 1120)	Porcn Inhibitor III	Regorafenib	IL-26	urolithin A	SR9011
Regorafenib (BAY 73-4506)	LDN 193189	DTP3	TRAIL	Sodium Butyrate	PHT427
